# Hypomethylated interferon regulatory factor 8 recruits activating protein-2α to attenuate porcine epidemic diarrhea virus infection in porcine jejunum

**DOI:** 10.3389/fimmu.2023.1187144

**Published:** 2023-08-01

**Authors:** Qiufang Zong, Huan Qu, Xianrui Zheng, Haifei Wang, Shenglong Wu, Zongjun Yin, Wenbin Bao

**Affiliations:** ^1^ College of Animal Science and Technology, Yangzhou University, Yangzhou, Jiangsu, China; ^2^ College of Animal Science and Technology, Anhui Agricultural University, Hefei, Anhui, China

**Keywords:** PEDV, IRF8, DNA methylation, AP-2α, oxidative stress, apoptosis

## Abstract

Interferon regulatory factor 8 (*IRF8*) is a key regulator of innate immune receptor signaling that resists pathogen invasion by regulating cell growth and differentiation. Porcine epidemic diarrhea virus (PEDV) targets the intestine and damages the mucosal barrier. However, whether *IRF8* regulates PEDV replication remains unclear. We revealed that PEDV infection activated *IRF8* expression. Moreover, *IRF8* deletion drastically promoted PEDV replication and invasion, increasing the virus copies and titers. Hypomethylation enrichment of activating protein (AP)-2α was significantly negatively correlated with high *IRF8* expression, and AP-2α directly targeted the *IRF8* promoter to regulate PEDV replication. Furthermore, *IRF8* overexpression decreased the cellular reactive oxygen species levels and mitochondrial membrane potential and increased the antioxidant enzyme activities to alleviate PEDV-induced oxidative damage. *IRF8* overexpression suppressed apoptotic gene expression, thereby inhibiting apoptosis in response to PEDV stimulation. Taken together, this study demonstrates that AP-2α is involved in PEDV-induced epigenetic modification of *IRF8* to reduce cell apoptosis and oxidative stress and facilitate host resistance to PEDV in the intestinal epithelium.

## 1 Introduction

Porcine epidemic diarrhea (PED) is a severe intestinal and severe respiratory infectious disease characterized by the atrophy and blockage of intestinal villi, with typical symptoms of watery diarrhea, vomiting, and dehydration in sick pigs, which may rapidly lead to the death of affected piglets (1–3). PED is a devastating intestinal disease that infects pigs of different ages, breeds, and genders, causing serious economic loss to the pig industry (4). PED virus (PEDV) is an enveloped RNA virus of the *Coronavirus* genus (5). PEDV enters the intestine via oral and nasal infections, mainly proliferates in the intestinal villous epithelial cell, causes malabsorption of nutrients, and leads to diarrhea (6). Therefore, keeping the regular function of the intestinal barrier is crucial to prevent PEDV invasion.

PEDV infection may disrupt the interferon (IFN) response (7). Activation of IFN regulators is critical for controlling the expression of IFN and several IFN-induced genes (8). IFN regulatory factor 8 (IRF8), a key regulator for IFN and IFN-induced genes (9, 10), is involved in the clearance of virus-infected cells and regulation of cell growth, differentiation, and survival (11, 12). IRF8 is also a vital regulator of the immune response to various pathogenic infections. IRF8 regulates caspase activation and subsequent KAP1 cleavage in the Epstein-Barr viruses (13). Host cells have been reported to perform an active role in resisting PRRSV infection through the IRF8–microRNA-10a–signal recognition particle 14 regulatory pathway (14). *IRF8* has been reported to be a key candidate gene associated with PEDV resistance in pigs via epigenetic analysis (15). It’s worth mentioning that the expression of *IRF8* in PEDV-infected jejunum is 3.8-fold higher than Control group via RNA-seq analysis. In addition, the *IRF8* expression in PEDV-infected IPEC-J2 was 3.1-fold higher than those in the Control group, which is consistent with the expression trend at the molecular level (15). Therefore, *IRF8* is an important candidate gene for piglet resistance to PEDV infection that may be regulated by epigenetic modifications.

In recent years, advancements in epigenetics have facilitated the study of genetic mechanisms of various diseases. DNA methylation is a key epigenetic role in regulating gene transcription that introduces a monomethyl group on the 5^th^ carbon atom of cytosine to convert it into 5-methylcytosine (16, 17). DNA methylation is suggested to inhibit gene expression and cell differentiation (18) by affecting the binding of specific transcription factor (TF) to DNA. Silencing of *IRF8* in various tumors may be closely related to abnormal DNA methylation (19) due to the inability of the TF STAT1, in activating *IRF8* (20). However, whether *IRF8* transcription is mediated by DNA methylation during PEDV infection in IPEC-J2 remains unknown.

In this study, we aimed to demonstrate the inhibitory effects and potential action mechanisms of *IRF8* in intestinal tract infections caused by PEDV. By deletion and overexpression of *IRF8* and detection of viral copy, cell activity, and inflammatory factor expression, it was preliminarily verified that *IRF8* activation resists PEDV infection. Further analysis revealed that the transcription factor, activating protein (AP)-2α, targets *IRF8* promoter methylation and participates in PEDV resistance regulation. Pathway enrichment analysis indicated that *IRF8* was involved in anti-PEDV infection via apoptosis and oxidative stress pathways. Our results provide a basis for further research on the mechanisms of *IRF8* resistance and its application in PED resistance breeding.

## 2 Materials and methods

### 2.1 Ethics statement

All animal experiments were approved by the Institutional Animal Care and Use Committee (IACUC) of the Yangzhou University Animal Experiments Ethics Committee (permit number: SYXK (Su) IACUC 2012-0029). All experiments were performed following the relevant guideline.

### 2.2 Experimental animals

We used 6 piglets (7-day-old) exhibiting the symptom of vomiting, dehydration, and diarrhea and 6 healthy piglets under the same feeding conditions from a pig farm. After euthanizing with sodium pentobarbital, duodenum, jejunum, and ileum were frozen and stored for subsequent experiments.

### 2.3 Histomorphology of the pathogen

RNA was extracted from the duodenum, jejunum, and ileum tissues of 12 piglets and reverse-transcribed into cDNA for the PCR amplification of PEDV, transmissible gastroenteritis virus (TGEV), porcine delta coronavirus (PDCoV), and porcine rotavirus (PoRV). All primers are listed in **Table S1**. After washing the intestines using phosphate-buffered saline (PBS; Sangon, Shanghai, China) and fixing using 4% paraformaldehyde for 1 day. Then the sections were embedded in paraffin, baked at 60 °C, and stained with hematoxylin and eosin (HE). The morphology of the intestine in the Control and PEDV groups was observed under a light microscope. Images were analyzed using the analysis system (Motic, Xiamen, China).

### 2.4 Cell lines and culture condition

Porcine intestinal epithelial cell (IPEC-J2) was obtained from the China Agricultural University. Vero kidney cell (Vero) was purchased from the ATCC. Cells were maintained in DMEM/F12 (Gibco, NY, USA), which contained 5% FBS in a 37 °C incubator.

### 2.5 Virus titration and infection

CV777 strain of PEDV was provided by the China Agricultural University. Vero was used for PEDV propagating and titrating. IPEC-J2 was cultured overnight in a 12 well plate (Jet Biofil, Guangzhou, China) of 3 × 10^5^/mL. PEDV toxicity of 0.1 MOI was propagated in an FBS-free medium containing 8 μg/mL trypsin at 37°C for 2 h. After specific periods (24, 48, and 72 h) of viral infection, the cell was collected for further experiments and 50% tissue culture infectious dose (TCID_50_) was used for quantifying the infectious virus particles (21). To detect the viral titers in IRF8-KO, IRF8-OE, and Control cells, culture supernatant was collected after specific periods and titrated by the TCID_50_ method.

### 2.6 Construction of IRF8, Sp1, and AP-2α overexpression vectors

The coding sequence (CDS) region was amplified and ligated to the pcDNA3.1, vector using a T4 ligase. Subsequently, the recombinant vector was transformed into the Top-10 competent cells. The overexpression vectors were constructed and named IRF8-OE, Sp1-OE, and AP-2α-OE. After transfection into IPEC-J2 by Lipofectamine 2000 (Lipo2000) reagent and sieving with 400 µg/mL G_418_ for 7 days. Stable cell lines constituted the polyclonal pools of cells.

### 2.7 *IRF8* gene depletion *via* CRISPR/Cas9 editing

Single guide RNAs (sgRNAs) were designed by the CRISPR Design software (http://crispr.mit.edu/; **Table S2**). Oligo corresponding to sgRNA was annealed to dsDNA. DNA was ligated into the pGK1.2 vector. Subsequently, the recombinant vector was transformed into the Top-10 competent cells. Positive recombinant plasmids were extracted and transfected into IPEC-J2 by Lipo2000. After sieving with 3 μg/mL puromycin for 3 days, the DNA of puromycin-resistant cells was extracted for PCR amplification. Primers are listed in **Table S3**. Gene knockout sequences were detected via TA clone sequencing. Positive monoclonal cells were harvested and called IRF8-KO cells.

### 2.8 RNA isolation and quantitative PCR

Cellular and intestinal RNA was isolated by FastPure Cell/Tissue Total RNA Isolation Kit V2 (Vazyme, Nanjing, China). cDNA was synthesized using the Hifair II 1^st^ Strand cDNA Synthesis Kit (Yeasen, Shanghai, China). RT-qPCR amplification was performed using the StepOnePlus quantitative PCR system (ABI, CA, USA). Relative quantification results were calculated following the 2^−ΔΔCt^ method (22). Primers used here are listed in **Table S4**.

### 2.9 Cell viability assay

To demonstrate the effect of *IRF8* knockout and inhibitors (Z-VAD-FMK and BIP-V5) on IPEC-J2 proliferation, cells were subjected to a cell counting kit-8 (CCK-8) assay. IRF8-KO and Control cells were infected with PEDV at 24, 48, and 72 h. Z-VAD-FMK and BIP-V5 (0.1, 1, 2.5, 5, 7.5, 10, and 20 μM) were incubated with cells for 48 h. Then, 10 μL CCK-8 reagent (Vazyme, Nanjing, China) was added, and the absorbance of 450 nm was determined by microplate reader.

Control and IRF8-OE cell were seeded at 80% confluency for PEDV infection. After 48 h, cells were cultured with 100 μL live/dead reagent (Yeasen, Shanghai, China) for 30 min at 37°C. Fluorescence microscopy was used to capture the signals of Calcein AM, which represented the live cell and those of Propidium Iodide (PI), which represented the dead cell.

### 2.10 Flow cytometry assay

For apoptosis analysis, cells of PEDV and IRF8-OE+PEDV groups were collected and washed using the binding buffer (Solarbio, Beijing, China). Annexin V-FITC and PI were used to stain cells, and the apoptotic cell was subsequently detected by CytoFLEX flow cytometer. For the reactive oxygen species (ROS) assay, cells of Control, PEDV, IRF8-OE, and IRF8-OE+PEDV groups were harvested and suspended in diluted DCFH-DA (Solarbio) for 30 min at 37 °C. The ROS level was detected using a CytoFLEX flow cytometer after washing thrice with Opti-MEM. For the mitochondrial membrane potential (MMP) assay, cells of Control, PEDV, IRF8-OE, and IRF8-OE+PEDV groups were harvested and incubated with JC-1 dye working buffer (Beyotime, Shanghai, China) at 37 °C for 30 min. After washing twice with the JC-1 staining buffer, MMP was determined using flow cytometry.

### 2.11 Western blotting analysis

Cells were lysed for 20 min in RIPA buffer on ice. Cells were then centrifuged for 10 min at 12,000 rpm to remove the insoluble components. The concentration of protein was determined by a BCA Protein Assay Kit (CW Biotech, Beijing, China). Protein separation was conducted by SDS-PAGE and transformation by PVDF membrane (Millipore, MA, USA). After blocking with 5% skim milk for 1 h and incubating with the primary antibody (**Table S5**) at 4°C overnight. The membrane was then incubated with a secondary antibody for 1 h. Protein was visualized by Chemiluminescence Kit (Thermo Fisher Scientific, MA, USA), and the image was obtained by the FC3 Chemiluminescent system (ProteinSimple, CA, USA).

### 2.12 Total DNA isolation and bisulfite treatment

Genomic DNA from the jejunum was isolated by the TIANamp Genomic DNA Kit (Tiangen, Beijing, China). The DNA was bisulfite-converted following the instruction of the EZ Methylation-Gold Kit (Zymo Research, CA, USA). Porcine *IRF8* promoter (upstream 2000 bp) was acquired from the NCBI database. The CpG island of the *IRF8* gene was predicted by MethPrimer software. The primer for bisulfite sequencing PCR (BSP) amplification was listed in **Table S6**. The PCR system (50 µL) contains 2 µL forward primer (10 pmol/μL), 2 µL reverse primer (10 pmol/μL), 25 µL ZYMO Taq Premix, 4 µL DNA, and 17 µL H_2_O. Reaction condition: 95°C for 15 min, 45 cycles of 94°C for 40 s, 54/52°C for 40 s, 72°C for 1 min, and 72°C for 8 min. After purifying, the fragment was ligated with pMD-19T at 16°C for 12 h. Recombinant plasmids were transformed into *E. coli* DH-5αcell and cultured on an agar plate containing ampicillin at 37°C. 25 monoclonal colonies were sent to perform bisulfite sequencing 14 h later. The methylation profile of CpG sites was calculated by the QUMA database.

### 2.13 Construction of truncated *IRF8* core promoter vectors

*IRF8* promoter sequence was analyzed by the Alibaba2 online database for predicting the underlying TF-binding site. Based on the BDGP online software core promoter prediction, truncated vectors (Control, –300 to –1 bp; fragment 1, –500 to –1 bp; fragment 2, –1000 to –1 bp) were constructed separately for –1000 to –1 bp, where the CpG island was located, and then ligated to the pGL3-basic vector.

### 2.14 Construction of promoter recombinant plasmid and *M.Sss*I methylation treatment

Amplification fragment of 293 bp (–782 to –490 bp) in the *IRF8* promoter was amplified via PCR using primers with *Spe*I and *Nco*I restriction site at the 5′-end (**Table S7**). The recombinant plasmid was constructed with the product and a pCpGL-basic linearized vector. We also amplified fragments (–500 to 0 bp) that deleted or mutated the putative *IRF8* binding site. IRF8-WT contains the DNA fragment from –500 to 0 bp in the pGL3-basic. The mutant fragment of IRF8-Mut includes a sequence mutated from CCCGGCGGCC to TTTAATAATT. Moreover, the CCCGGCGGCC sequence was removed to generate IRF8-Del. Recombinant plasmids, IRF8-P1, and IRF8-P2, were methylated by *M.Sss*I, and the product was purified. Products were then transfected into IPEC-J2 cells using an unmethylated vector (Control) or pCpGL-basic. Relative fluorescence intensity was quantified to determine the role of hypermethylation on *IRF8* promoter transcriptional activities. IPEC-J2 was treated with 5’-Aza-2dC demethylases (Sigma-Aldrich).

### 2.15 Dual-luciferase reporter assay

Firefly luciferase reporter vectors (100 ng) along with IRF8 (WT, Mut, or Del), AP-2α-OE, and 2 ng of pRL-TK (calibrated as internal reference) were co-transfected into IPEC-J2 by Lipo2000. Relative fluorescence intensity was detected 48 h later by a Dual-Luciferase Reporter system. 100 μL passive lysis buffer was added into cells and incubated for 30 min with slight shaking, and 20 μL of cell lysates were used for determination. Data analyses were repeated thrice, each performed 6 times.

### 2.16 RNA-seq library construction and sequencing

IRF8-KO and wild-type IPEC-J2 were cultured and infected with PEDV of 0.1 MOI. After 48 h of incubation, PEDV-infected IRF8-KO (IRF8-KO+PEDV), Control (PEDV), and wild-type (mock) cells were harvested to perform RNA-seq. Total RNA was extracted and purified by the AMPure XP system (Beckman Coulter, California, USA). cDNA library was conducted and sequenced by Illumina HiSeq 2000 sequencer (BGI Tech, Hong Kong). The original data were processed for quality control by removing the adapters and low-quality reads. The Sscrofa11.1 genome was used to align reads by TopHat2 software (23). HTSeq software was used to calculate gene expression (24), and FPKM values were calculated. Finally, differential gene expression analysis was conducted by DESeq software (25). Adjusted *P* value < 0.05 and |log2-fold change| >2 was defined as a differentially expressed gene (DEG). GO and KEGG pathway enrichment in the differential gene set was conducted by Cluster Profiler software.

### 2.17 Measurement of the oxidative stress index

Control and IRF8-OE cells were seeded at 80% confluency for PEDV infection. After the infection for 48 h, the cells of Control, PEDV, IRF8-OE, and IRF8-OE+PEDV groups were centrifuged (4000 rpm for 10 min) to get the supernatant. Then the BCA Protein Assay Kit (CWBiotech, Beijing, China) was used to detect the concentration. Total antioxidant capacity (T-AOC) and the levels of adenosine triphosphate (ATP), nicotinamide adenine dinucleotide (NADH), catalase (CAT), superoxide dismutase (SOD), glutathione peroxidase (GSH-Px), and malondialdehyde (MDA) were determined following the detection kits (Jiancheng, Nanjing, China).

### 2.18 Statistical analysis

Statistical analyses were conducted by GraphPad Prism. Data are shown as the mean ± standard error or standard deviation and repeated at least thrice. Statistical analyses were performed by a two-tailed Student’s *t*-test. *P < 0.05, **P < 0.01.

## 3 Results

### 3.1 *IRF8* expression is strongly associated with PEDV infection

**Figure 1:**
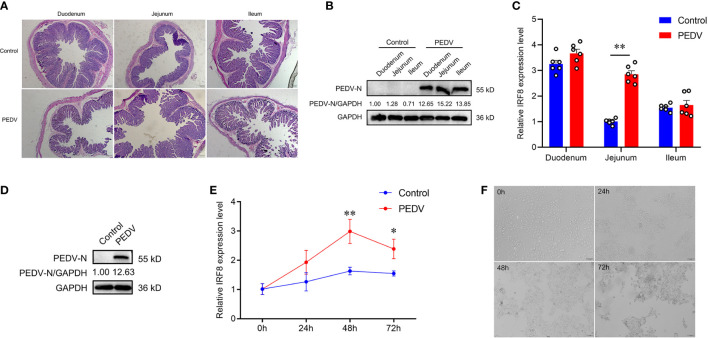
*IRF8* expression is strongly associated with PEDV infection. **(A)** HE staining of the intestinal tissues under the light microscope. **(B)** Protein expression levels of PEDV-N in the three intestinal segments of PEDV-infected and Control piglets. **(C)** *IRF8* mRNA expression level in the duodenum, jejunum, and ileum. **(D)** Protein expression levels of PEDV-N in the IPEC-J2 infected with PEDV. **(E)** *IRF8* mRNA expression in PEDV-infected IPEC-J2 after 24, 48, and 72 h. **(F)** Light microscopy observation of PEDV-infected IPEC-J2 after 24, 48, and 72 h. All images were taken at 4 × magnification. *P < 0.05, **P < 0.01.

PCR products of 6 diarrheal piglets revealed that the amplified fragment was PEDV strain (JSCZ1601), whereas no bands of TGEV, PDCoV, and PoRV were detected (**Figures S1A, B**). HE staining revealed that the intestinal villi of the mucosal epithelium in PEDV-infected piglets were broken and detached, and the height of the intestinal villi was reduced (**Figure 1A**). It was found that N protein was expressed in different intestinal segments of PEDV-infected piglets (**Figure 1B**). We also found that the *IRF8* mRNA expression in PEDV-infected jejunum was significantly higher than those in the Control (**Figure 1C**). N protein was also expressed in IPEC-J2 infected with PEDV (**Figure 1D**) and mRNA expression levels of *IRF8* were significantly upregulated 48 h PEDV post-infection (hpi) in IPEC-J2 (**Figure 1E**). Moreover, the morphology of cells infected with PEDV for 24, 48, and 72 h was observed by microscopy. As shown in **Figure 1F**, the cells changed from a flat spindle to a spherical shape, with some cells showing shrinkage. The cells showed complete lesions at 72 hpi and cellular complete morphology was lost.

### 3.2 Downregulation of *IRF8* expression facilitates PEDV infection

**Figure 2:**
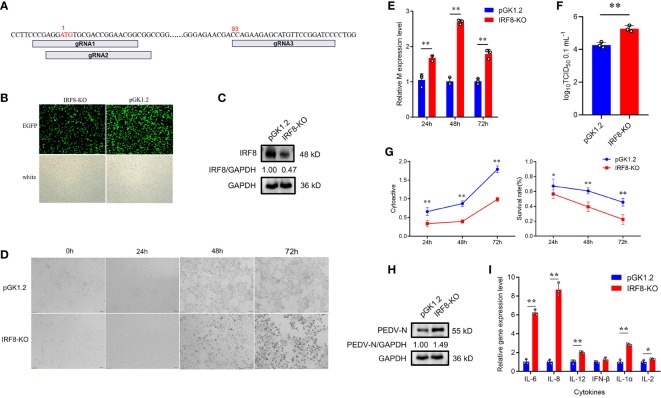
Downregulation of *IRF8* expression facilitates PEDV infection. **(A)** Locations of 3 Single guide RNAs (sgRNAs) used for *IRF8* depletion. **(B)** Fluorescence field and white light images of IPEC-J2 cells transfected with the *IRF8* knockout plasmid. **(C)** Western blotting of IRF8 protein expression levels after *IRF8* knockout. **(D)** Cytologic observation of pGK1.2 and IRF8-KO cells after PEDV infection for 24, 48, and 72 h. **(E)** Expression levels of *M* gene in pGK1.2 and IRF8-KO groups after PEDV infection for 24, 48, and 72 h. **(F)** PEDV virulence in pGK1.2 and IRF8-KO cells after PEDV infection was detected using TCID_50_. **(G)** Activities of pGK1.2 and IRF8-KO cells via cell counting kit (CCK)-8 assay. **(H)** Protein expression levels of PEDV-N in pGK1.2 and IRF8-KO cells after PEDV infection. **(I)** Expression levels of inflammatory genes in pGK1.2 and IRF8-KO cells. All images were taken at 4 × magnification. *P < 0.05, **P < 0.01.

To understand the relationship between *IRF8* and PEDV infection, we constructed *IRF8* knockout cell line. **Figure 2A** shows the relative positions of gRNAs in the genome sequences. More than 80% of green fluorescence was visualized by fluorescence microscope, indicating that the knockout vector was transfected into IPEC-J2 cells (**Figure 2B**). The PCR amplification result showed a significant reduction in sequence length (**Figure S2**). Furthermore, IRF8 protein was barely expressed in IRF8-knockout cells via western blotting (**Figure 2C**). The morphological difference was observed by microscope upon PEDV infection (**Figure 2D**). IRF8-KO cells showed more severe lesions infected with PEDV compared to that of the pGK1.2 vector infected with PEDV, especially at 72 hpi, and almost all cells shrank and fused with partial necrosis. In addition, RT-qPCR revealed that *IRF8* knockout significantly upregulated the mRNA level of the *M* gene (**Figure 2E**). TCID_50_ assay was conducted to detect the viral titer in IRF8-KO and Control cells upon PEDV infection, and the result demonstrated that PEDV replication increased significantly after *IRF8* knockout (**Figure 2F**). Moreover, cell proliferation and survival rate were decreased in the IRF8-KO group after PEDV infection, suggesting that *IRF8* depletion inhibits cell viability (**Figure 2G**). Knockout of *IRF8* also increased PEDV N protein levels (**Figure 2H**). To explore whether PEDV causes inflammation, the mRNA level of key inflammatory cytokines was measured. RT-qPCR showed that cytokine expression was significantly upregulated after *IRF8* knockout (**Figure 2I**).

**Figure 3:**
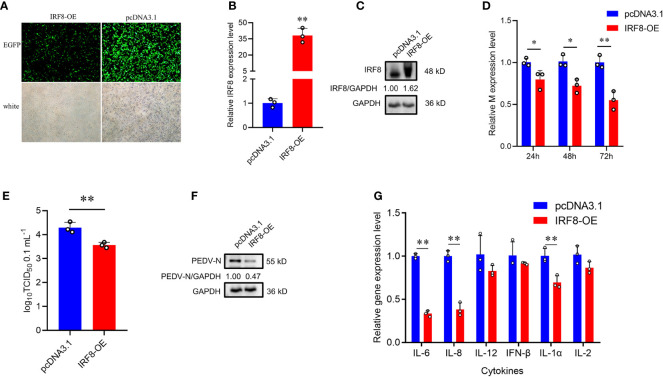
Up-regulation of *IRF8* suppresses PEDV infection. **(A)** Fluorescence field and white light images of IPEC-J2 transfected with IRF8 overexpression plasmid. **(B)** The mRNA expression level of IRF8 after transfection of the overexpressed plasmid into IPEC-J2. **(C)** Western blot results of IRF8 protein expression after IRF8 overexpression. **(D)** M gene expression in pcDNA3.1 and IRF8-OE groups after PEDV infection for 24 h, 48 h, and 72 h. **(E)** PEDV virulence of pcDNA3.1 and IRF8-OE cells after PEDV infection determined by TCID_50_. **(F)** The protein expression level of PEDV-N in pcDNA3.1 and IRF8-OE cells after PEDV infection. **(G)** Expression levels of inflammatory genes in pcDNA3.1 and IRF8-OE cells. *P < 0.05, **P < 0.01.

To further demonstrate that *IRF8* affects PEDV invasion, an *IRF8* overexpression vector was constructed. As shown in **Figure 3A**, IRF8-OE, and pcDNA3.1-EGFP vectors were transfected into cells. The *IRF8* mRNA and protein level was significantly upregulated compared to the pcDNA3.1-EGFP vector (**Figures 3B, C**). In addition, *M* gene expression was significantly downregulated in the IRF8-OE group than in Control cells (**Figure 3D**). TCID_50_ assay revealed that the PEDV replication was inhibited after *IRF8* overexpression along with the N protein expression (**Figures 3E, F**). Cytokine IL-6, IL-8, and IL-1α expression were significantly downregulated in IRF8-OE cells infected with PEDV (**Figure 3G**).

### 3.3 *IRF8* expression is negatively correlated with the promoter methylation status

**Figure 4:**
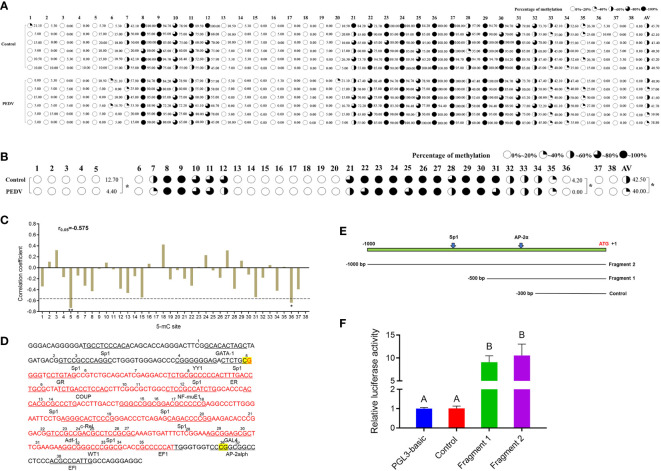
*IRF8* expression is negatively correlated with the promoter methylation status. **(A)** Methylation status of CpG island site in *IRF8* gene promoter. **(B)** Methylation level differences at each locus between PEDV and Control groups. **(C)** Correlation between mRNA expression and *IRF8* methylation. **(D)** Transcription factor binding prediction of CG sites on *IRF8* CpG island. **(E)** Schematic diagram of truncated fragment sequences synthesized according to the location of key CG sites. **(F)** Dual-luciferase results after transfection of the truncated fragment recombinant plasmid into IPEC-J2 cells. Different capital letters represent *P* < 0.01. *P < 0.05.

To determine whether DNA methylation regulates the mRNA expression of *IRF8*, a CpG island (–398 to –106 bp) was predicted in the *IRF8* promoter (**Figure S3**). *IRF8* methylation profiles in PEDV-infected piglets and Control groups were determined using BSP. As shown in **Figure 4A**, 38 CpG sites with different methylation statuses were observed in the amplification fragments. **Figure 4B** depicts the significant difference in mC-5 loci, with methylation status of 12.7 and 4.4% in the Control and PEDV groups, respectively. Moreover, the methylation status of 4.2% in the Control group at the mC-36 loci showed a higher degree compared to the PEDV-infected group. Importantly, the average methylation status in the Control group was also higher than the PEDV-infected group. Pearson correlation revealed that the *IRF8* expression was negatively correlated with CpG island methylation statuses (**Figure 4C**, r = –0.575). Transcriptional factor prediction revealed that the potential transcription factors in the promoters at mC-5 and mC-36 were Sp1 and AP-2α, respectively (**Figure 4D**). Based on the sequences of the transcription factor-binding site where Sp1 and AP-2α are located, we constructed 3 recombinant vectors (**Figure 4E**). The dual-luciferase assay revealed that the activity of fragment 1 was significantly higher than the Control group (**Figure 4F**). The data indicated that the core promoter of *IRF8* is located between –301 and –500 bp, where AP-2α is located.

### 3.4 Hypermethylation status suppresses AP-2α-mediated IRF8 transcription

**Figure 5:**
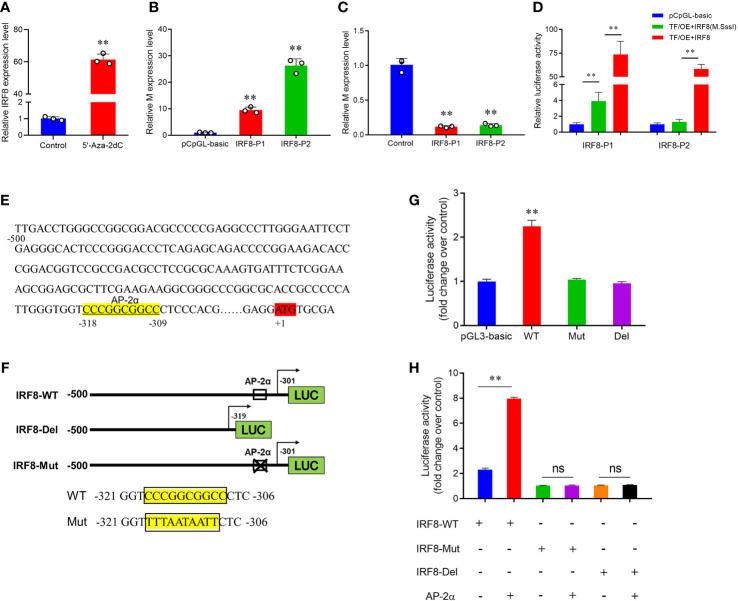
Hypermethylation suppresses AP-2α-mediated IRF8 transcription. **(A)** Relative expression levels of IRF8 before and after demethylase treatment. **(B)** Relative expression levels of *M* gene after transfection of methylase-treated recombinant plasmids into PEDV-infected IPEC-J2. **(C)** Relative *M* gene expression under demethylase treatment after transfection of the recombinant plasmids into PEDV-infected IPEC-J2 cells. **(D)** Dual-luciferase results after co-transfer of Sp1 and AP-2α overexpression plasmids with IRF8 recombinant plasmid before and after methylase treatment, respectively. **(E)** Schematic representation of the porcine *IRF8* promoter. AP-2αbinding sites are highlighted in yellow. ATG region is highlighted in red. **(F)** Construction of IRF8-WT, IRF8-Del (with or without the AP-2α binding site), and the IRF8-Mut recombinant plasmids (mutated AP-2α binding site). **(G)** Detection of dual-luciferase activity after transfection of IRF8-WT, IRF8-Del, and IRF8-Mut recombinant plasmids into cells, respectively. **(H)** Dual-luciferase activity assay before and after co-transfection of IRF8-WT, IRF8-Del, and IRF8-Mut recombinant plasmids with AP-2a overexpression vector, respectively. ns, not significant, **P < 0.01.

To verify whether DNA methylation affects *IRF8* expression, the effect of promoter methylation on *IRF8* activity was assessed via a dual-luciferase reporter assay. RT-qPCR revealed that *IRF8* expression was significantly upregulated after 5’-Aza-2dC treatment (**Figure 5A**). Moreover, PEDV invasion was markedly activated by methylase treatment, and *M* gene expression in the recombinant plasmid containing AP-2α binding site (IRF8-P2) was significantly higher than that in the recombinant plasmid containing Sp1 binding domain (IRF8-P1; **Figure 5B**). In addition, *M* gene expression was significantly downregulated in both IRF8-P1 and IRF8-P2 groups after 5’-Aza-2dC treatment (**Figure 5C**). To further determine the effect of TFs on *IRF8* activity, overexpression vectors of Sp1 and AP-2α were co-transfected into IPEC-J2 cells with core promoter recombinant plasmid after methylase treatment. **Figure 5D** shows that transcriptional activity was activated by transcription factors co-transfected with a methylated reporter compared to those transfected with an unmethylated vector. Moreover, AP-2α plays a critical role in *IRF8* transcription activation.

Whether AP-2α directly regulates *IRF8* expression remains unclear. We hypothesize that AP-2α binds to the IRF8 promoter and acts as a transcriptional activator that directly regulates its expression. Therefore, we constructed vectors containing *IRF8* promoter with or without AP-2α binding sites (**Figures 5E, F**). Dual-luciferase assay revealed that AP-2α activates the *IRF8* activity, rather than the truncated vector without AP-2α binding sites (**Figure 5G**). In addition, we further demonstrated that AP-2α failed to regulate the vector upon *IRF8* binding sites mutation (**Figure 5H**).

### 3.5 *IRF8* knockdown increases PEDV-induced oxidative phosphorylation and apoptosis abnormality

**Figure 6:**
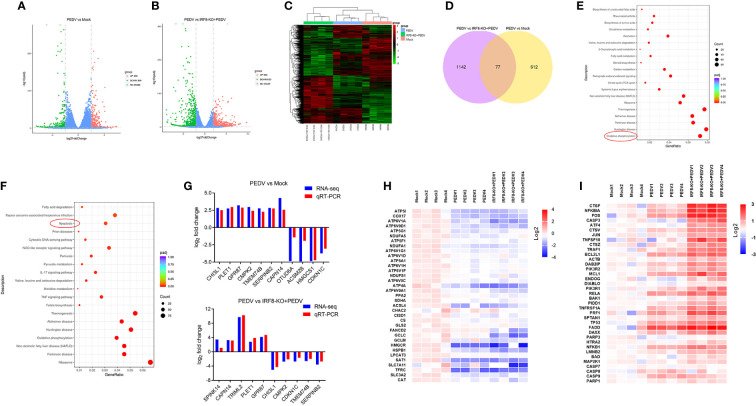
*IRF8* knockdown increases PEDV-induced oxidative phosphorylation and apoptosis abnormality. **(A)** Volcano plot of DEGs between the Mock and PEDV-infected cells. **(B)** Volcano plot of DEGs between the PEDV and IRF8-KO + PEDV cells. **(C)** Cluster analysis of DEGs among the 3 groups. **(D)** Venn plot of screened DEGs among 3 groups. **(E)** KEGG enrichment analysis of DEGs between the Mock and PEDV groups. **(F)** KEGG enrichment analysis of DEGs between the PEDV and IRF8-KO + PEDV groups. **(G)** Gene expression alignment of RNA-seq with RT-qPCR results in Mock, PEDV, and IRF8-KO + PEDV groups. **(H)** Expression clustering heatmap of oxidative phosphorylation pathway genes in the 3 groups. **(I)** Expression clustering heatmap of apoptosis pathway genes in the 3 groups.

Transcripts of mock, PEDV, and IRF8-KO + PEDV cells were obtained via RNA-seq in IPEC-J2 cell lines. We identified 689 DEGs in mock and PEDV cells, of which 300 were upregulated and 389 were downregulated in the PEDV group (**Figure 6A**, **Table S8**). Moreover, 1219 DEGs were determined between the PEDV and IRF8-KO + PEDV cells, among which *IRF8* knockout caused 586 genes downregulation and 633 genes upregulation compared to those in the PEDV group (**Figure 6B**, **Table S9**). Heatmaps of clustered genes with significantly altered PEDV expression showed similarities in expression change caused by *IRF8* knockout (**Figure 6C**). Venn diagram shows that 77 altered gene expression in the PEDV group was suppressed by *IRF8* knockout (**Figure 6D**, **Table S10**). Enrichment analysis by KEGG demonstrated that the oxidative phosphorylation pathway was highly enriched in the mock and PEDV groups, and the apoptosis pathway was mainly enriched in the PEDV and IRF8-KO + PEDV groups (**Figures 6E, F**). Based on the fold difference in gene expression and biological function, 13 genes (*CHI3L1*, *PLET1*, *GPR87*, *CMPK2*, *CDKN1C*, *TMEM74B*, *SERPINB2*, *SPINK14*, *CAPN14*, *TRIML2*, *OTUD6A*, *ACSM2B*, and *HMGCS1*) were selected for RT-qPCR assay, and the result was in line with RNA-seq (**Figure 6G**). Heat maps of gene expression in oxidative phosphorylation and apoptosis pathways were drawn according to the RNA-seq. Oxidative phosphorylation genes were significantly downregulated upon PEDV infection, whereas *IRF8* knockdown exacerbated the PEDV-induced decrease in gene expression levels (**Figure 6H**). However, most apoptosis-related gene expression was significantly upregulated after PEDV infection, and *IRF8* knockdown exacerbated the elevated gene expression caused by PEDV (**Figure 6I**).

### 3.6 *IRF8* expression is associated with PEDV-induced oxidative stress

**Figure 7:**
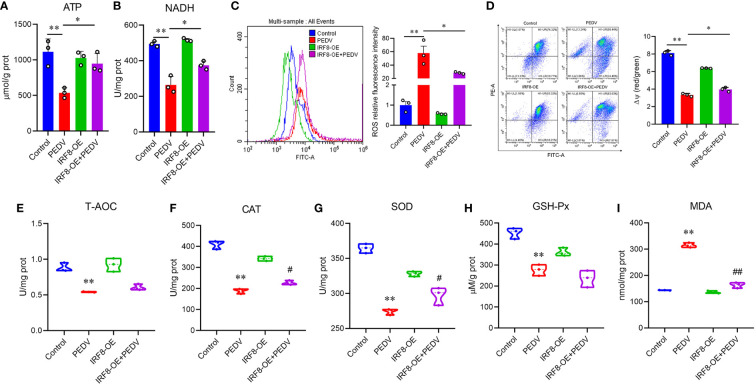
*IRF8* expression is associated with PEDV-induced oxidative stress. **(A, B)** Determination of ATP and NADH levels in 4 groups. **(C)** Fluorescence intensity of ROS in the 4 groups. **(D)** Mitochondrial membrane potential levels in the 4 groups of cells. Ratio of red over green fluorescence intensity in flow cytometry is indicated by ΔΨ. **(E–I)** Detection of the T-AOC, CAT, SOD, GSH-Px, and MDA levels in different groups. *Compared to the Control group; ^#^compared to the PEDV group.

As endogenous energy sources, ATP and NADH levels were significantly decreased in cells infected with PEDV, which were rescued by the upregulation of *IRF8* levels (**Figures 7A, B**). To clarify the effect of *IRF8* in oxidative stress caused by PEDV, ROS levels and MMP of the cells were measured. PEDV infection increased the cellular ROS levels, which were reduced by *IRF8* overexpression (**Figure 7C**). In contrast, *IRF8* overexpression alleviated the decrease in MMP induced by PEDV (**Figure 7D**). Concomitant with the decrease in T-AOC in PEDV-infected cells (**Figure 7E**), PEDV infection significantly decreased CAT, SOD, and GSH-Px levels in cells, whereas *IRF8* overexpression alleviated the decrease in CAT and SOD levels induced by PEDV infection (**Figures 7F–H**). Importantly, *IRF8* overexpression significantly diminished the PEDV-induced increase in lipid peroxide and MDA levels induced by PEDV (**Figure 7I**).

### 3.7 *IRF8* expression is essential for PEDV-induced apoptosis

**Figure 8:**
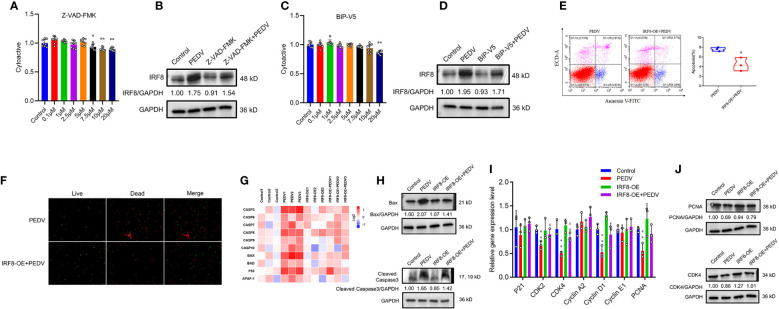
*IRF8* is essential for PEDV-induced apoptosis. **(A, C)** Effects of gradient concentration inhibitors (Z-VAD-FMK and BIP-V5) on cell viability. **(B, D)** Protein expression levels of IRF8 in the 4 groups. **(E)** Apoptosis of cells evaluated via flow cytometry with annexin V staining. **(F)** Cell viability is determined using live/dead reagent staining. **(G)** Heatmap of apoptosis gene mRNA expression (Log_2_) normalized to Control. **(H)** Protein expression of apoptotic protein in 4 groups. **(I)** Relative mRNA expression of cell cycle genes. **(J)** Expression levels of proliferation proteins in 4 groups. *Compared to the Control group; #compared to the PEDV group.

Pan-caspase inhibitors, Z-VAD-FMK, and Bax inhibitor, BIP-V5, were selected to rescue cell apoptosis. IPEC-J2 were treated with inhibitors at gradient concentrations (0.1, 1, 2.5, 5, 7.5, 10, and 20 μM). CCK-8 assay revealed that cell viability decreased with 7.5 μM Z-VAD-FMK and 20 μM BIP-V5 (**Figures 8A, C**). Therefore, concentrations of 5 μM Z-VAD-FMK and 10 μM BIP-V5 were suitable for preventing the apoptosis of IPEC-J2 cells. To verify whether *IRF8* affects apoptosis, its protein expression was analyzed. **Figure 8B** revealed that PEDV increased the expression of *IRF8* that was suppressed by Z-VAD-FMK. As expected, elevated expression of IRF8 was observed in the PEDV group, which was recovered by BIP-V5 treatment (**Figure 8D**). Both inhibitors and clustering heatmap results jointly proved that *IRF8* played a repressive role in PEDV-induced apoptosis. Flow cytometry revealed that *IRF8* overexpression markedly decreased the ratio of apoptotic cells compared to that by PEDV alone (**Figure 8E**). In addition, fluorescence staining further confirmed that high expression of *IRF8* improves the cell viability compared with the PEDV group (**Figure 8F**). Furthermore, the heatmap revealed that *IRF8* overexpression strongly inhibited the PEDV-induced increase in apoptotic gene expression (**Figure 8G**). Immunoblotting revealed that IRF8 overexpression efficiently downregulated the cleaved caspase 3 and Bax protein expression compared to the PEDV group (**Figure 8H**). In contrast, RT-qPCR results revealed that most cell cycle-related gene expression was significantly downregulated by PEDV, and upregulation of *IRF8* expression restored the cell cycle (**Figure 8I**). Compared to the PEDV group, *IRF8* overexpression resulted in strong upregulation of PCNA and CDK4 levels (**Figure 8J**).

## 4 Discussion

PED is a highly contagious enterovirus disease induced by PEDV and has a high fatality rate in neonatal piglets (26). As the most abundant envelope component of PEDV, the M protein perform a vital effect in virus assembly (27). In this study, *M* gene amplification and intestinal morphology observations showed that PEDV bands could be amplified in piglets infected with PEDV, accompanied by symptoms, such as intestinal villous atrophy, epithelial cell necrosis, and shedding. Combined with the fact that no other typical diarrheal pathogens (TGEV and PoRV) were detected in these piglets (28), PEDV was determined to be the only pathogen of infection.

As an immunoregulatory factor, *IRF8* is involved in the regulation of various immune processes (29). Although there have been several studies on *IRF8* antiviral activity (13, 30, 31), its contribution to resistance to PEDV, one of the most harmful viruses to piglets, remains unclear. PEDV is particularly harmful to neonatal piglets (32). This study detected *IRF8* expression in duodenum, jejunum, and ileum of a 7-day-old piglet and found that PEDV infection can activate *IRF8* gene expression. The main PEDV target is the porcine intestinal epithelial cell (5). Therefore, a PEDV-infected cell injury model was constructed, and the result revealed that PEDV infection induced increased *IRF8* expression. With the property of conservation, the M protein is an important structural protein that viruses stimulate the host to produce immune protection (33). Therefore, the virulence of PEDV can be determined by detecting the expression of the M protein. The degree of cytopathic deepening is accompanied by the enhancement of PEDV virus copy and titer with the *IRF8* knockout IPEC-J2 cell model. *IRF8* is a key factor in the infection process of PEDV. *IRF8* overexpression may inhibit viral reproduction and PEDV replication. Notably, *IRF8* plays a vital effect in cell growth and differentiation (34). Herein, we addressed the fact that PEDV infection reduced the proliferative activity of IPEC-J2 cells, blocked the cell cycle, and induced apoptosis (35). This provides the basis for supporting *IRF8*-regulated cell cycle and cell arrest. We demonstrated that cell activity and viability were significantly reduced and cells were arrested after *IRF8* knockout. Based on these findings that *IRF8* depletion hinders cell proliferation during PEDV invasion, we speculated that *IRF8* may rebuild the epithelial cell barrier by promoting cell proliferation. The inflammatory response triggered by a viral infection can induce host morbidity. *IRF8* has been extensively studied in antiviral activity and modulation of inflammatory responses (36, 37). Expression levels of proteins involved in the inflammatory response increase upon PEDV infection (38). Consistent with our results, the cytokine expression significantly increased after *IRF8* knockdown and high expression of *IRF8* resisted virus invasion. Taken together, *IRF8* deletion during PEDV infection further enhances the viral infection process and produces a severe inflammatory response in the host.

DNA methylation blocks transcriptional activation of gene promoters in cells by inhibiting specific TFs to bind to DNA, which is often associated with disease development (39, 40). Previous studies demonstrated that the *IRF8* may be epigenetically regulated (41). However, whether *IRF8* expression is also modulated by DNA methylation during PEDV infection remains unknown. We speculate that PEDV alters the methylation level of the *IRF8* promoter, and hypomethylation at mC-5 and mC-36 sites located in the core promoter region is associated with increased *IRF8* expression. Hypomethylation of promoter DNA is often regulated by transcription factors (42). Interestingly, transcription factor AP-2α was more sensitive to *IRF8*-mediated transcription than Sp1. We also speculated that Sp1, AP-2α, and IRF8 play synergistic roles via changes in gene expression involved in PEDV infection. Sp1 and AP-2α may regulate the *IRF8* expression to resist PEDV invasion. However, the co-transformation experiments with IRF8 promoter methylase treatment and transcription factor overexpression plasmids provided further evidence for the specifically targeted activation of IRF8 by AP-2α, and we proved that AP-2α negatively regulates PEDV replication by activating the expression of *IRF8*. In addition, AP-2α participates in the antiviral gene regulation process via post-translational modification (43, 44). This may be another mechanism for AP-2α to modify the expression of *IRF8* under PEDV infection, which requires further investigation.

Further functional enrichment analysis demonstrated that the oxidative phosphorylation pathway was significantly altered upon PEDV infection, whereas the apoptotic pathway was closely related to *IRF8* knockout. Cellular energy generation is primarily through oxidative phosphorylation (45). Deletion of *IRF8* blocks energy production caused by PEDV, which is manifested as oxidative phosphorylation in cells caused by viral stimulation and increased energy consumption. This may be associated with the energy metabolism modification upon PEDV infection, which meets the energy requirements for virus assembly (46). Notably, the mitochondria also generate ROS during oxidative phosphorylation. Upregulated oxidative phosphorylation induced by PEDV affects reversible cysteine redox modification by generating high levels of ROS and plays a key role in lipid molecule-induced oxygen stress (47). Inhibition of ROS generation and restoration of MMP via the activation of *IRF8* suggest that *IRF8* may be an important metabolic regulator during oxidative stress. The reduction in antioxidant enzyme activity and intracellular lipid peroxidation further indicates that high expression of *IRF8* is beneficial for maintaining the redox homeostasis of the organism during pathogen infection (48). Interestingly, PEDV induces apoptosis in Vero through ROS/p53, suggesting that activation of oxidative phosphorylation is associated with apoptosis (49). IRF8 is a key regulator of apoptosis inhibitory protein, which can defend against pathogenic infection (50). In our study, treatment with Z-VAD-FMK and BIP-V5 alleviated the PEDV-induced increase in IRF8 protein expression levels, implying that IRF8 is involved in PEDV-induced apoptosis. Decreased expression levels of key apoptotic proteins further illustrate that high expression of *IRF8* is beneficial for viral infection-activated apoptosis and changes in biological processes, such as the cell cycle (35).

In summary, our study revealed that AP-2α positively regulates *IRF8* via promoter methylation and activation of *IRF8* resists PEDV replication by inhibiting oxidative stress and apoptosis. Our finding provides valuable insight into the *IRF8* in PEDV replication and lays a foundation for developing therapeutic strategies for PEDV-associated diseases.

## Data availability statement

The data presented in the study are deposited in the NCBI Sequence Read Archive repository, and the accession numbers are SRR25409507/ SRR25409506/ SRR25409505/ SRR25409504/ SRR25409503/ SRR25409502/ SRR25409501/ SRR25409500/ SRR25409499/ SRR25409498/ SRR25409497/ SRR25409496/ PRJNA998179.

## Ethics statement

All animal experiments were approved by the Institutional Animal Care and Use Committee (IACUC) of the Yangzhou University Animal Experiments Ethics Committee (permit number: SYXK (Su) IACUC 2012-0029). All experiments were performed following the relevant guideline.

## Author contributions

QZ and HQ conducted the majority of the experiments; XZ, HW, and SW took part in some experiments; WB and ZY conceived this study, QZ, HQ, and WB took part in its design and wrote the manuscript. Every author consented to the final version.
