# Supplementary material for: Hypomethylated interferon regulatory factor 8 recruits activating protein-2α to attenuate porcine epidemic diarrhea virus infection in porcine jejunum
Source: Front Immunol. 2023 Aug 1;14:1187144. doi: 10.3389/fimmu.2023.1187144 (PMC10427914; doi:10.3389/fimmu.2023.1187144)
Supplement: Supplementary file 1 [file Presentation_1.pdf]

Supplementary Material

Hypomethylated Interferon Regulatory Factor 8 Recruits Activating Protein-2α to Attenuate Porcine Epidemic Diarrhea Virus Infection in Porcine Jejunum

Qiufang Zong[1†], Huan Qu[1†], Xianrui Zheng[2], Haifei Wang[1], Shenglong Wu[1], Zongjun Yin[2*], Wenbin Bao[1*]

*** Correspondence:** Wenbin Bao: wbbao@yzu.edu.cn; Zongjun Yin: yinzongjun@ahau.edu.cn;

1 Supplementary Figures

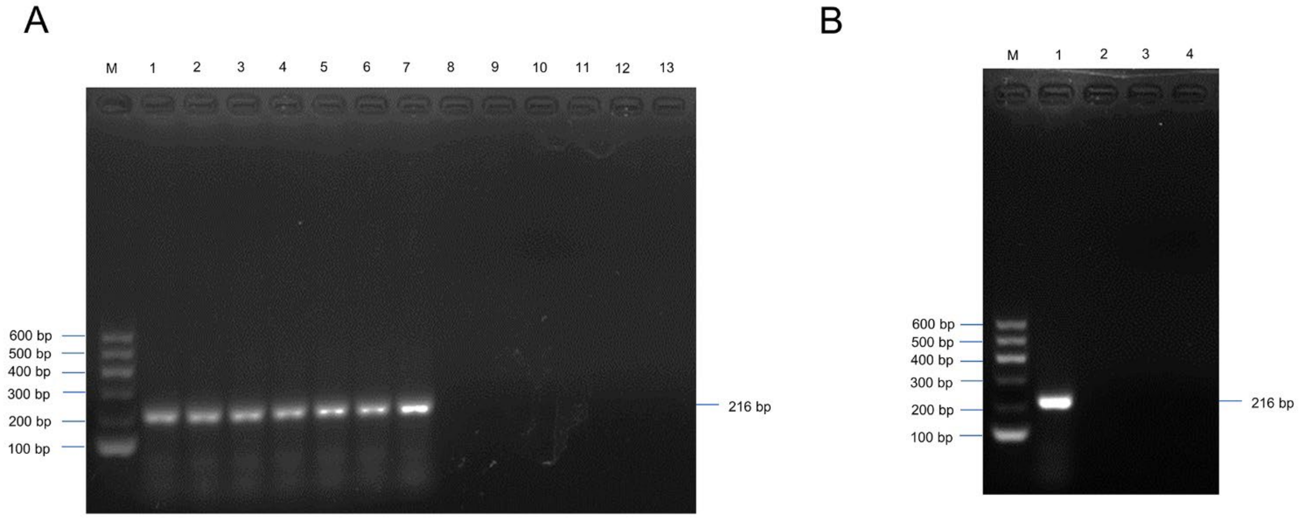

Supplementary Figure 1. Pathogen identification of diarrhea piglets. (A) Agarose gel results of M gene amplification of PEDV classical strains CV777. M, DNA marker I; 1-6, diarrheal piglets; 7, positive control; 8-13, control piglets. **(B)** Etiology identification of multiple porcine coronaviruses. M, DNA marker I; 1, PEDV; 2, TGEV; 3, PDCoV; 4, PoRV.

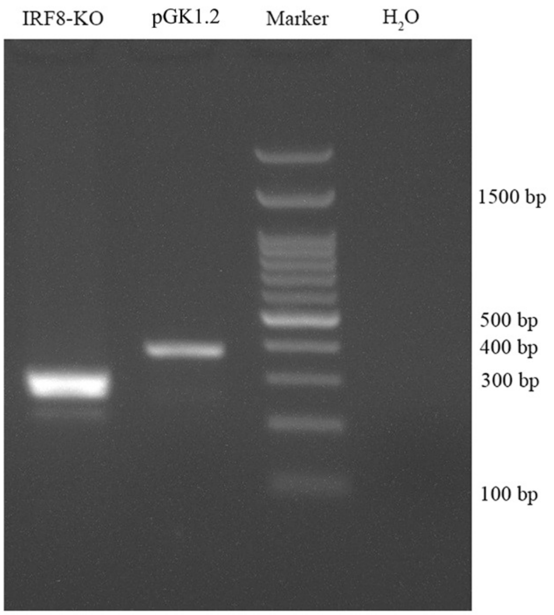

Supplementary Figure 2. Nucleic acid gel detection of IRF8 gene by PCR amplification.

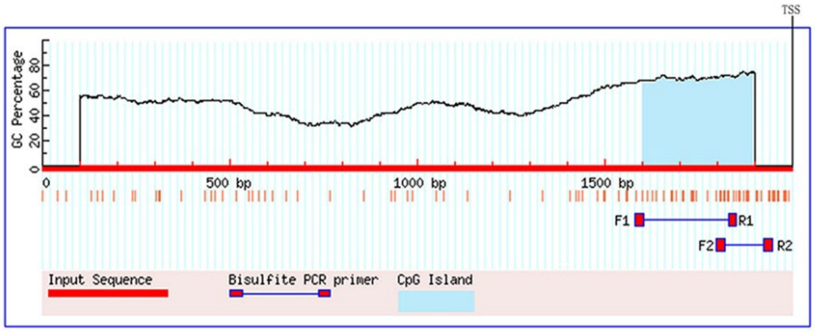

Supplementary Figure 3. CpG island prediction of IRF8 gene promoter region.

Supplementary Tables

Table S1 Primers for pathogen identification

Genes	Accession number	Primer sequence (5'-3')	Length (bp)
PEDV (M)	AF353511.1	F: AGGTCTGCATTCCAGTGCTT<br>R: GGACATAGAAAGCCCAACCA	216
TGEV(S)	FJ755618	F: CCAAACAGCCGTTATTAGTTA<br>R: AGTGACACCACCCGTTGT	218

PDCoV(N)	JQ065043	F: ATGGCTACTGGCTGCGTTAC<br>R: GCGTTTCCTGGGCTGATT	383
PoRV(VP6)	FJ807867	F: CAAACGGGAGGAATAGGAA<br>R: CACTCTTGGGAAACTGAACC	527

Table S2 SgRNA oligo sequences

Name	sgRNA sequence (5'-3')	Length (bp)
sgRNA1	CCGTTCCGGTCGCACATCCTCGG	23
sgRNA2	AGGATGTGCGACCGGAACGGCGG	23
sgRNA3	GGATCCGGAACATGCTCTTCTGG	23

Table S3 Primers for identifying knockout efficiency of IRF8

Name	Primer sequence (5'-3')	Length (bp)	Temperature (℃)
IRF8-sg	TGAAGCGGCACCTCTGTCCT<br>AAAGAAACCGGAGTCCTGTG	370	60

Table S4 Real-time PCR primer sequences

Genes	Accession number	Primer sequences (5'-3')	Length (bp)
IRF8	NM_001252427.2	F: GACGAGGCTACGCTGTGCTTT<br>R: CTCTGTGGCTTCGCTCATGCA	169
IL-6	NM_001252429.1	F: ATCTGGGTTCAATCAGGAGACCT<br>R: ATTTGTGGTGGGGTTAGGGG	208
IL-8	NM_213867.1	F: CCACACCTTTCCACCCCAAA<br>R: TTGTTGCTTCTCAGTTCTCTTCA	179
IL-12	NM_213993.1	F: CAGGCCCAGGAATGTTCAAA	166

		R: CGTGGCTAGTTCAAGTGGTAAG	
IFN-β	JN391525.1	F: GCTAACAAGTGCATCCTCCAAA<br>R: CCAGGAGCTTCTGACATGCCA	124
IL-1α	NM_214029.1	F: ACCTGGATGAGGCAGTGAAAT<br>R: ATGGGCGGCTGATTTGAAGT	236
IL-2	XM_003358973.3	F: GTTGCATTGCACTAACCCTT<br>R: TGGCTCCAGTTGTTTCTTTG	86
P21	NM_001291549.1	F: GACCAGCATGACAGATTTCTACC<br>R: GGCTTCCAGCAGCCTAGTTA	81
CDK2	XM_003481615.1	F: AAACAAGTTGACGGGAGA<br>R: GTGAGAATGGCAGAAAGC	297
CDK4	NM_001123097.1	F: GCATCCCAATGTTGTCCG<br>R: GGGTGCCTTGTCCAGATA	126
Cyclin A2	NM_001177926.1	F: GCTATGCTGTTGGCCTCAAAA<br>R: AGACTGCTGGTGCAGAAAGT	195
Cyclin D1	XM_021082686.1	F: GCGAGGAACAGAAGTGCG<br>R: TGGAGTTGTCGGTGTAGATGC	192
Cyclin E1	XM_005653265.2	F: CTGCCGCGGAGACGG<br>R: TTGTCCCAAGGCTGATTGCC	241
PCNA	NM_001291925.1	F: TGTAGCCGCGTCGTTGTGATTC<br>R: CGCTTCCAGCACCTTCTTCAGG	83
BCL2	XM_021099593.1	F: CAGAGGGGCTACGAGTGGGATG<br>R: CCGGGCTGGGAGGAGAAGATG	89
BAX	XM_003127290.5	F: ATCGGCTGCTGGGCTGGATC<br>R: ATGGTGAGCGAGGCGGTGAG	124
BAD	XM_021082883.1	F: CCGAGGAGGATGAAGGGACTGAG<br>R: AGGAACCCTGGAACTCGTCACTC	138

P53	NM_213824.3	F: GCCCATCCTCACCATCATCACAC<br>R: GCACAAACACGCACCTCAAAGC	81
APAF-1	XM_021093024.1	F: TACCCTGTTGGCGACTGGAGATG<br>R: ACTGGAGCACACGAATGAAGAAGC	87
CASP3	NM_214131.1	F: TGTGGGATTGAGACGGACAGTGG<br>R: GCCAGGAATAGTAACCAGGTGCTG	112
CASP6	XM_013989349.2	F: TCACGGCGAAGGCAATCACATC<br>R: TTTCCAACCAGGCTCTGACACTTG	102
CASP7	XM_021073322.1	F: AGATGCGGAGGCTCTTTTCAAGTG<br>R: AAGCAGGCTGAATTTCGGTGGTC	135
CASP8	NM_001031779.2	F: GAAAAGCAAGCCTCGGGGATACTG<br>R: GAGCCGCTGCATCCAAGTCTG	125
CASP9	XM_013998997.2	F: TGAAGACAGGACCGCCGACAG<br>R: ACTAGGTGTGGGCAGACTGGAC	109
CASP10	NM_001161640.1	F: TTTCGGCATGTGAAGGAAGGTAGC<br>R: ATTGTTGACGGCGGTGAGAATGG	111
CHI3L1	NM_001001540.1	F: ACACTCAAGAACAGGAACCCC<br>R: TGAGATGCCGCTTGTCTCTC	208
PLET1	NM_213744.1	F: CCGTCGAGCTACAAGTCTTC<br>R: TGTGTGGTGTGGGTTGTGAT	169
GPR87	XM_003132489.3	F: GCCAGCTACAGCAATGAAAGAAAT<br>R: GTGACGGACTGTAGGCACAA	109
CMPK2	XM_021087907.1	F: CCGTGACCCAGTCAGTTTCA<br>R: GCTGTGCCAGTACCTGTCTA	206
CDKN1C	XM_021082622.1	F: CTGCGATGAGAGCCTCG<br>R: CCTTGGGACCAGTGTACTCC	224
TMEM74B	XM_013985206.2	F: CCCAGCACAGCTGCCAAG	167

		R: CAGGCATTCTCCACACCCTC	
SERPINB2	XM_003121697.4	F: CCTCAGAGAATAACCAGATTGAAAC<br>R: TCACTGGGATGCCTTCATGG	240
SPINK14	XM_005661791.2	F: GCTCTTCCTTTCTGAGGCAAC<br>R: GCGGAGGATTGGGAAGGATT	100
CAPN14	XM_021088409.1	F: TTGGGAAGTGGATTCCGGTG<br>R: AGGAGCCAGAGAGCTTAGCA	141
TRIML2	XM_021078065.1	F: TGAGGTGAACTTCCAGAGCC<br>R: GTAGTGACTCGCTCCTTTCCA	299
OTUD6A	XM_003135157.5	F: AGCAGAAGCACCAGCAAGA<br>R: CCACGGTCACCGAGAACAC	339
ACSM2B	XM_003124561.6	F: GCTTCTTCTCTGGCTATGT<br>R: AAACGCCTTCACTACCTCT	263
HMGCS1	XM_021076557.1	F: TTATGGTTCTGGTTTGGC<br>R: AAGTGCTCTCATTTCGGG	308
GAPDH	AF017079.1	F: ACATCATCCCTGCTTCTACTGG<br>R: CTCGGACGCCTGCTTCAC	187

Table S5 Antibodies used

**Antibody**	**Vendor**	**Catalog number**	**Dilution**
IRF8	Lifespan bioscience	ls-c31015	1:1000
PEDV-N	Medgene	SD-1-5	1:500
Cleaved Caspase3	Cell signaling	Asp175	1:1000
Bax	HuaBio	ET1603-34	1:1000
PCNA	Proteintech	10205-2-AP	1:2000

CDK4	Proteintech	11026-1-AP	1:1000
GAPDH	Proteintech	10494-1-AP	1:5000
anti-Mouse IgG	HuaBio	HA1006	1:10000
anti-Rabbit IgG	HuaBio	HA1001	1:10000

Table S6 BSP-PCR primers

Name	Primer sequences (5'-3')	Length (bp)	Temperature (℃)
IRF8-1	F: GGGATAGGGGGATGTTTTTATATA<br>R: ATACCCTCAAAAATTCCCAAAAA	272	54
IRF8-2	F: TGGGAATTTTTGAGGGTATTTT<br>R: ACCTCCCTAACCAATAAAC	185	52

Table S7 CpG island amplification primers

Name	Primer sequences (5'-3')	Length (bp)	Temperature (℃)
IRF8-P1	F: GGACTAGTCGGGACAGGGGGATGC<br>R: CATGCCATGGCGCCGAAGGTGGAGGT	182	68
IRF8-P2	F: GGACTAGTCGTCTGCAGCATCGA<br>R: CATGCCATGGTTGCCACACTCACCG	420	68
