# Supplementary figures and images for: Hypomethylated interferon regulatory factor 8 recruits activating protein-2α to attenuate porcine epidemic diarrhea virus infection in porcine jejunum
Source: Front Immunol. 2023 Aug 1;14:1187144. doi: 10.3389/fimmu.2023.1187144 (PMC10427914; doi:10.3389/fimmu.2023.1187144)

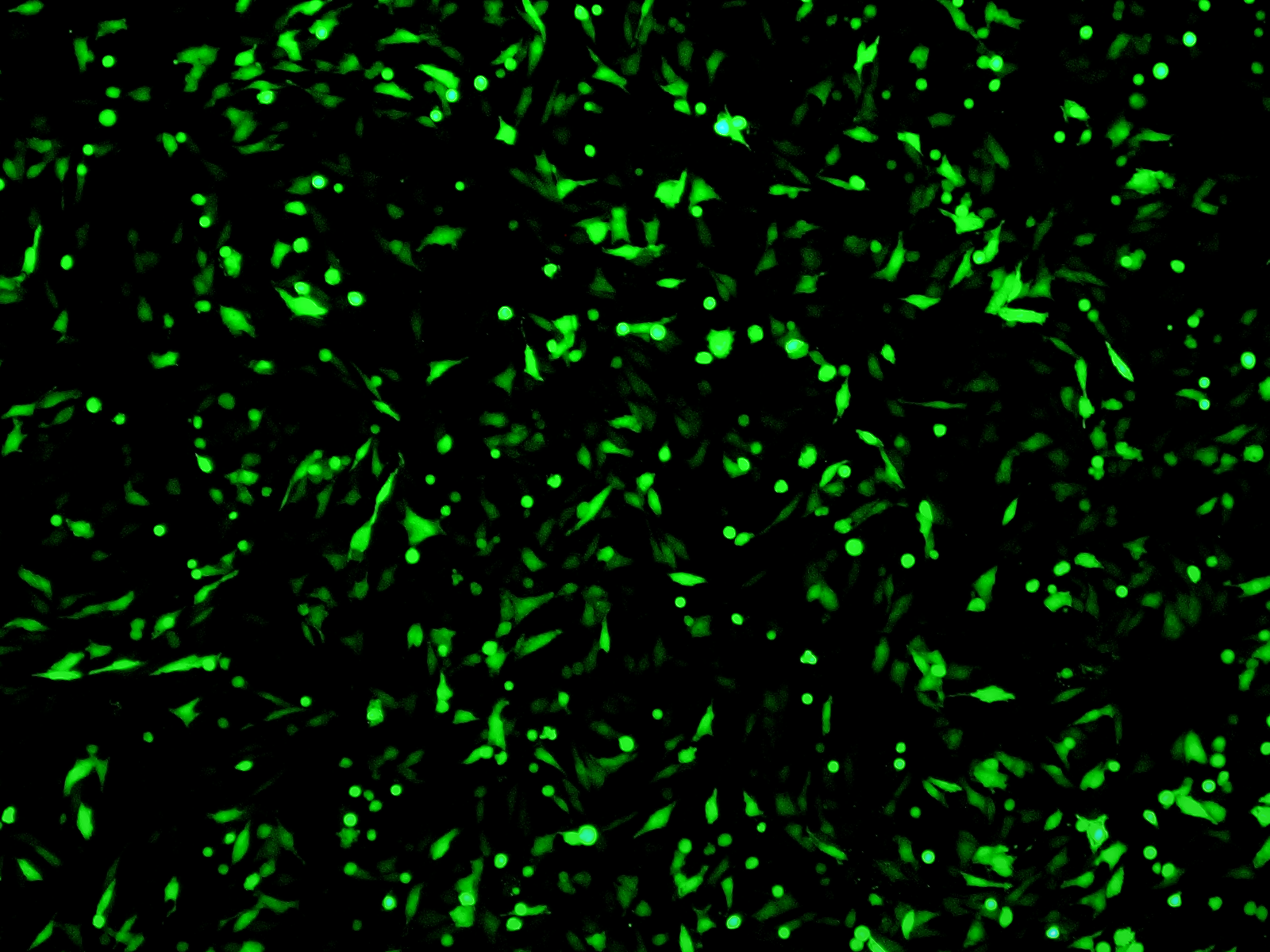

Supplement: Supplementary file 4 [file DataSheet_3.zip › Raw data of Microscopy images Figure 2/Figure 2B/IRF8-KO-EGFP.tif]

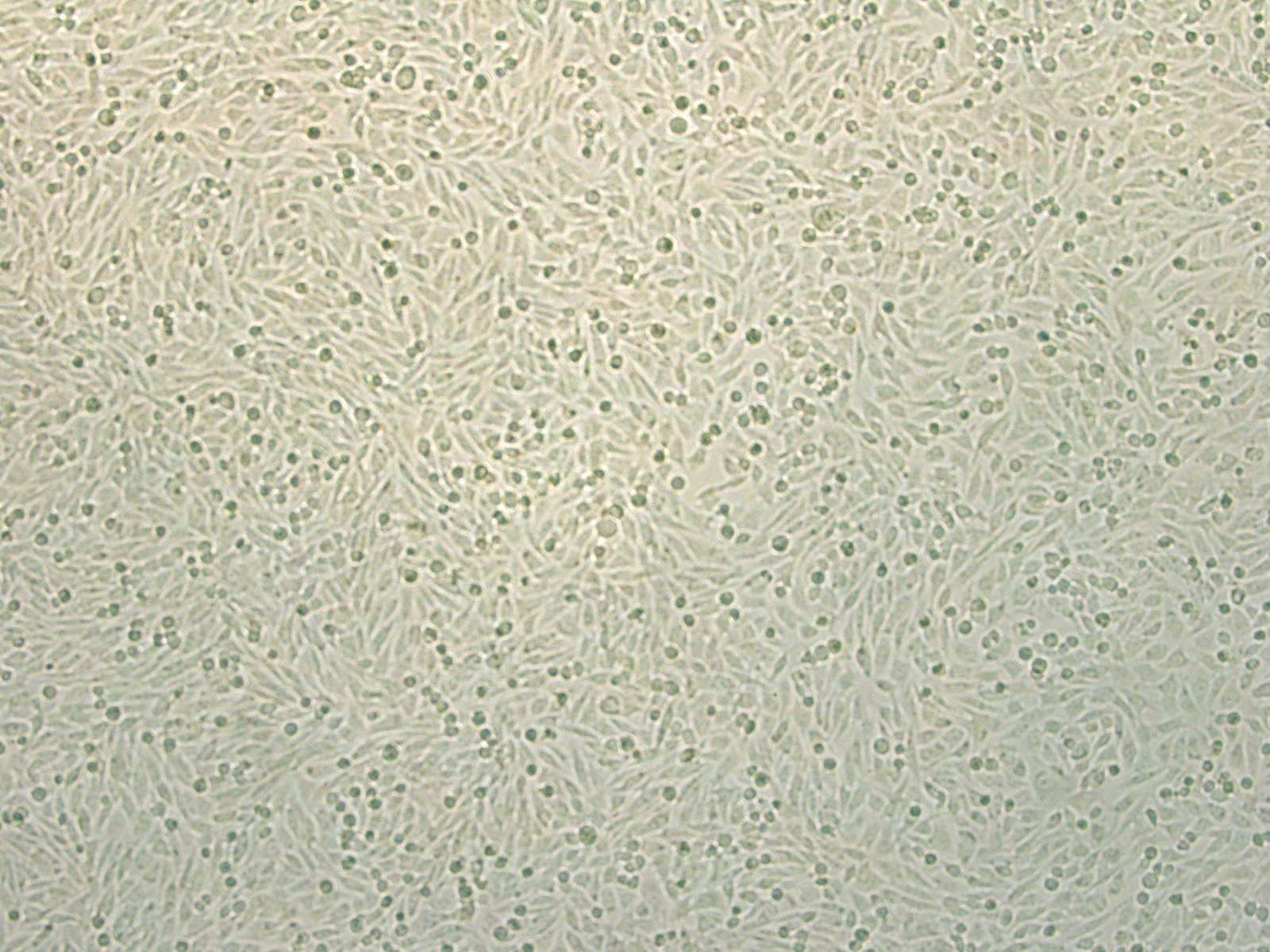

Supplement: Supplementary file 4 [file DataSheet_3.zip › Raw data of Microscopy images Figure 2/Figure 2B/IRF8-KO-white.tif]

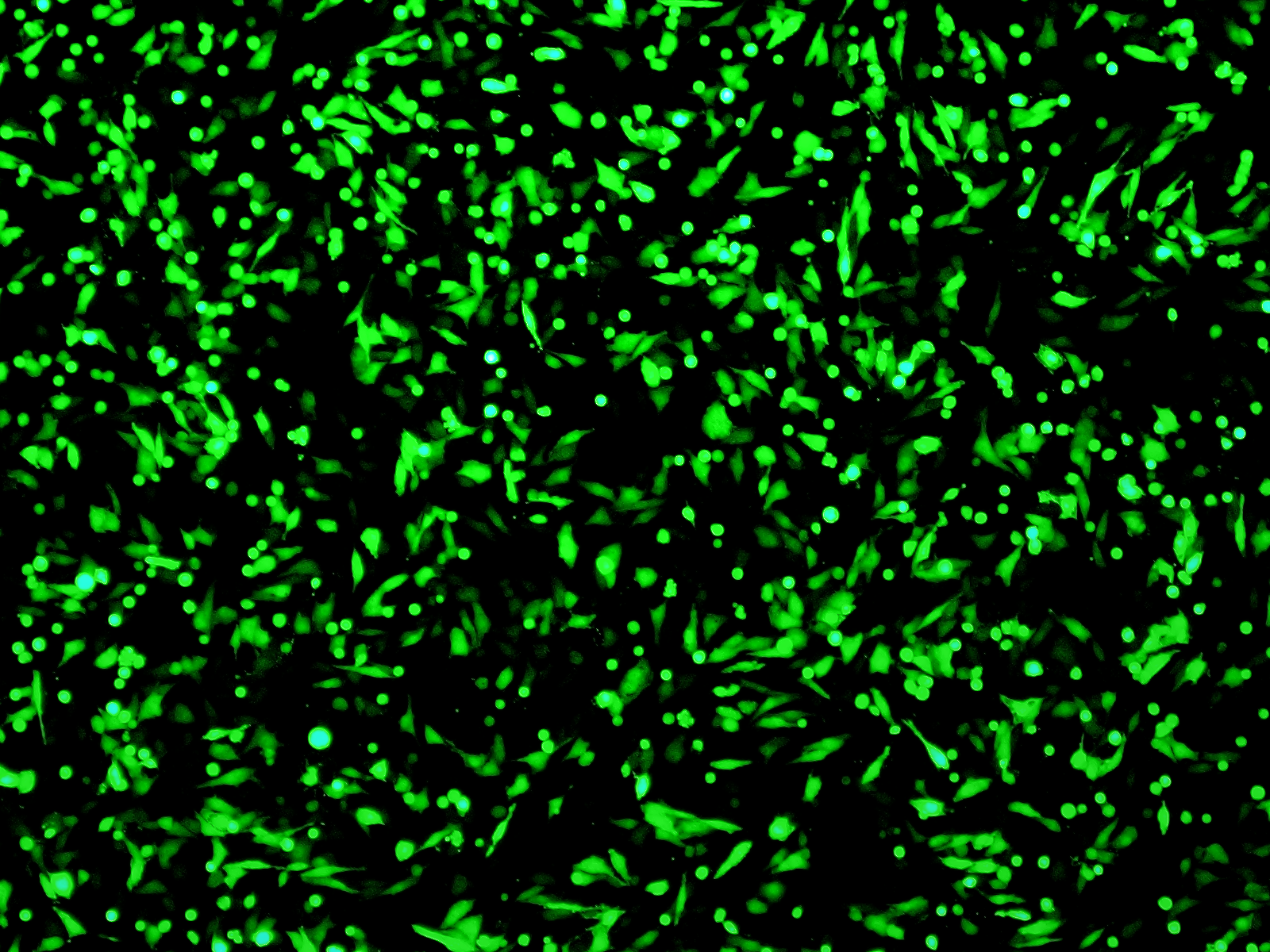

Supplement: Supplementary file 4 [file DataSheet_3.zip › Raw data of Microscopy images Figure 2/Figure 2B/pGK1.2-EGFP.tif]

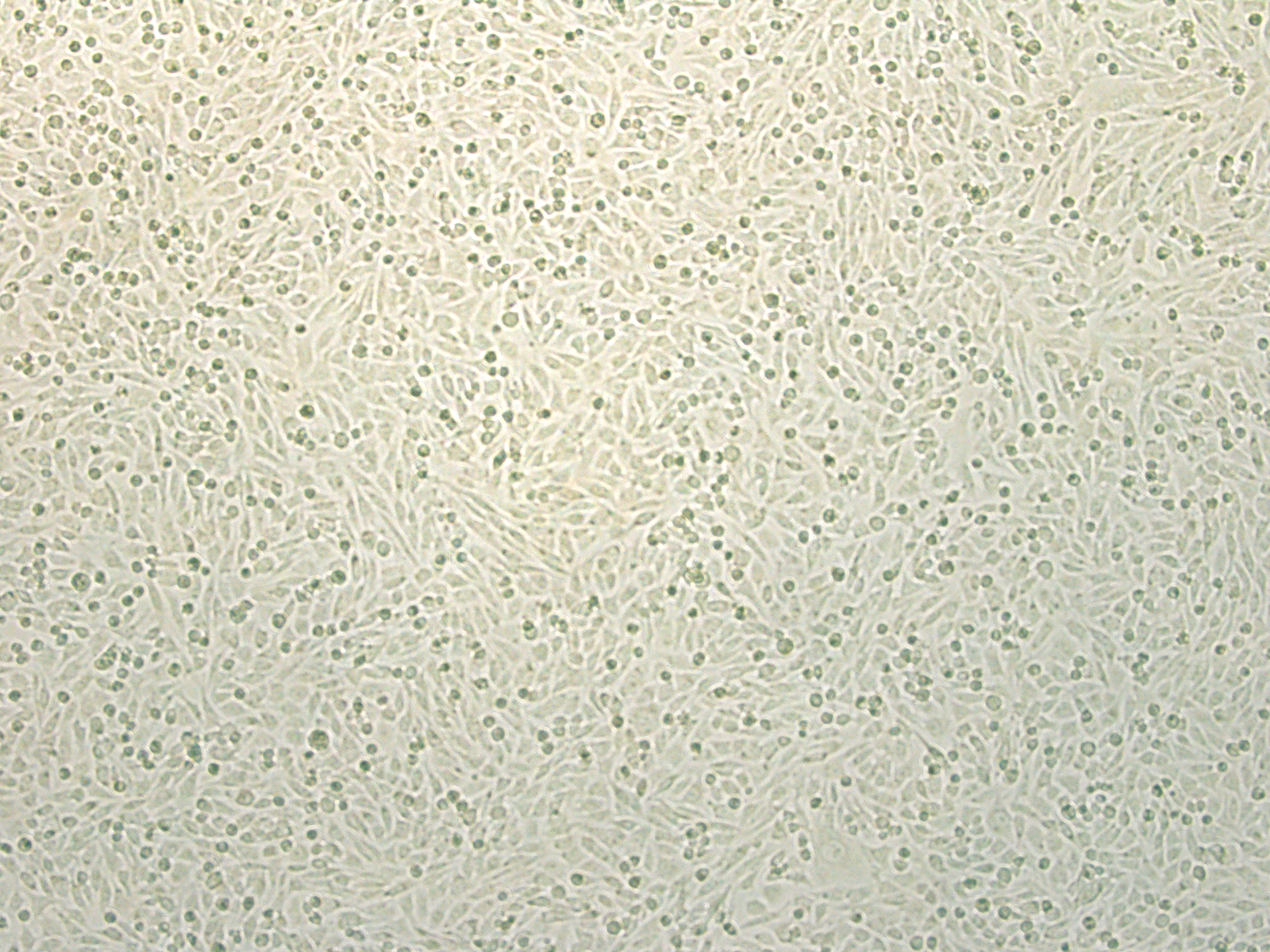

Supplement: Supplementary file 4 [file DataSheet_3.zip › Raw data of Microscopy images Figure 2/Figure 2B/pGK1.2-white.tif]

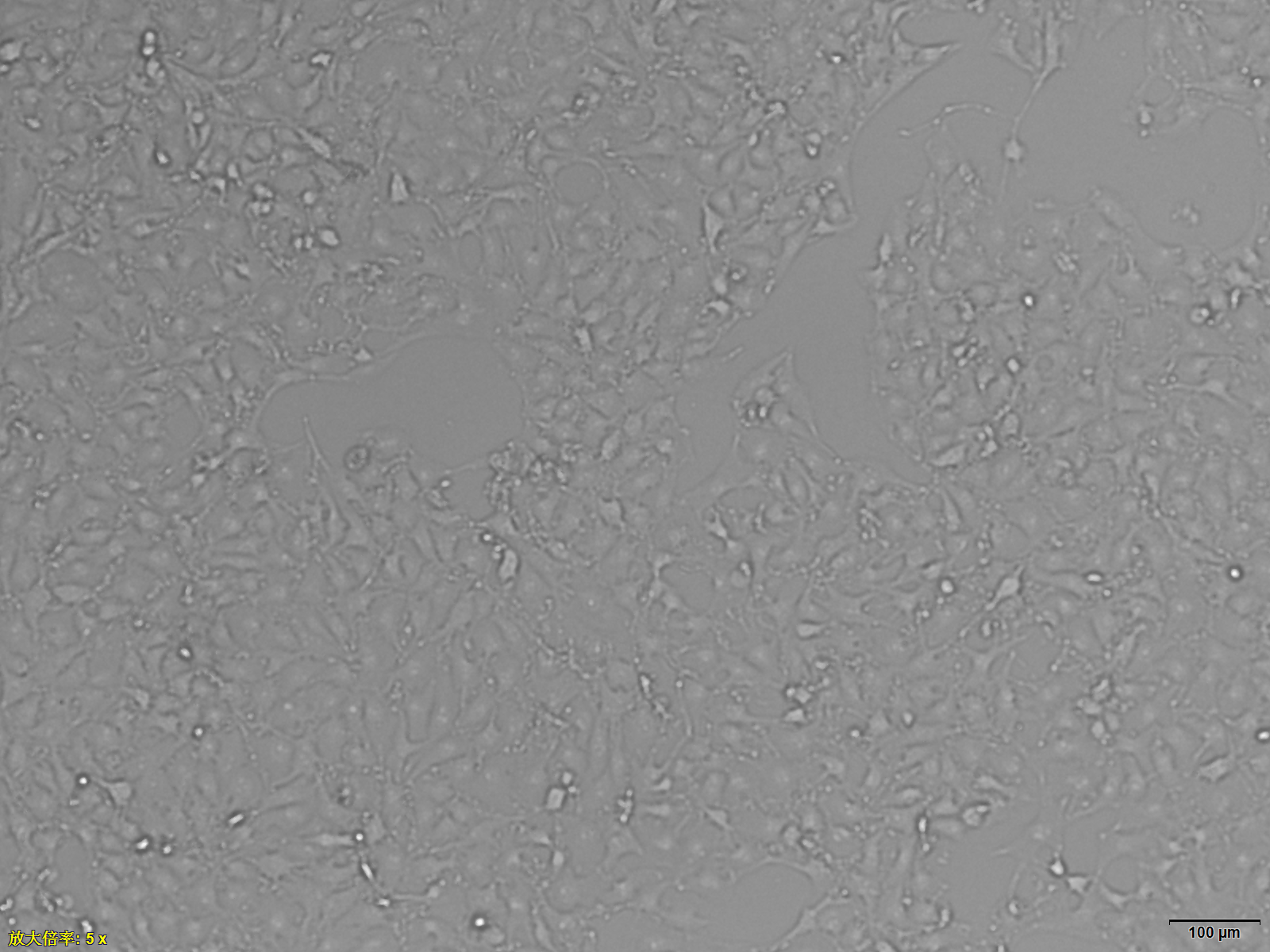

Supplement: Supplementary file 4 [file DataSheet_3.zip › Raw data of Microscopy images Figure 2/Figure 2D/IRF8-KO-0h.tif]

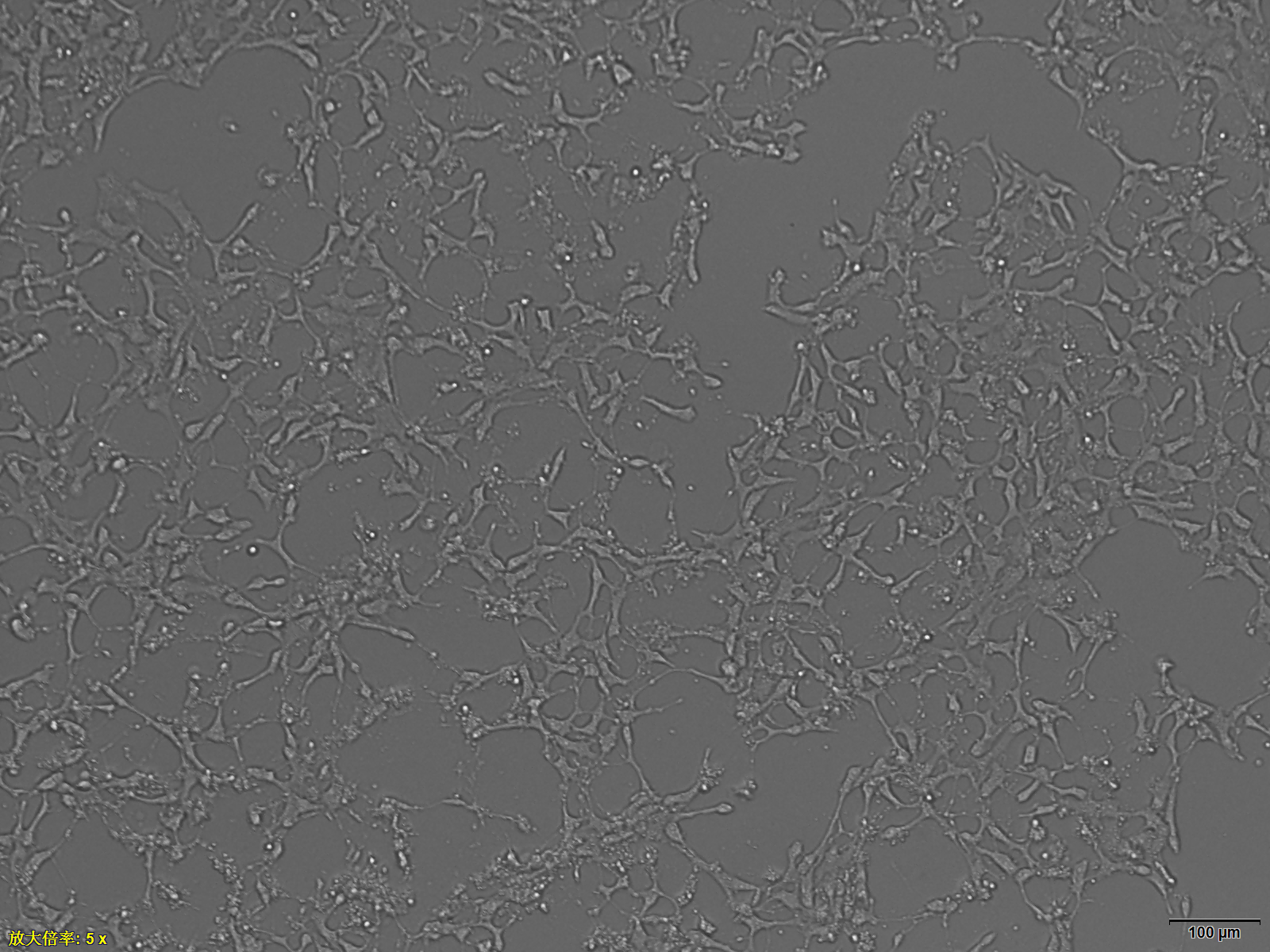

Supplement: Supplementary file 4 [file DataSheet_3.zip › Raw data of Microscopy images Figure 2/Figure 2D/IRF8-KO-24h.tif]

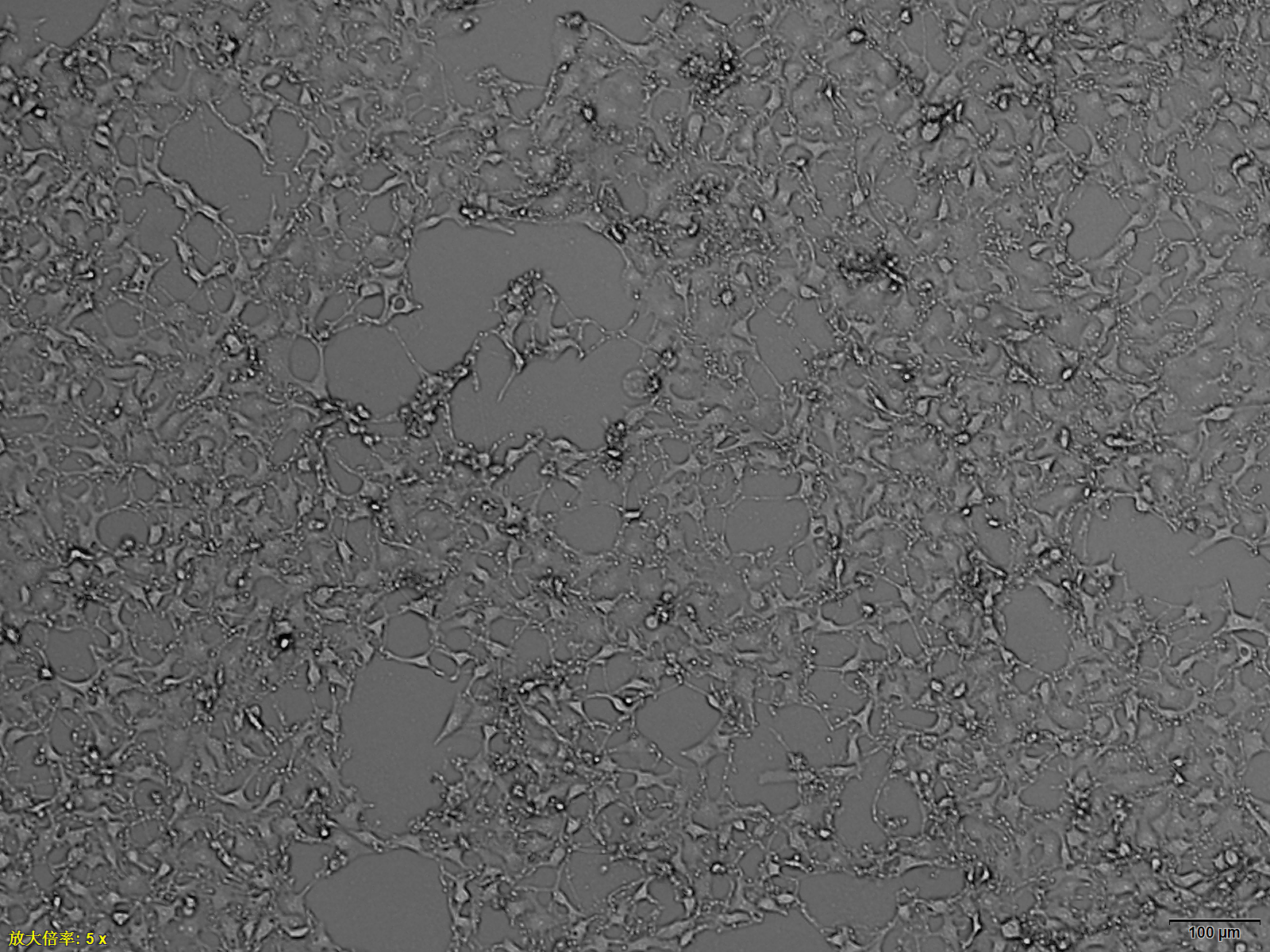

Supplement: Supplementary file 4 [file DataSheet_3.zip › Raw data of Microscopy images Figure 2/Figure 2D/IRF8-KO-48h.tif]

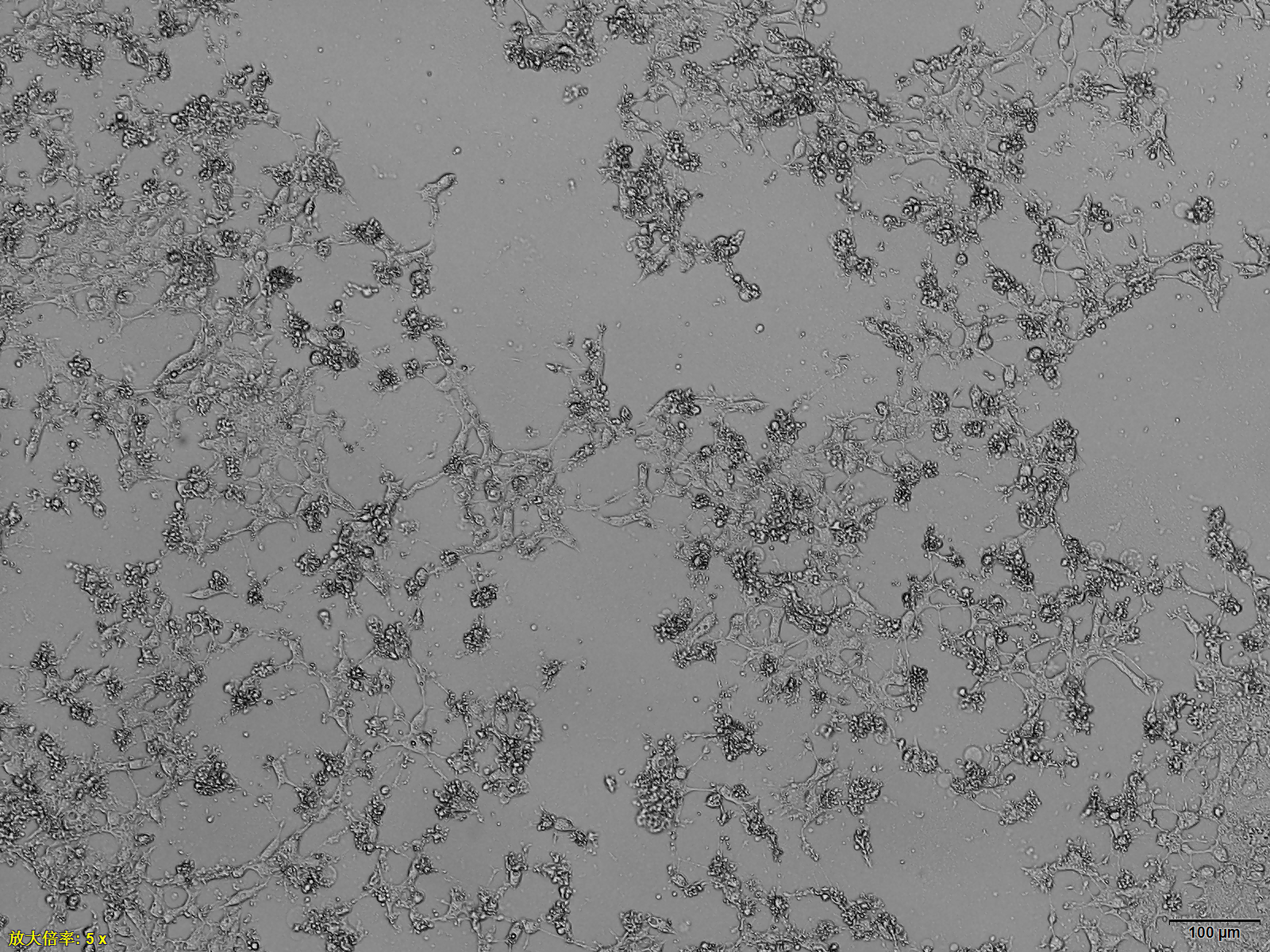

Supplement: Supplementary file 4 [file DataSheet_3.zip › Raw data of Microscopy images Figure 2/Figure 2D/IRF8-KO-72h.tif]

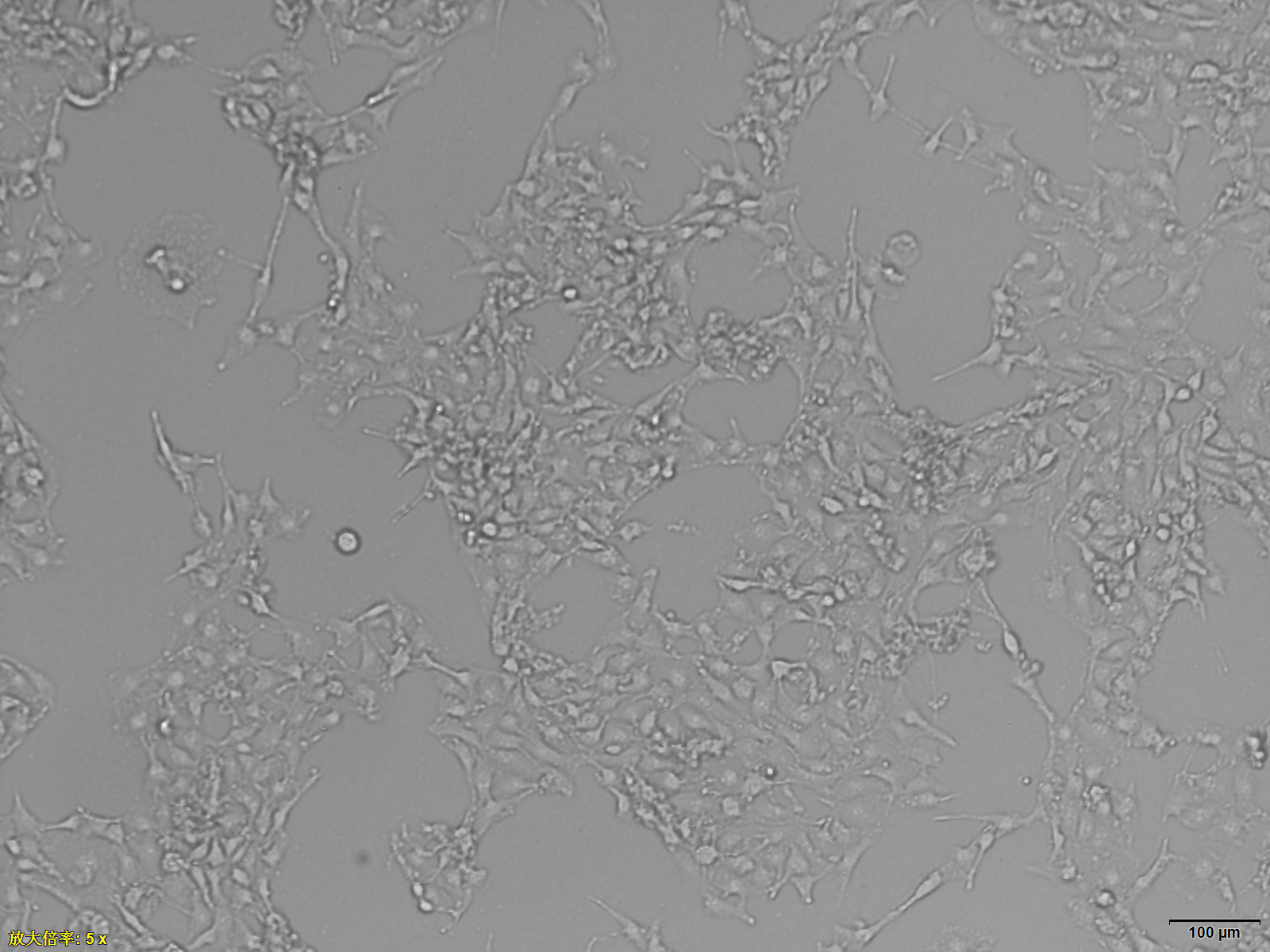

Supplement: Supplementary file 4 [file DataSheet_3.zip › Raw data of Microscopy images Figure 2/Figure 2D/pGK1.2-0h.tif]

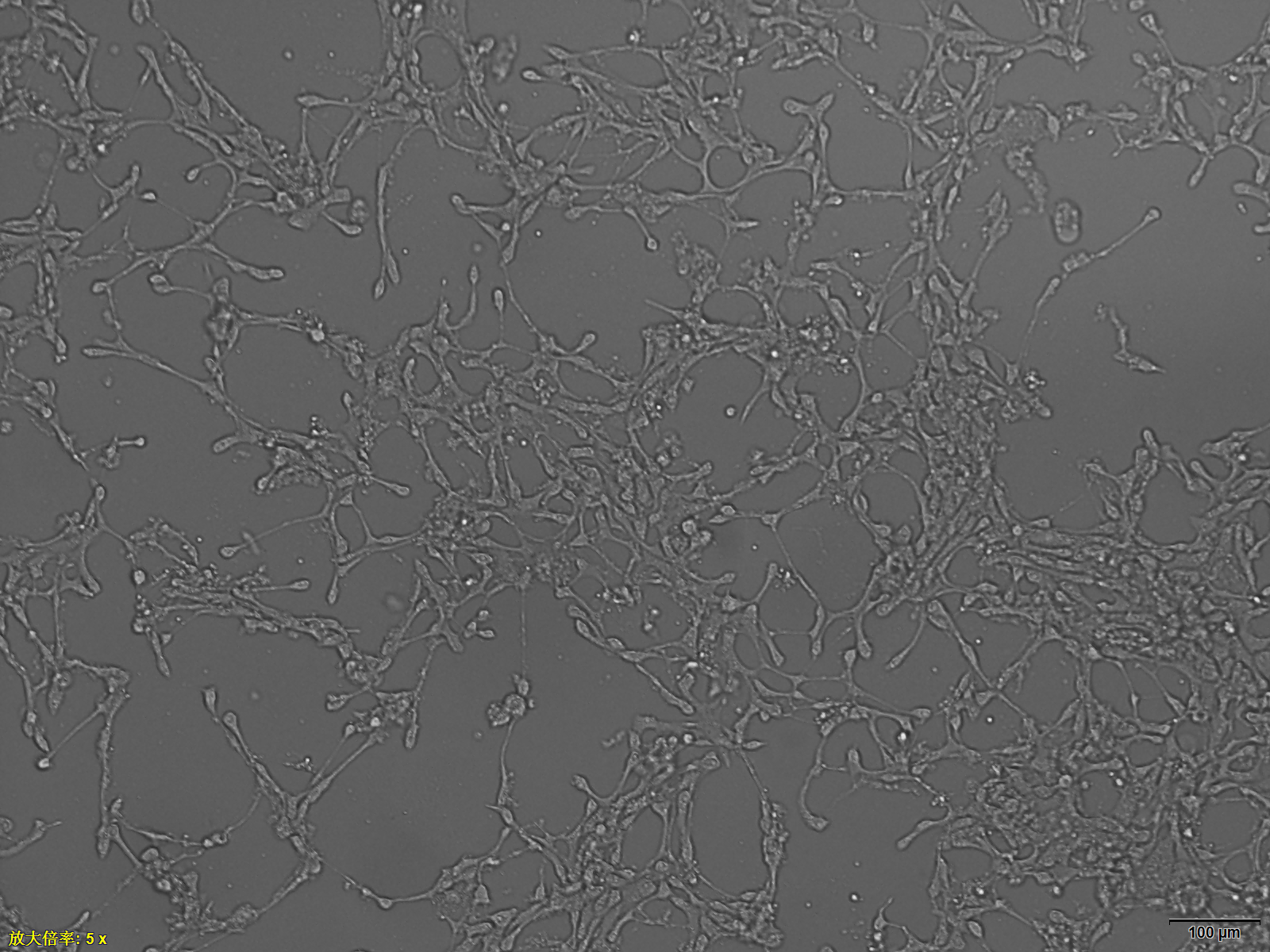

Supplement: Supplementary file 4 [file DataSheet_3.zip › Raw data of Microscopy images Figure 2/Figure 2D/pGK1.2-24h.tif]

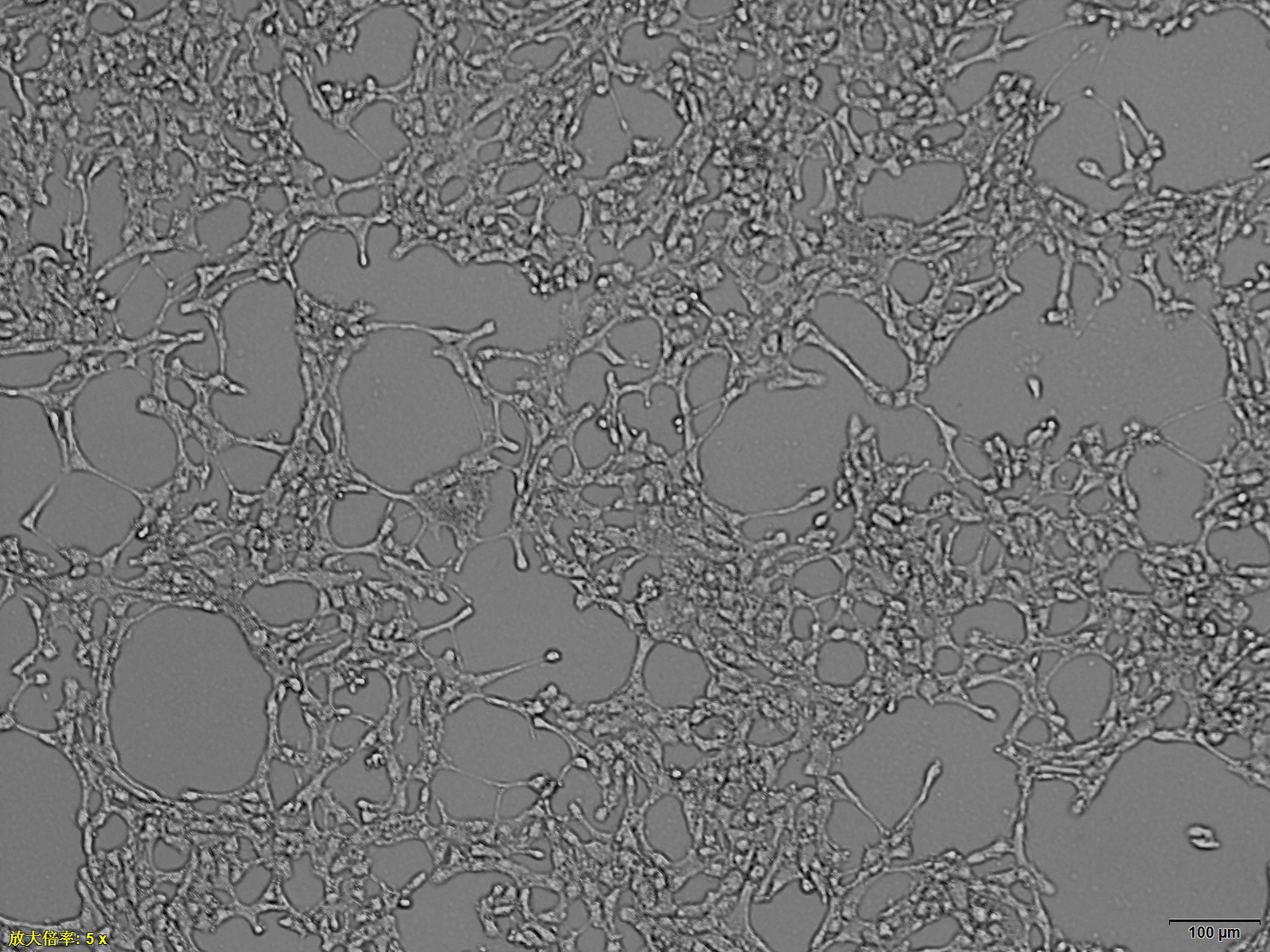

Supplement: Supplementary file 4 [file DataSheet_3.zip › Raw data of Microscopy images Figure 2/Figure 2D/pGK1.2-48h.tif]

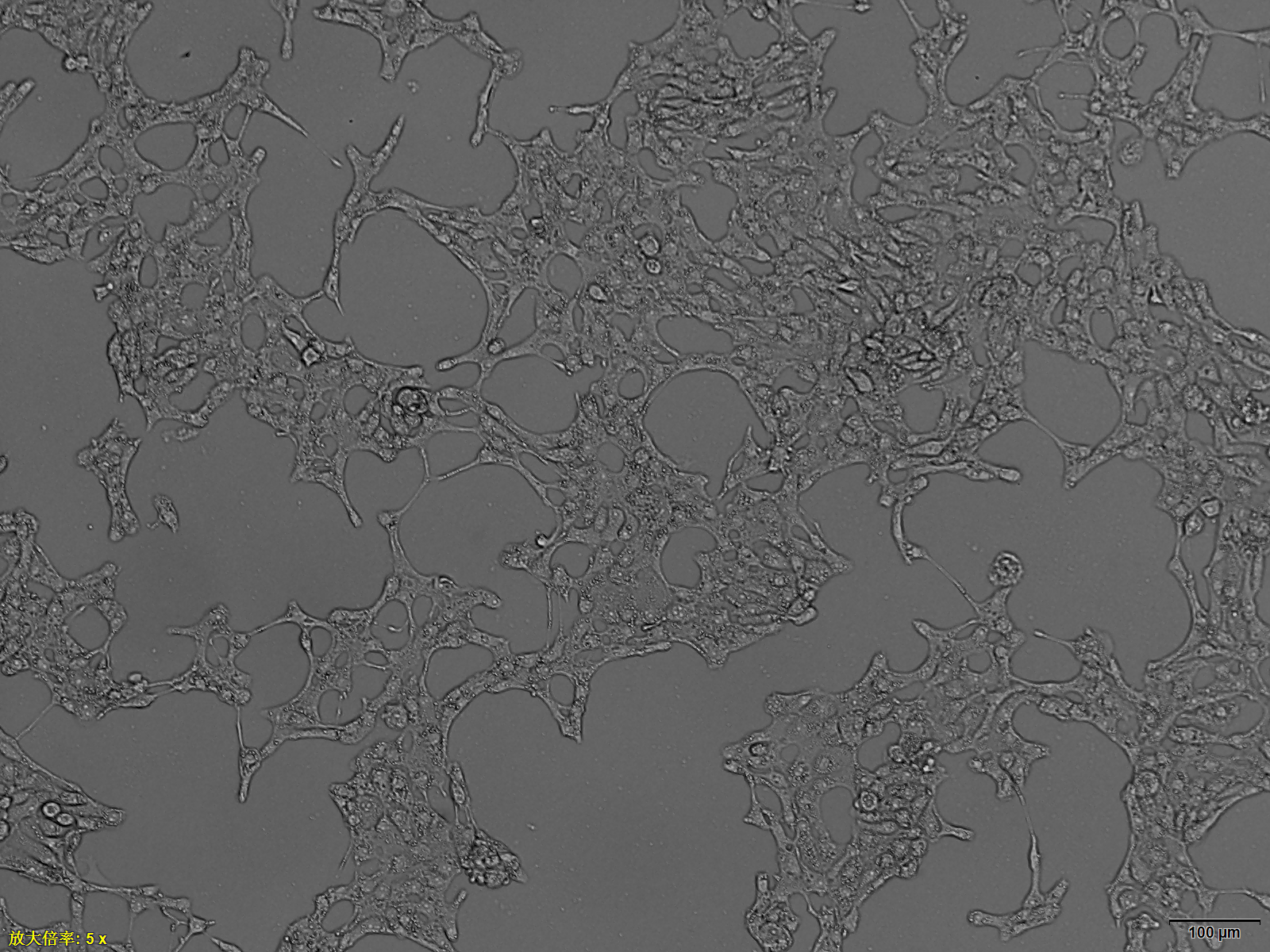

Supplement: Supplementary file 4 [file DataSheet_3.zip › Raw data of Microscopy images Figure 2/Figure 2D/pGK1.2-72h.tif]

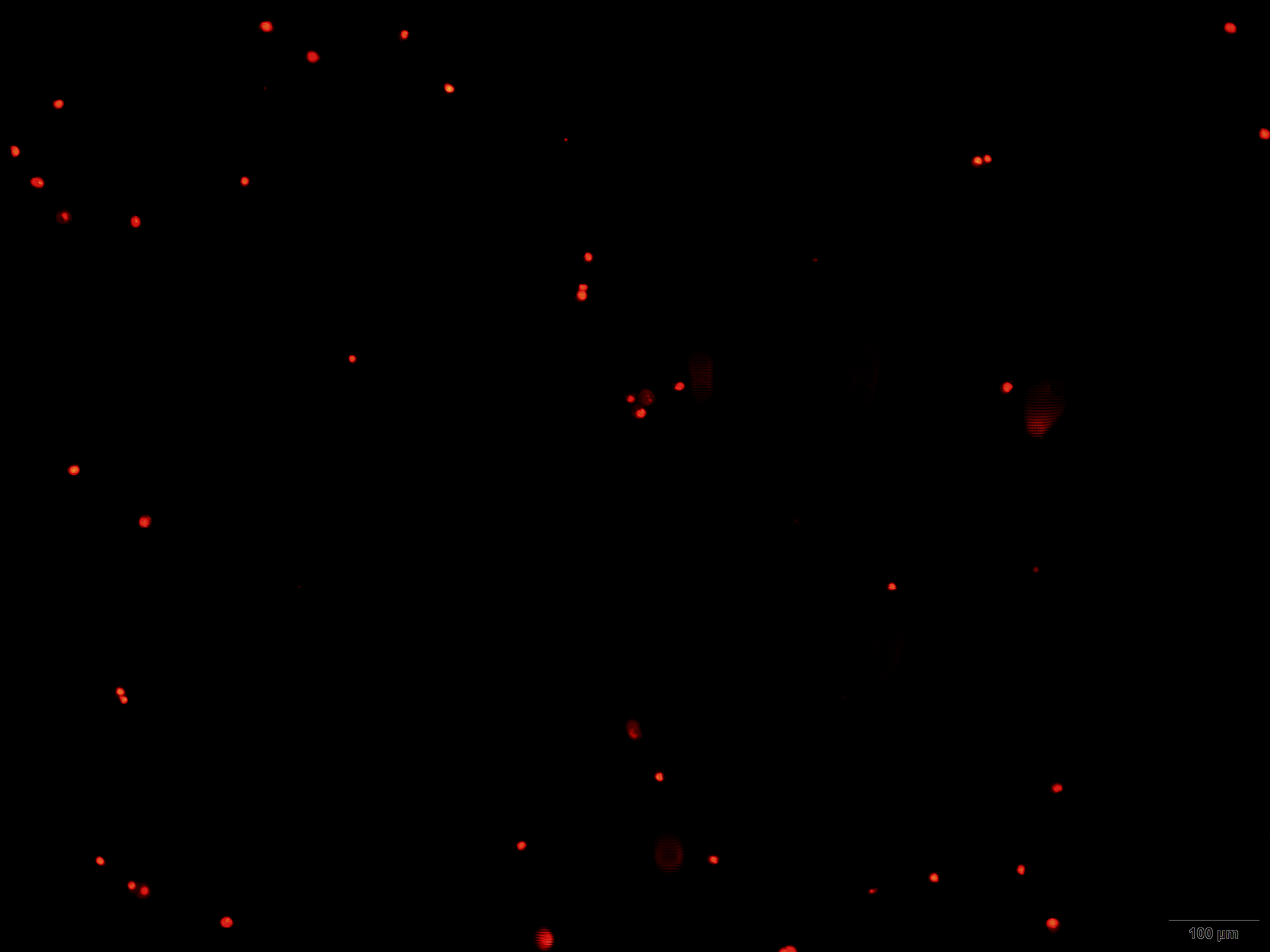

Supplement: Supplementary file 5 [file DataSheet_4.zip › Raw data of Microscopy images Figure 7/Figure 7F/Dead-IRF8-OE+PEDV.tif]

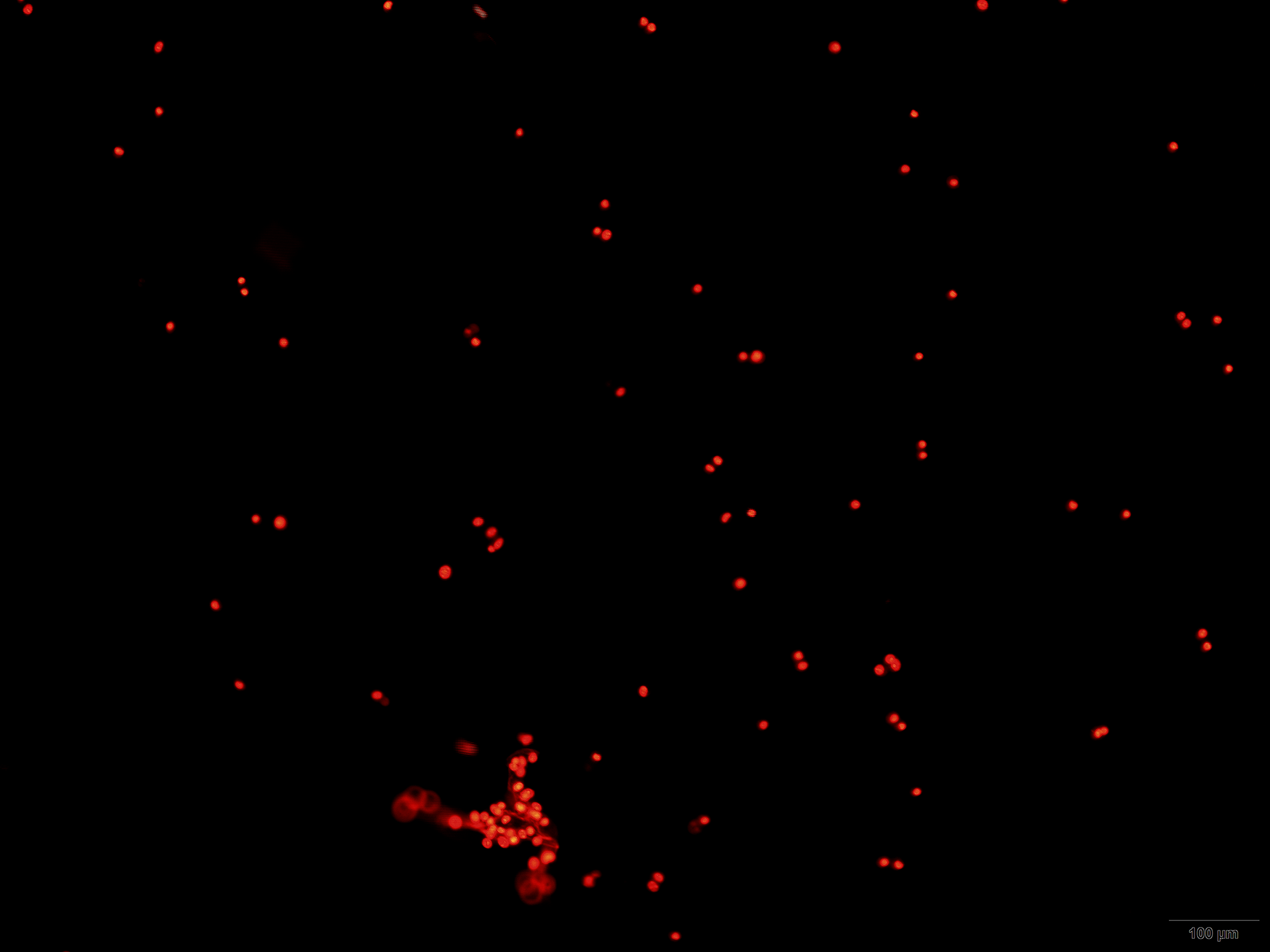

Supplement: Supplementary file 5 [file DataSheet_4.zip › Raw data of Microscopy images Figure 7/Figure 7F/Dead-PEDV.tif]

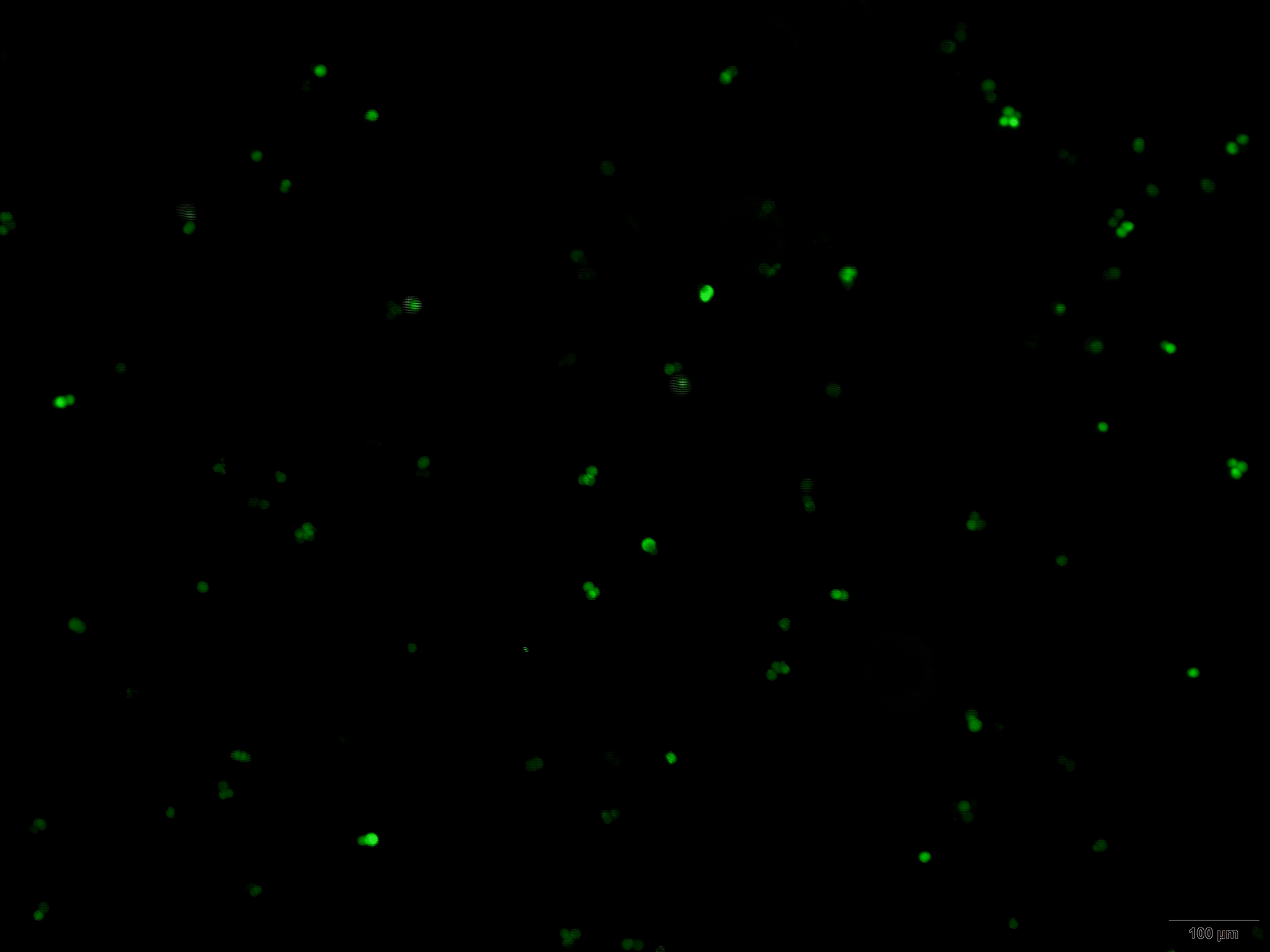

Supplement: Supplementary file 5 [file DataSheet_4.zip › Raw data of Microscopy images Figure 7/Figure 7F/Live-IRF8-OE+PEDV.tif]

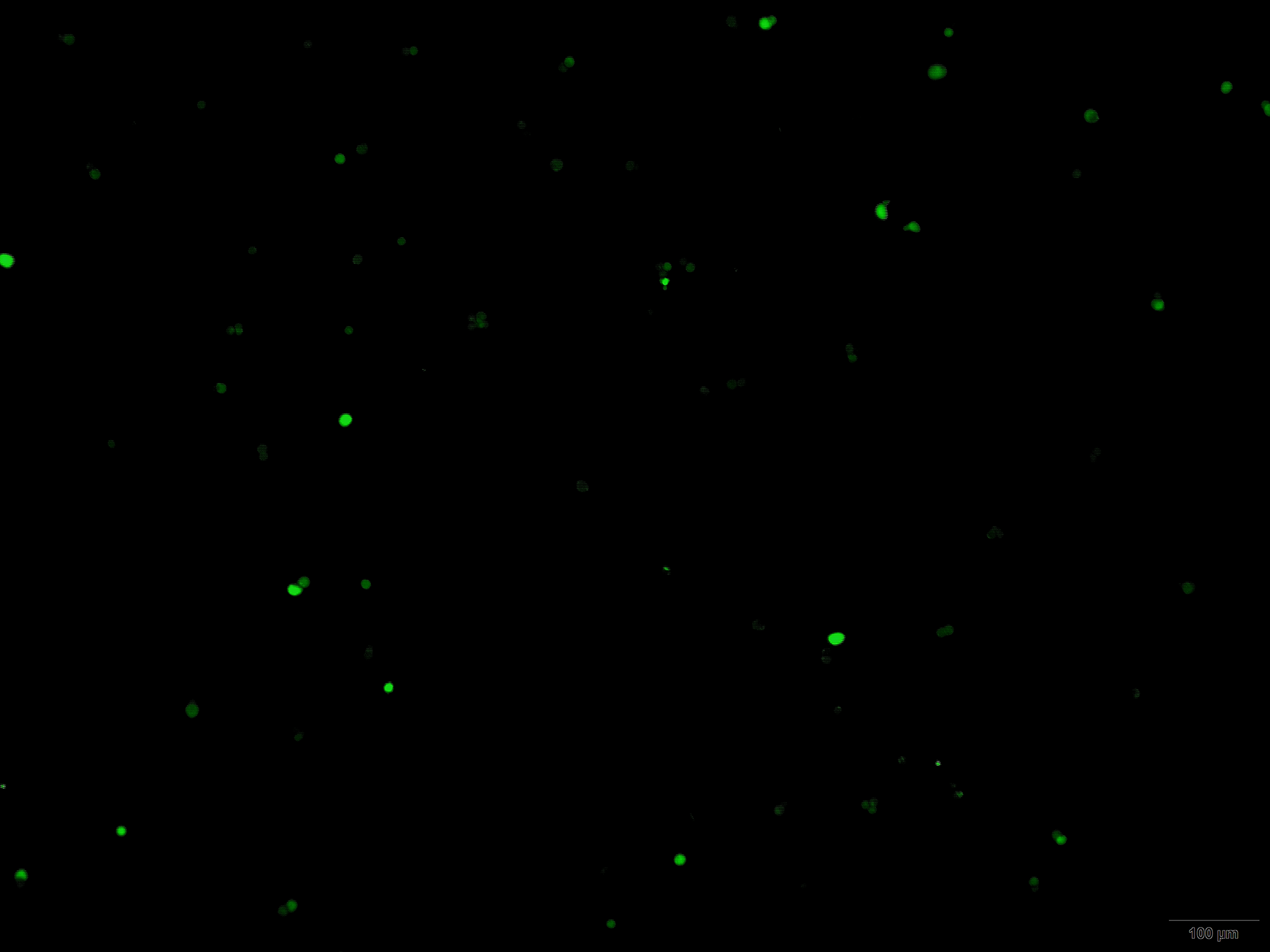

Supplement: Supplementary file 5 [file DataSheet_4.zip › Raw data of Microscopy images Figure 7/Figure 7F/Live-PEDV.tif]

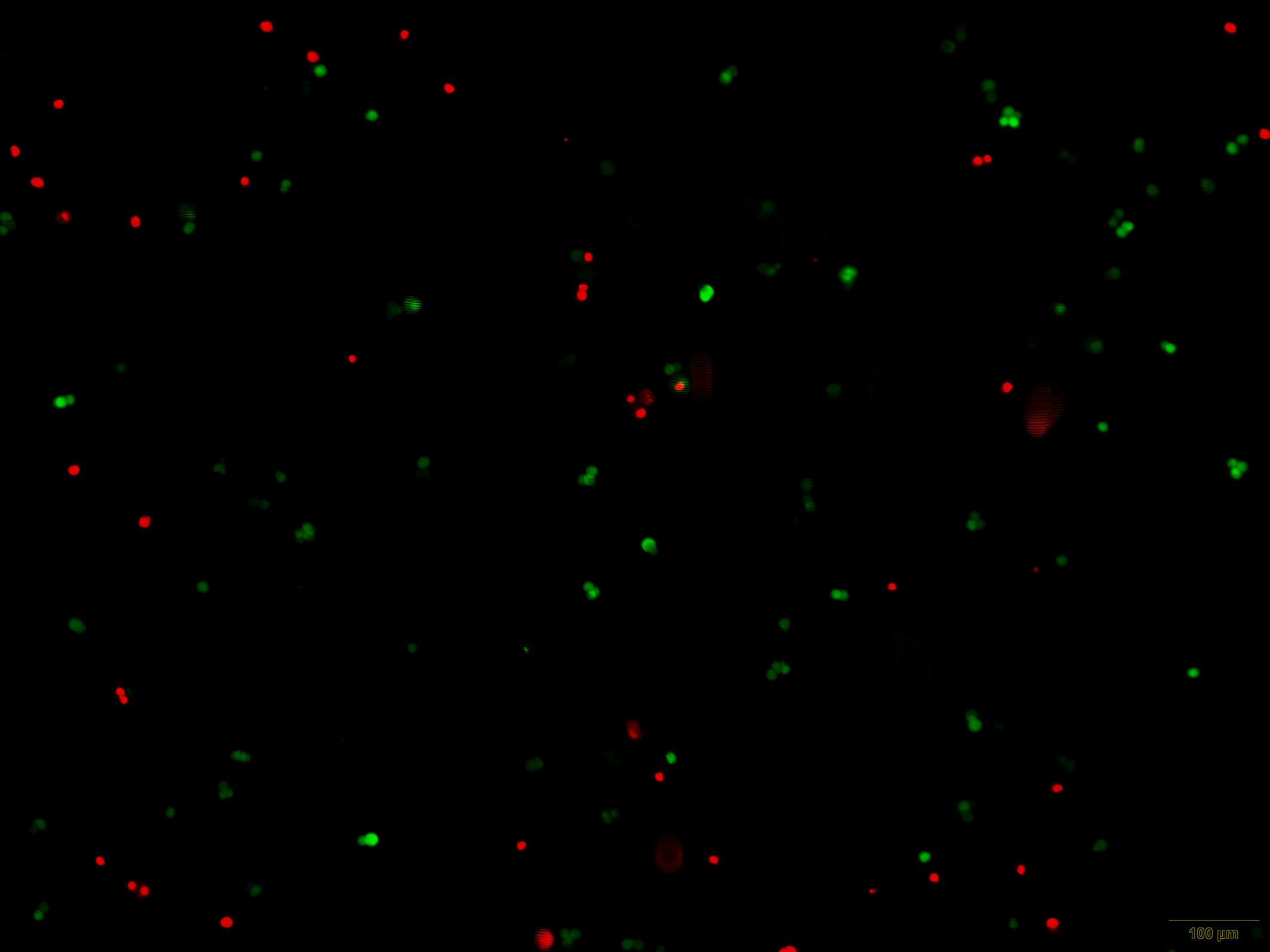

Supplement: Supplementary file 5 [file DataSheet_4.zip › Raw data of Microscopy images Figure 7/Figure 7F/Merge-IRF8-OE+PEDV.tif]

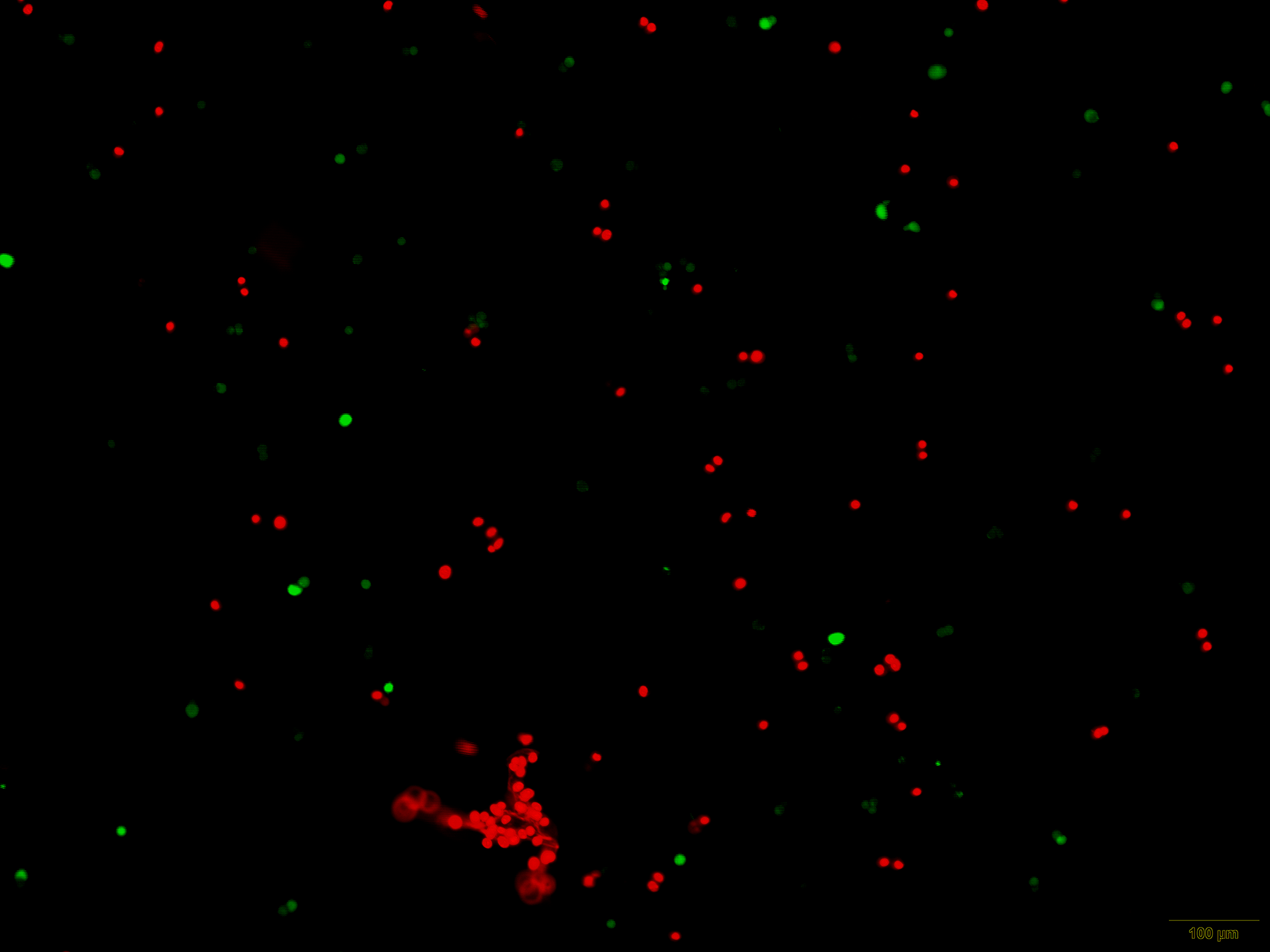

Supplement: Supplementary file 5 [file DataSheet_4.zip › Raw data of Microscopy images Figure 7/Figure 7F/Merge-PEDV.tif]

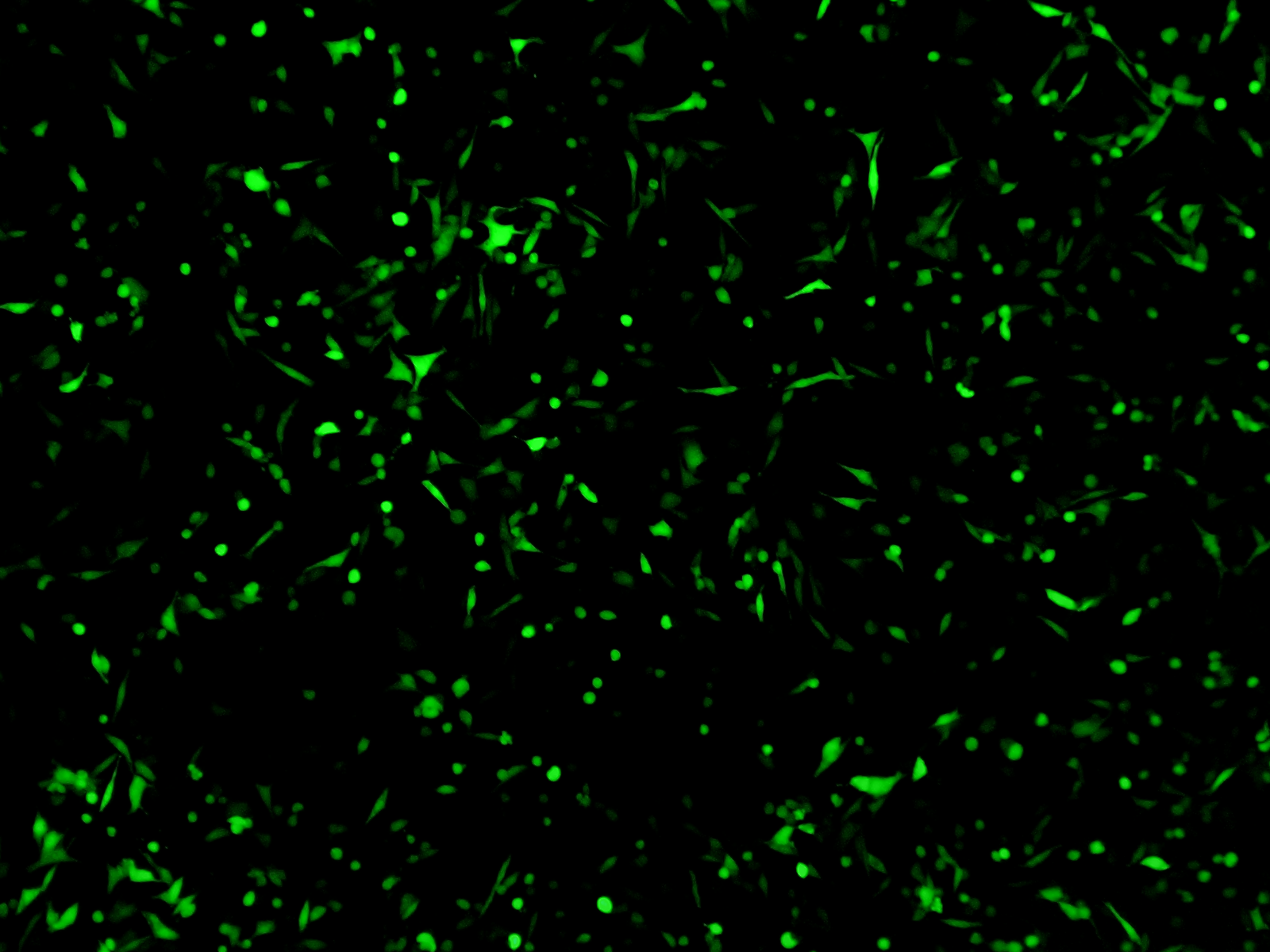

Supplement: Supplementary file 6 [file DataSheet_5.zip › Raw data of Microscopy images Supplymentary Figure 2/Supplementary Figure 2A/IRF8-OE-EGPF.tif]

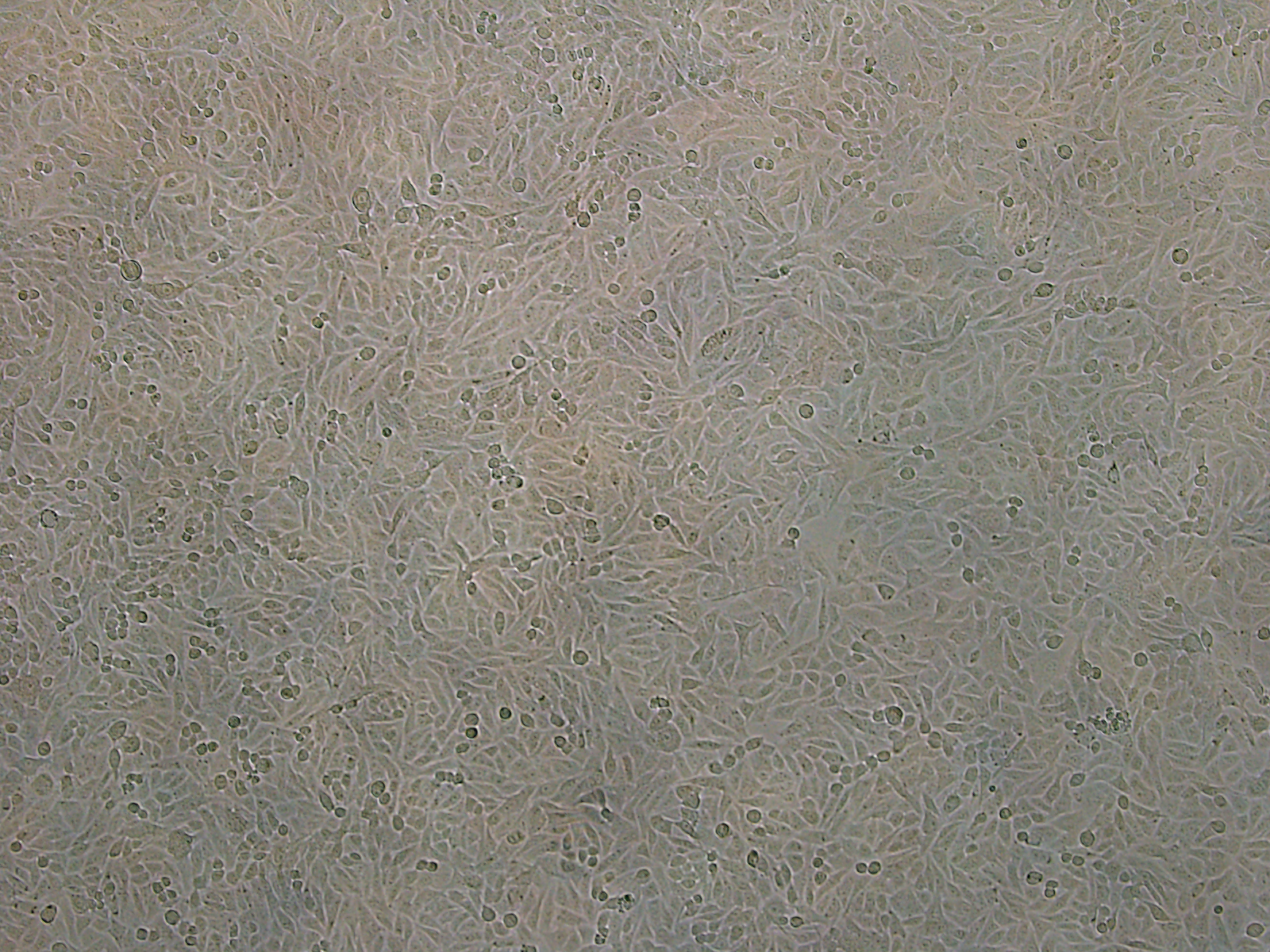

Supplement: Supplementary file 6 [file DataSheet_5.zip › Raw data of Microscopy images Supplymentary Figure 2/Supplementary Figure 2A/IRF8-OE-white.tif]

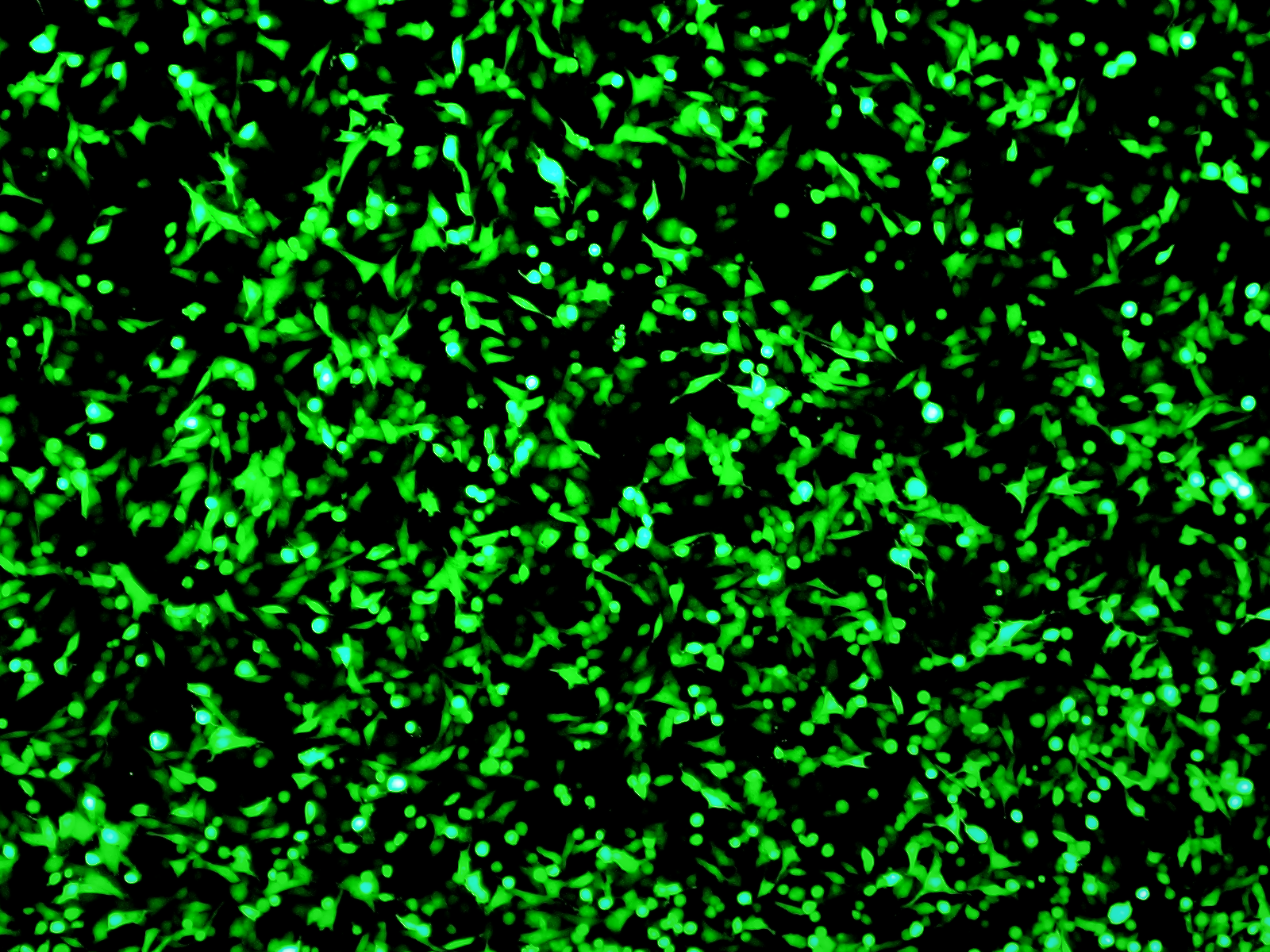

Supplement: Supplementary file 6 [file DataSheet_5.zip › Raw data of Microscopy images Supplymentary Figure 2/Supplementary Figure 2A/pcDNA3.1-EGFP.tif]

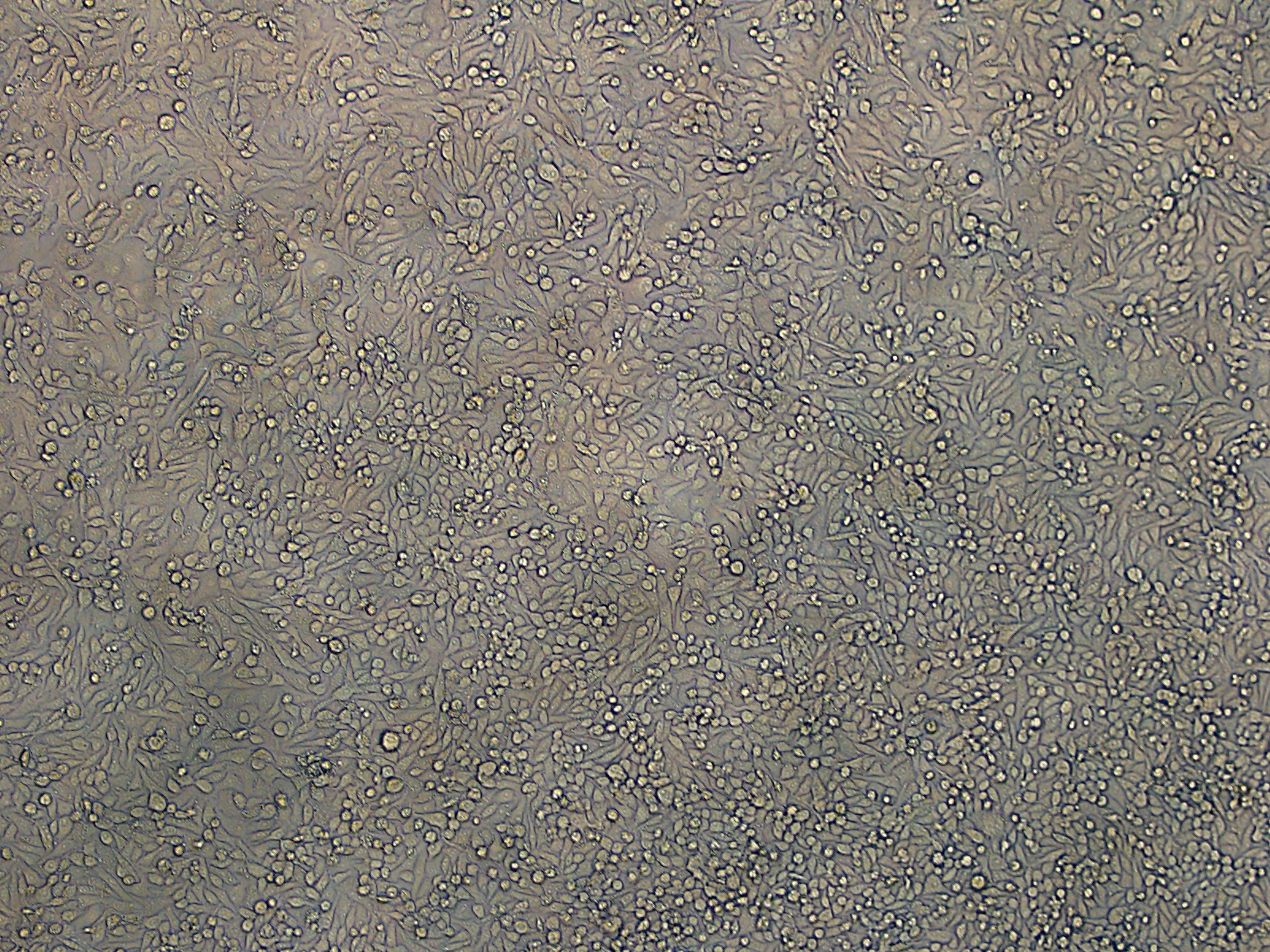

Supplement: Supplementary file 6 [file DataSheet_5.zip › Raw data of Microscopy images Supplymentary Figure 2/Supplementary Figure 2A/pcDNA3.1-white.tif]

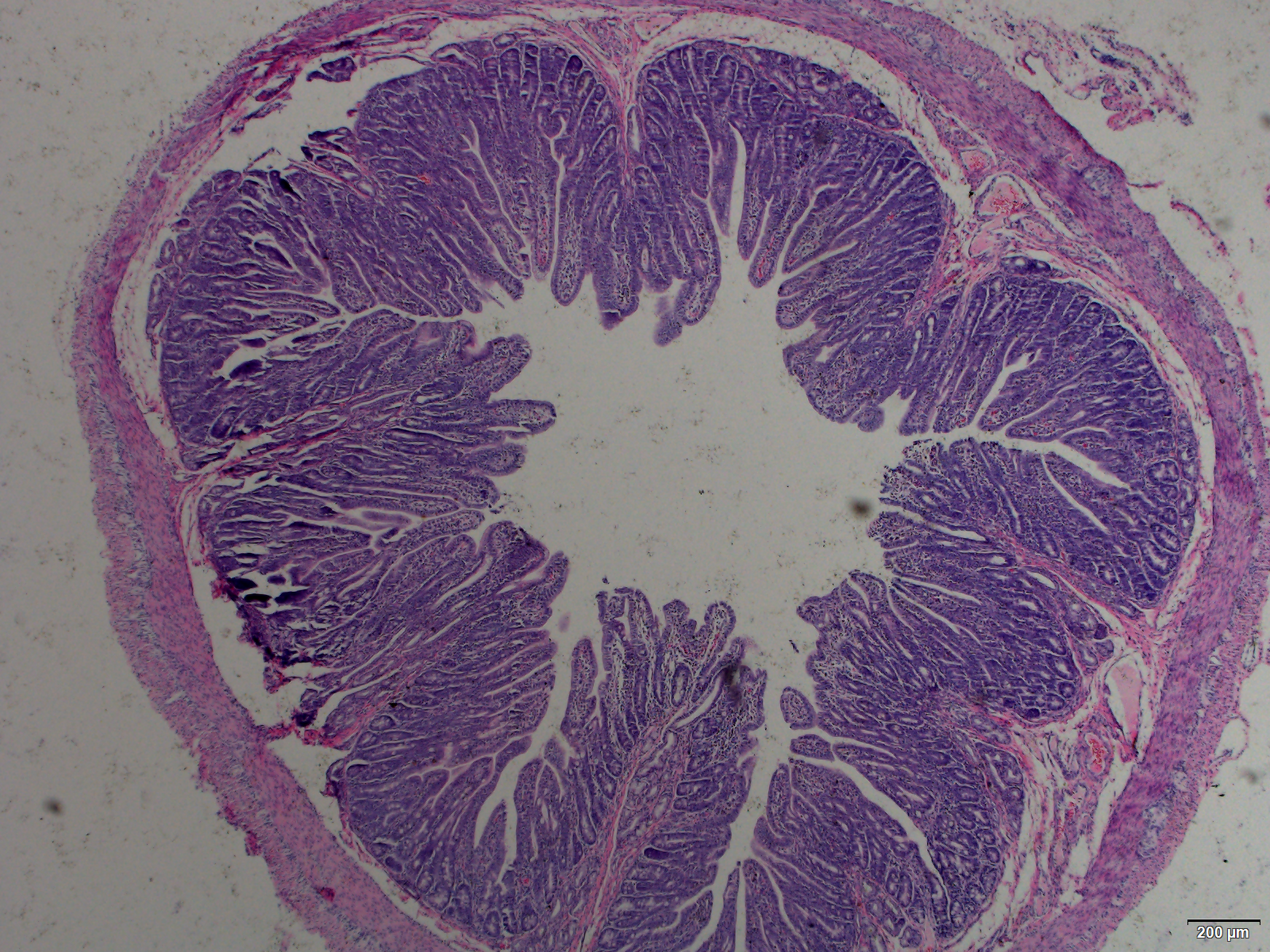

Supplement: Supplementary file 7 [file DataSheet_6.zip › Raw data of Microscopy images Figure 1A/Ctrl-Duodenum.tif]

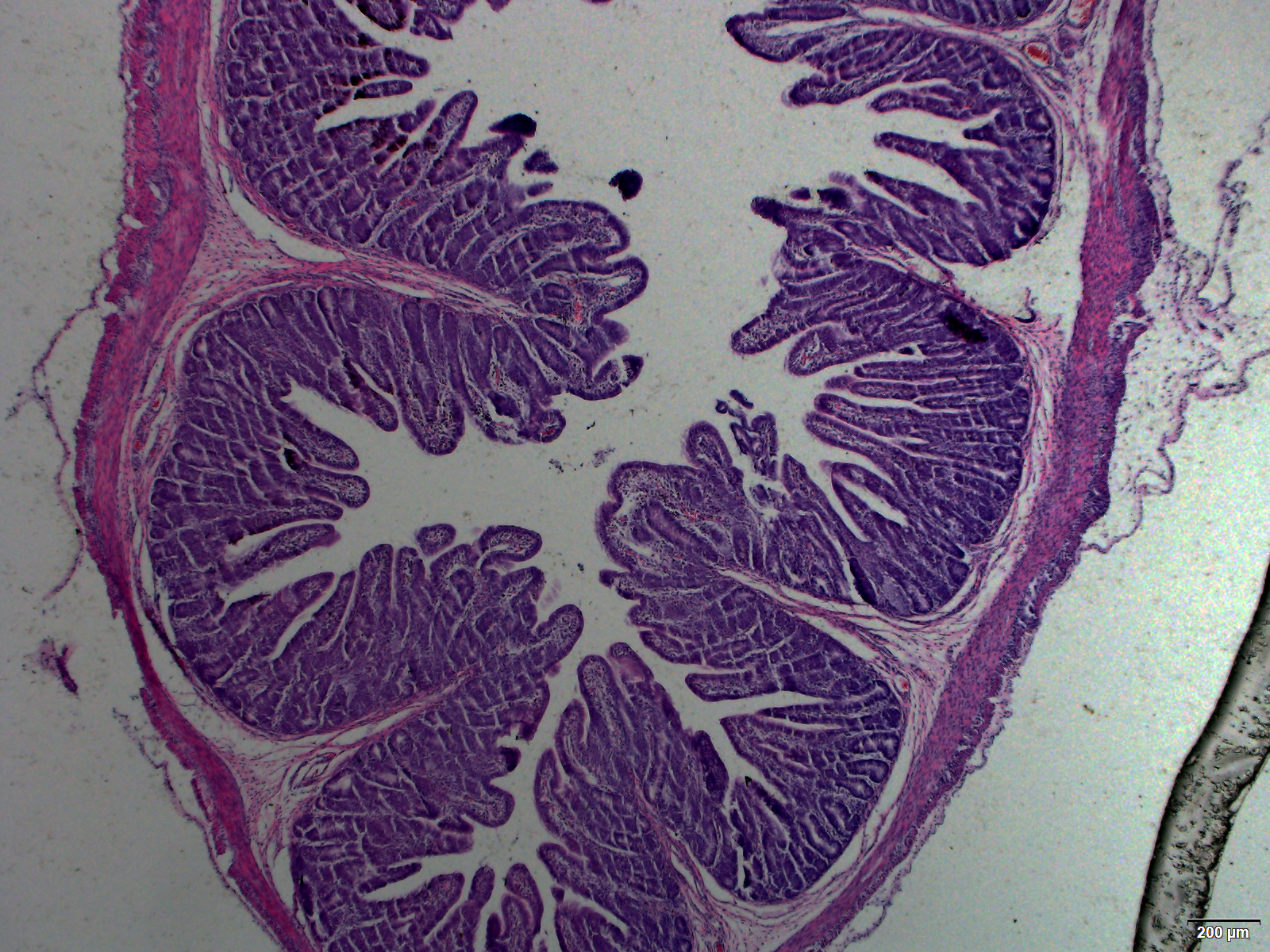

Supplement: Supplementary file 7 [file DataSheet_6.zip › Raw data of Microscopy images Figure 1A/Ctrl-Ileum.tif]

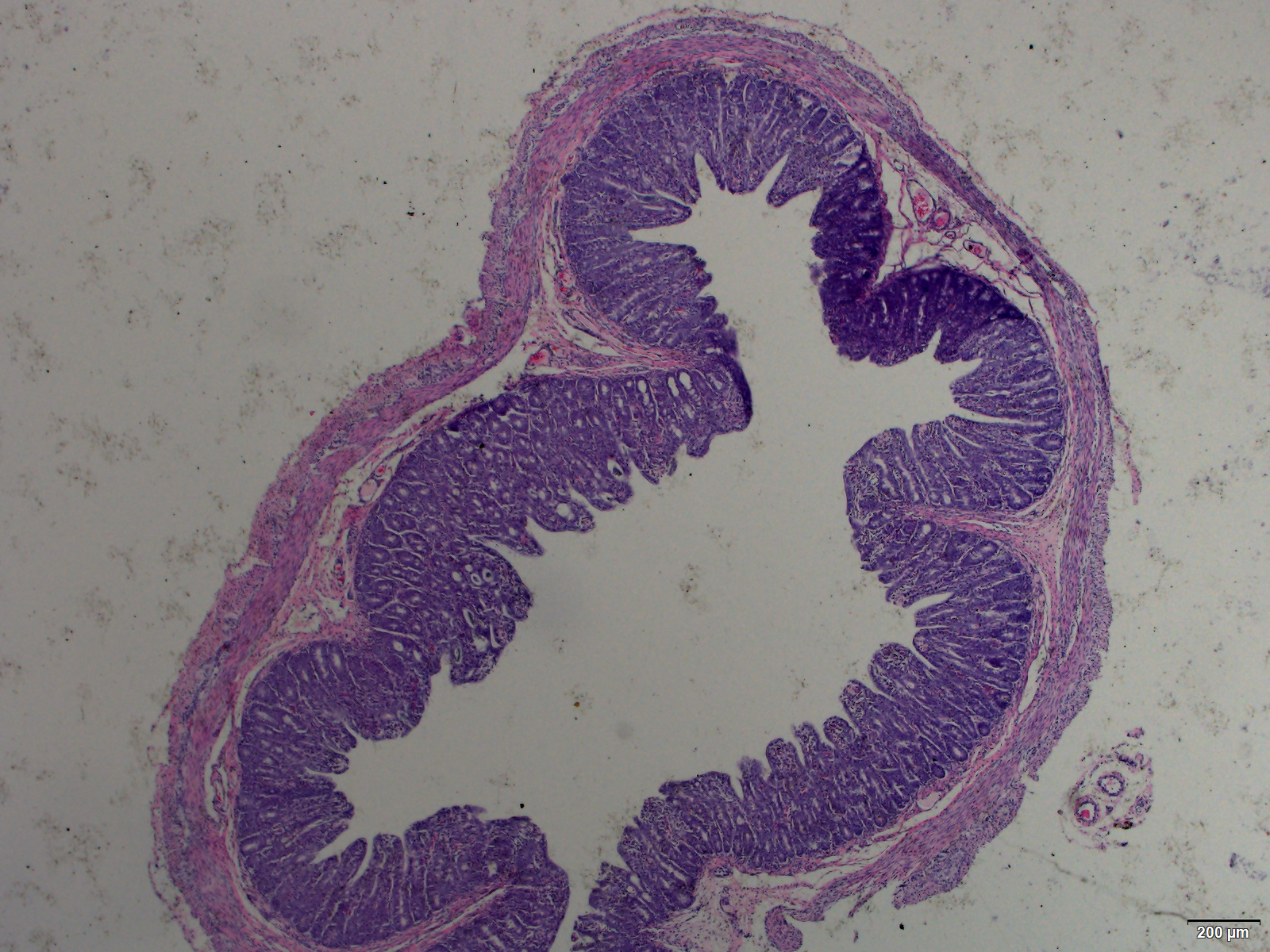

Supplement: Supplementary file 7 [file DataSheet_6.zip › Raw data of Microscopy images Figure 1A/Ctrl-Jejunum.tif]

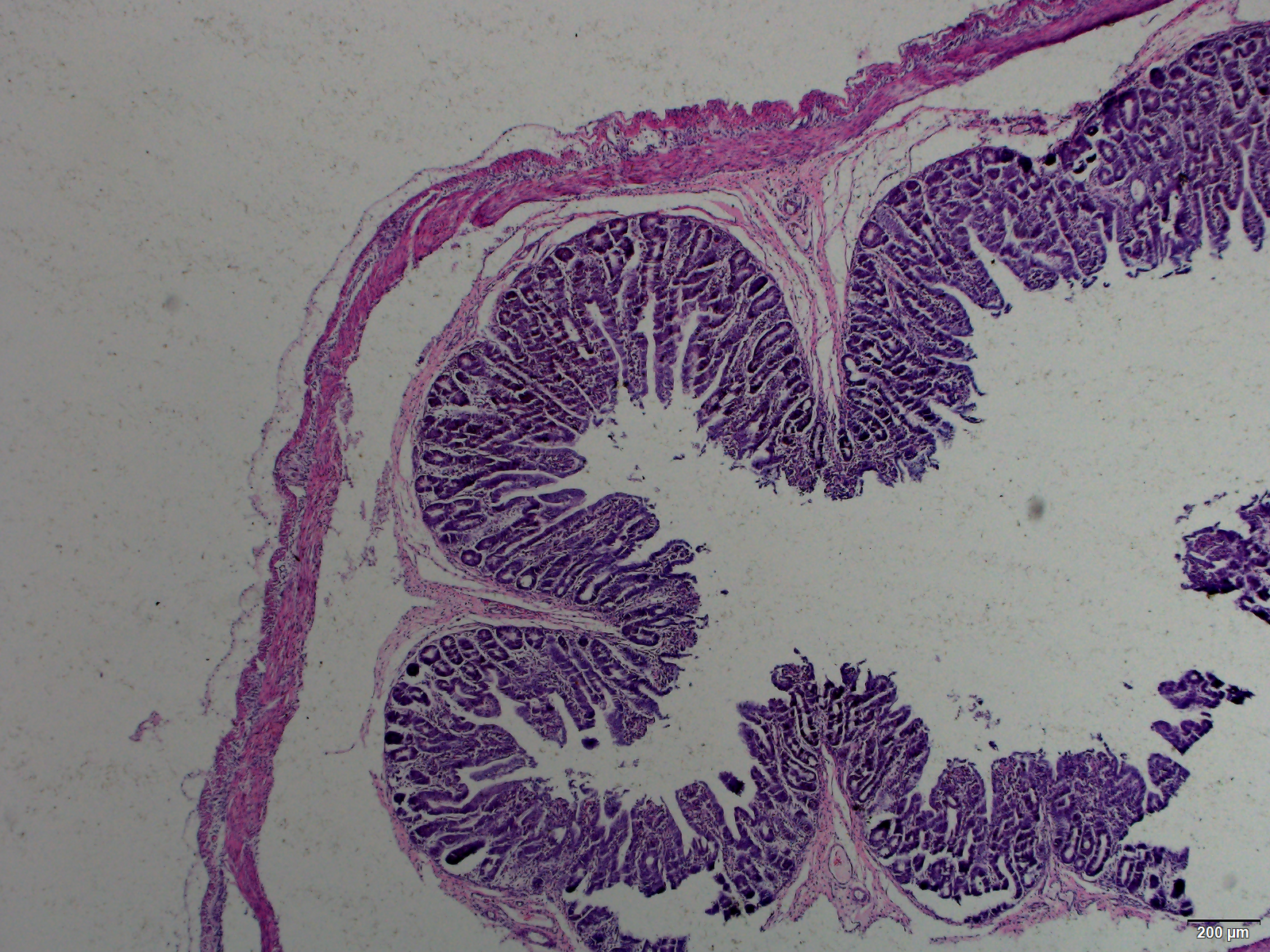

Supplement: Supplementary file 7 [file DataSheet_6.zip › Raw data of Microscopy images Figure 1A/PEDV-Duodenum.tif]

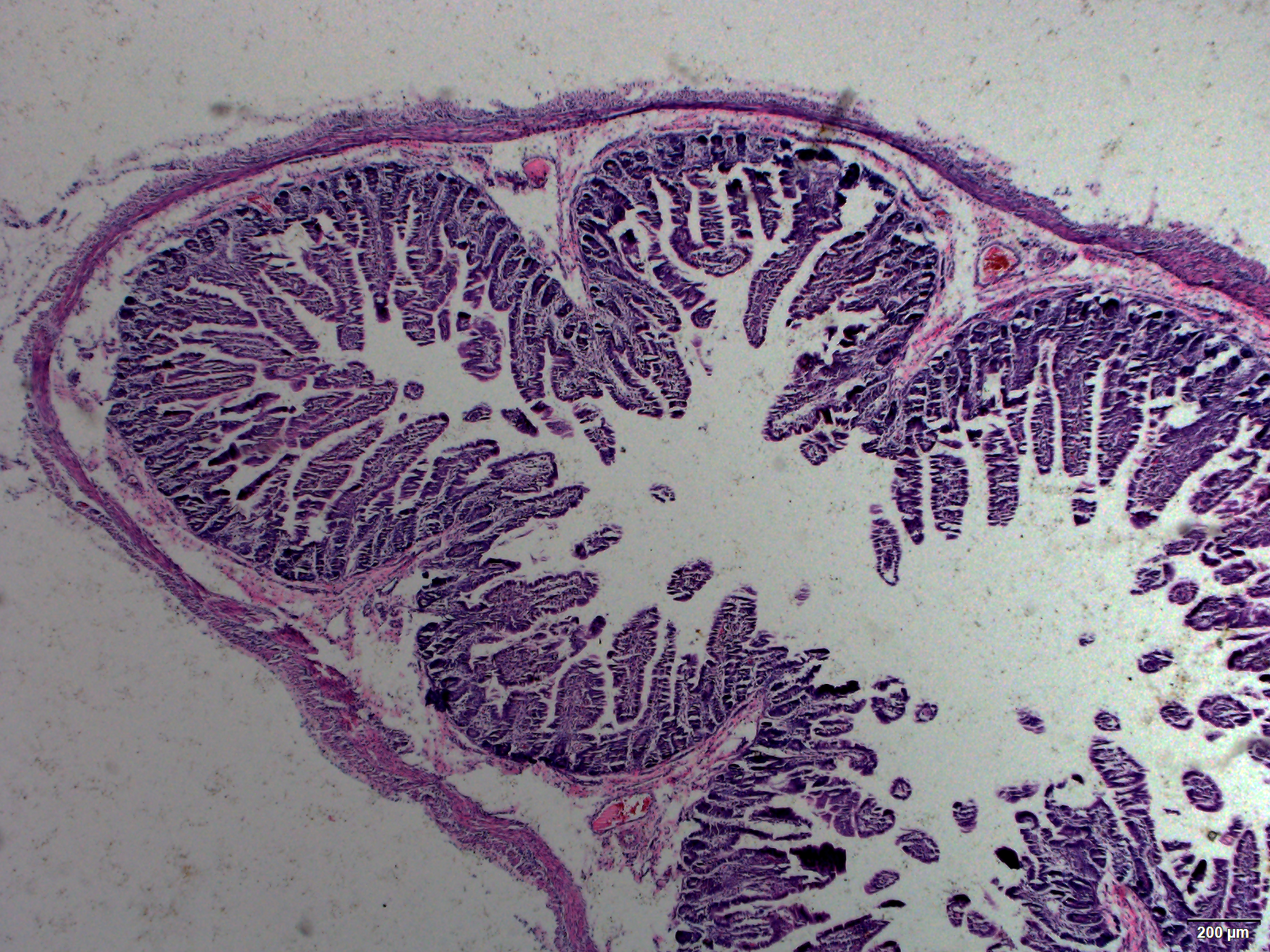

Supplement: Supplementary file 7 [file DataSheet_6.zip › Raw data of Microscopy images Figure 1A/PEDV-Ileum.tif]

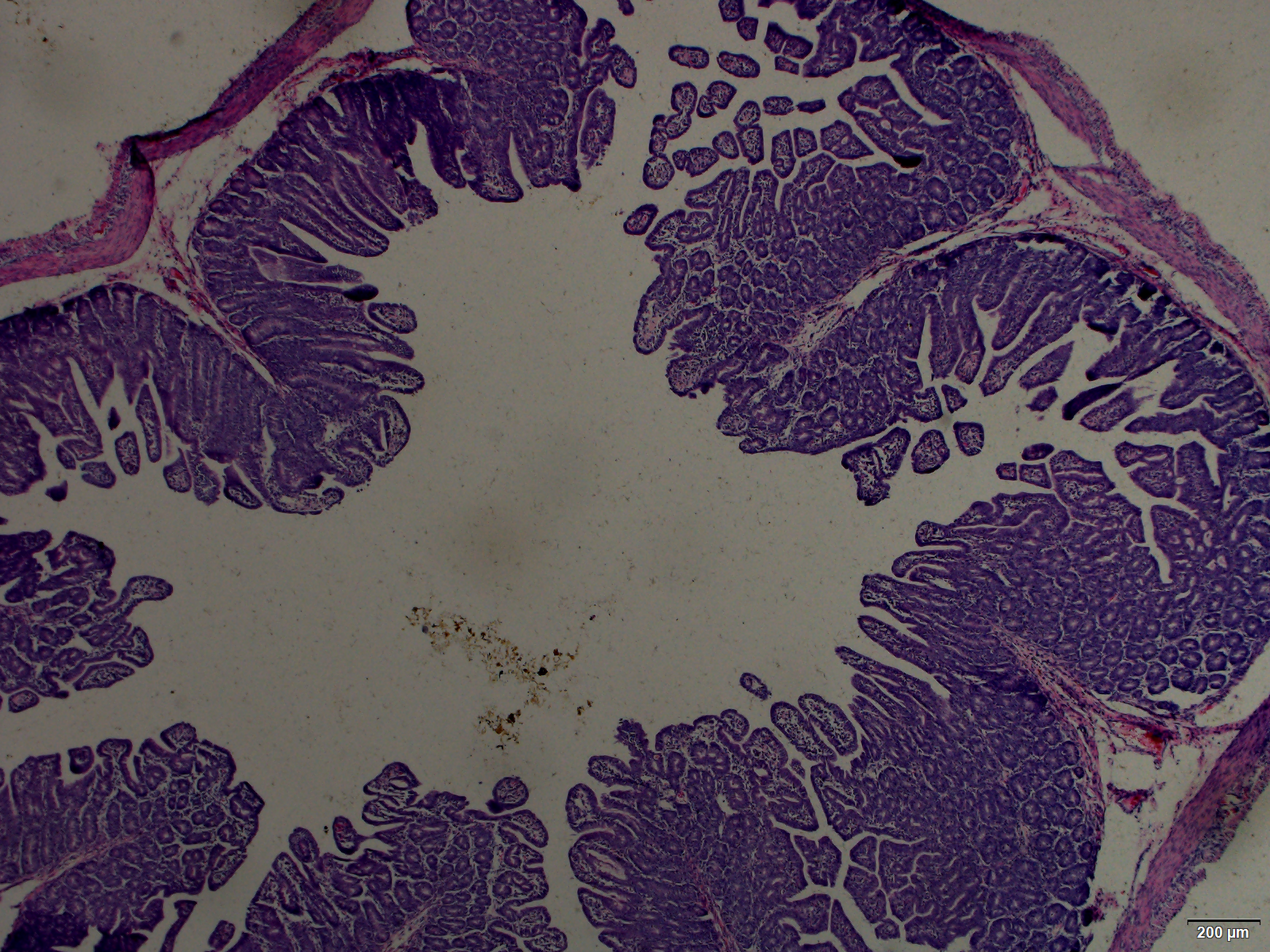

Supplement: Supplementary file 7 [file DataSheet_6.zip › Raw data of Microscopy images Figure 1A/PEDV-Jejunum.tif]

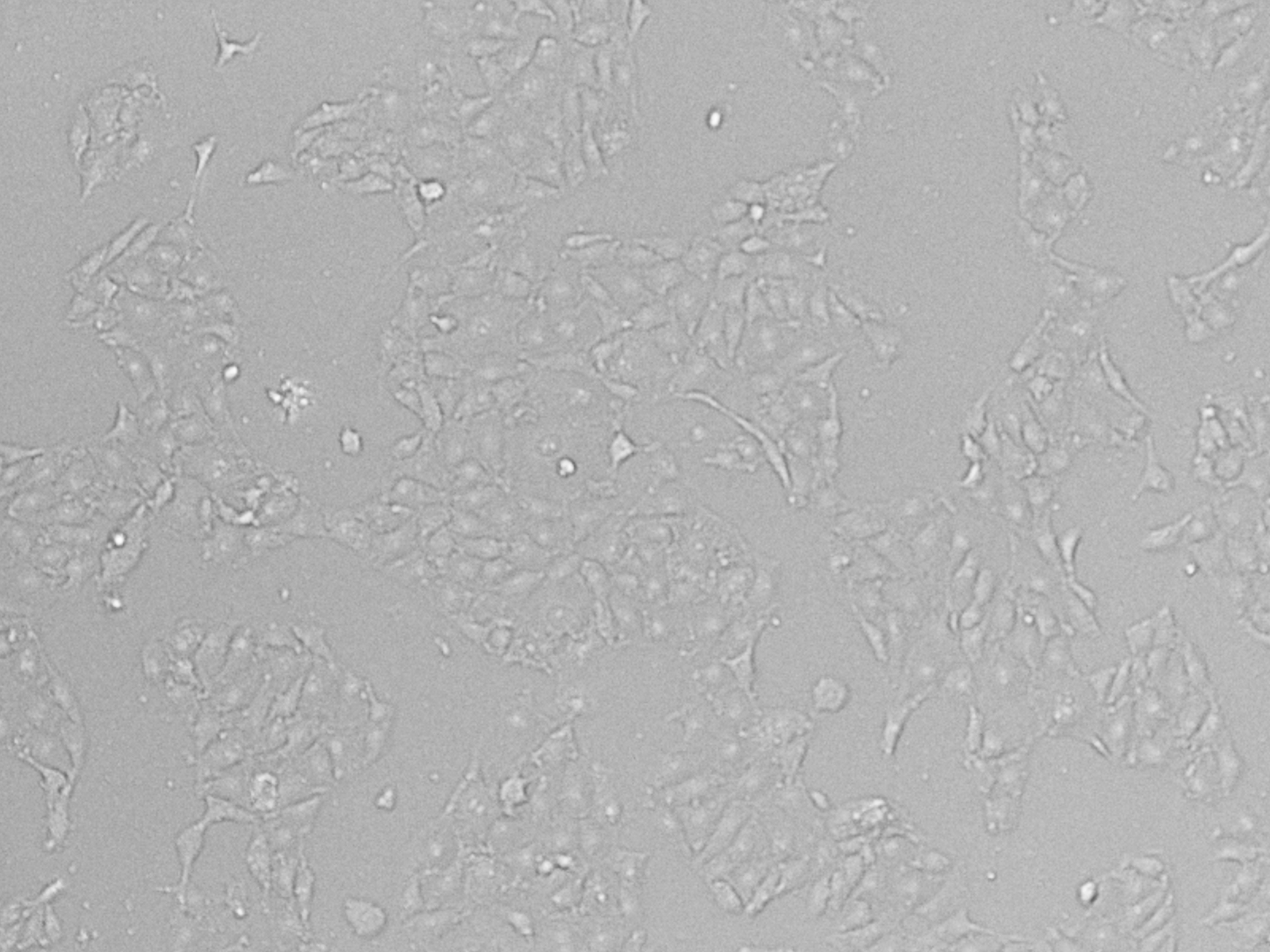

Supplement: Supplementary file 8 [file DataSheet_7.zip › Raw data of Microscopy images Figure 1D/0h.tif]

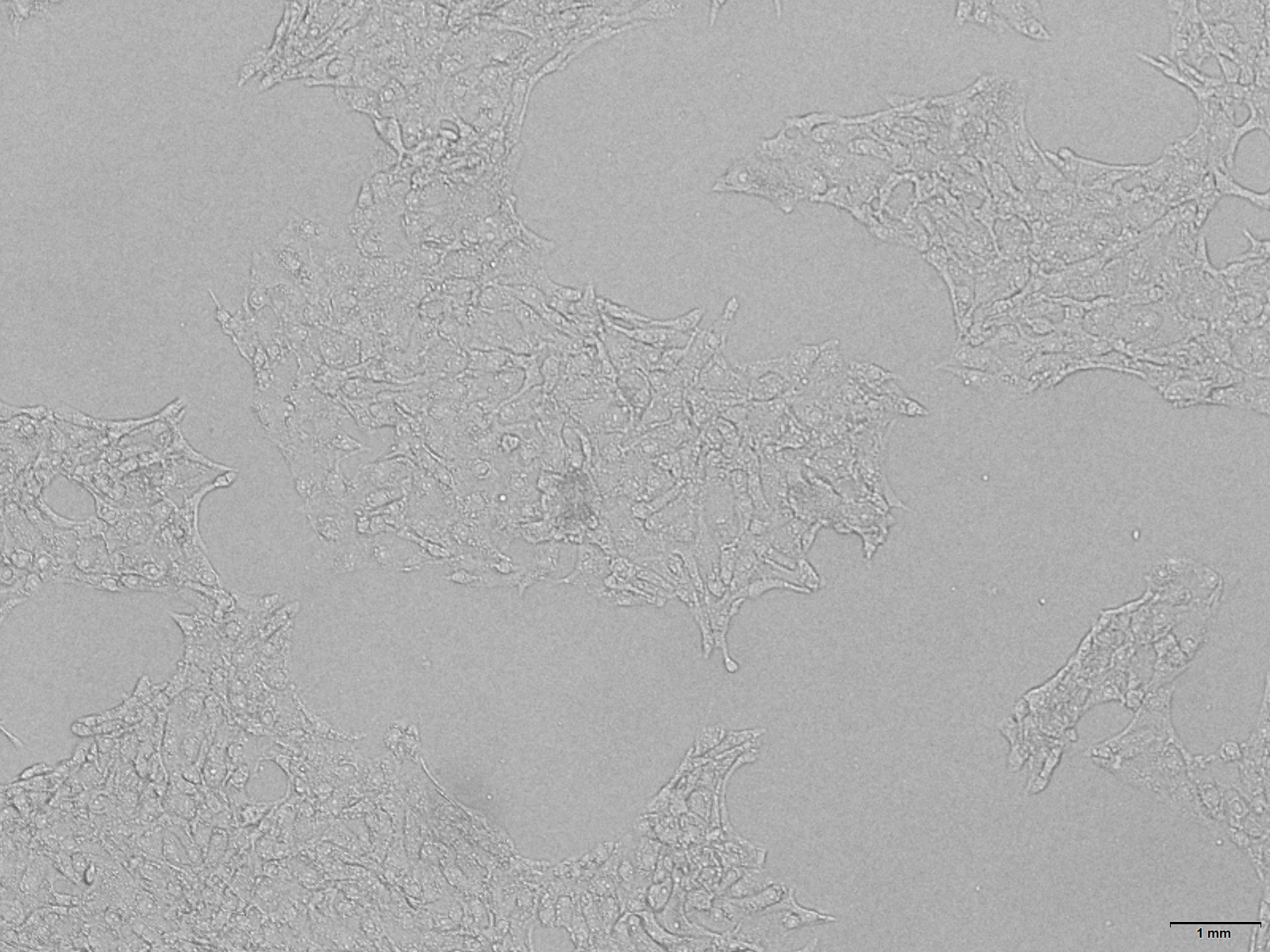

Supplement: Supplementary file 8 [file DataSheet_7.zip › Raw data of Microscopy images Figure 1D/24h.tif]

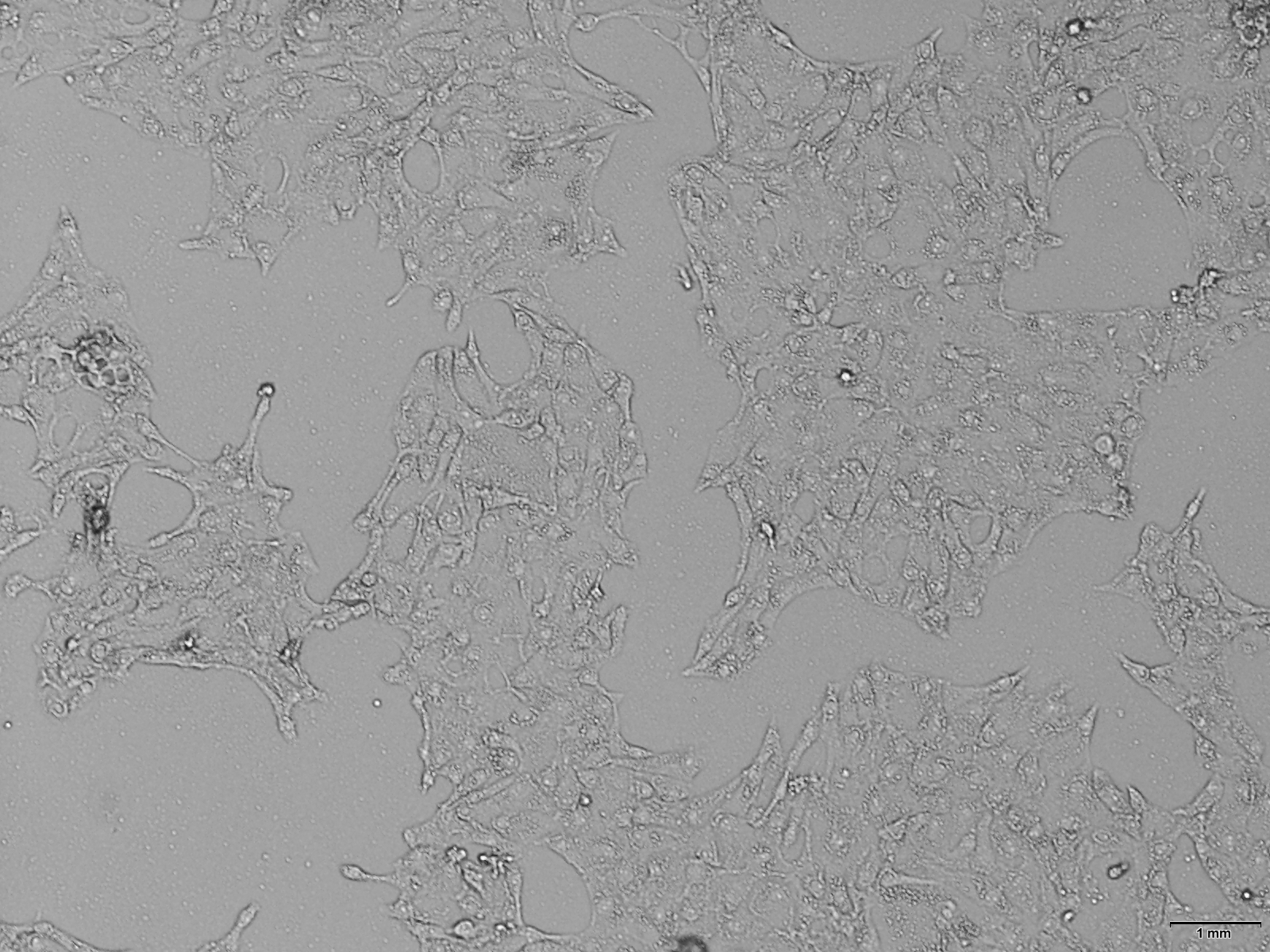

Supplement: Supplementary file 8 [file DataSheet_7.zip › Raw data of Microscopy images Figure 1D/48h.tif]

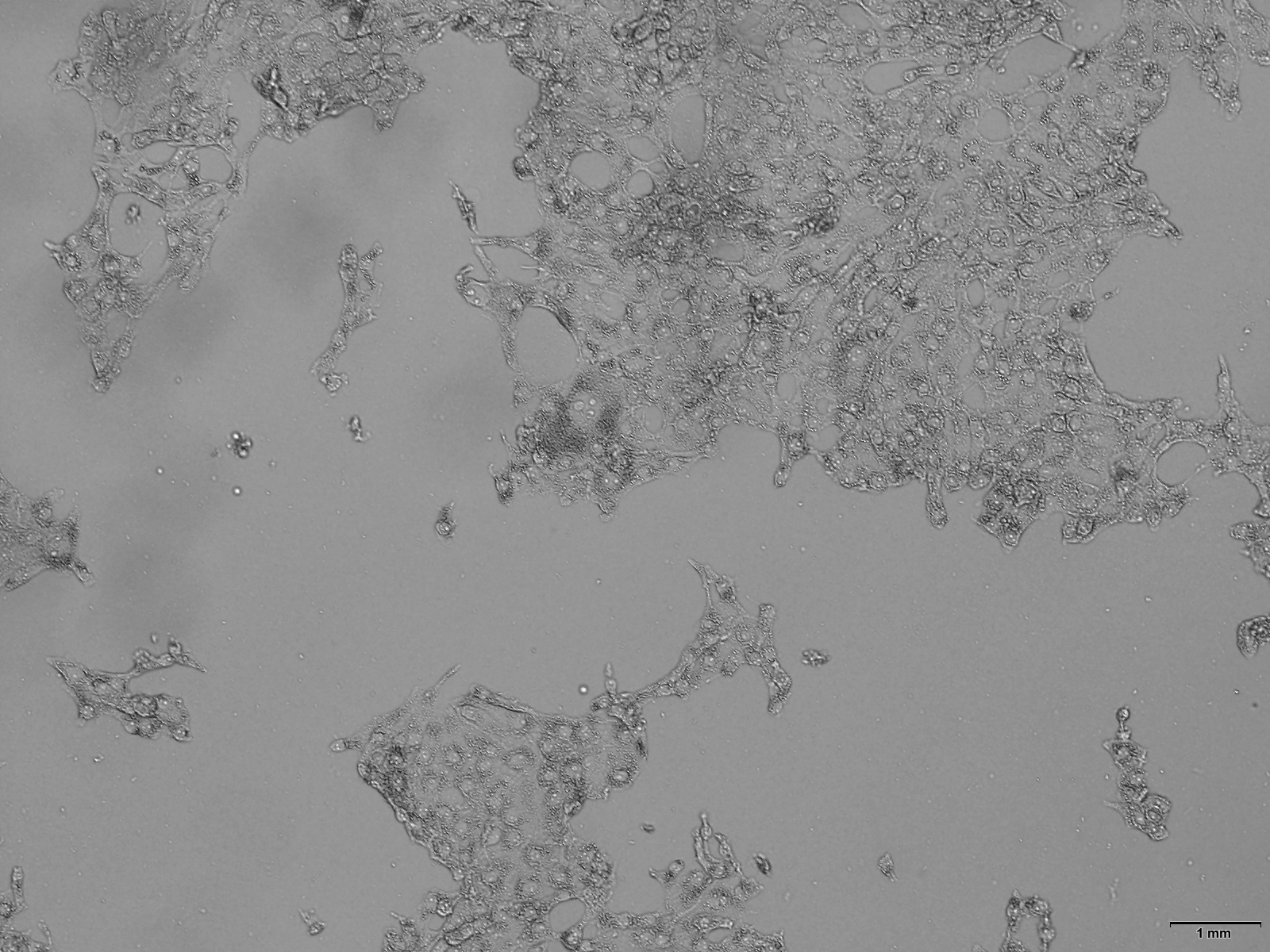

Supplement: Supplementary file 8 [file DataSheet_7.zip › Raw data of Microscopy images Figure 1D/72h.tif]

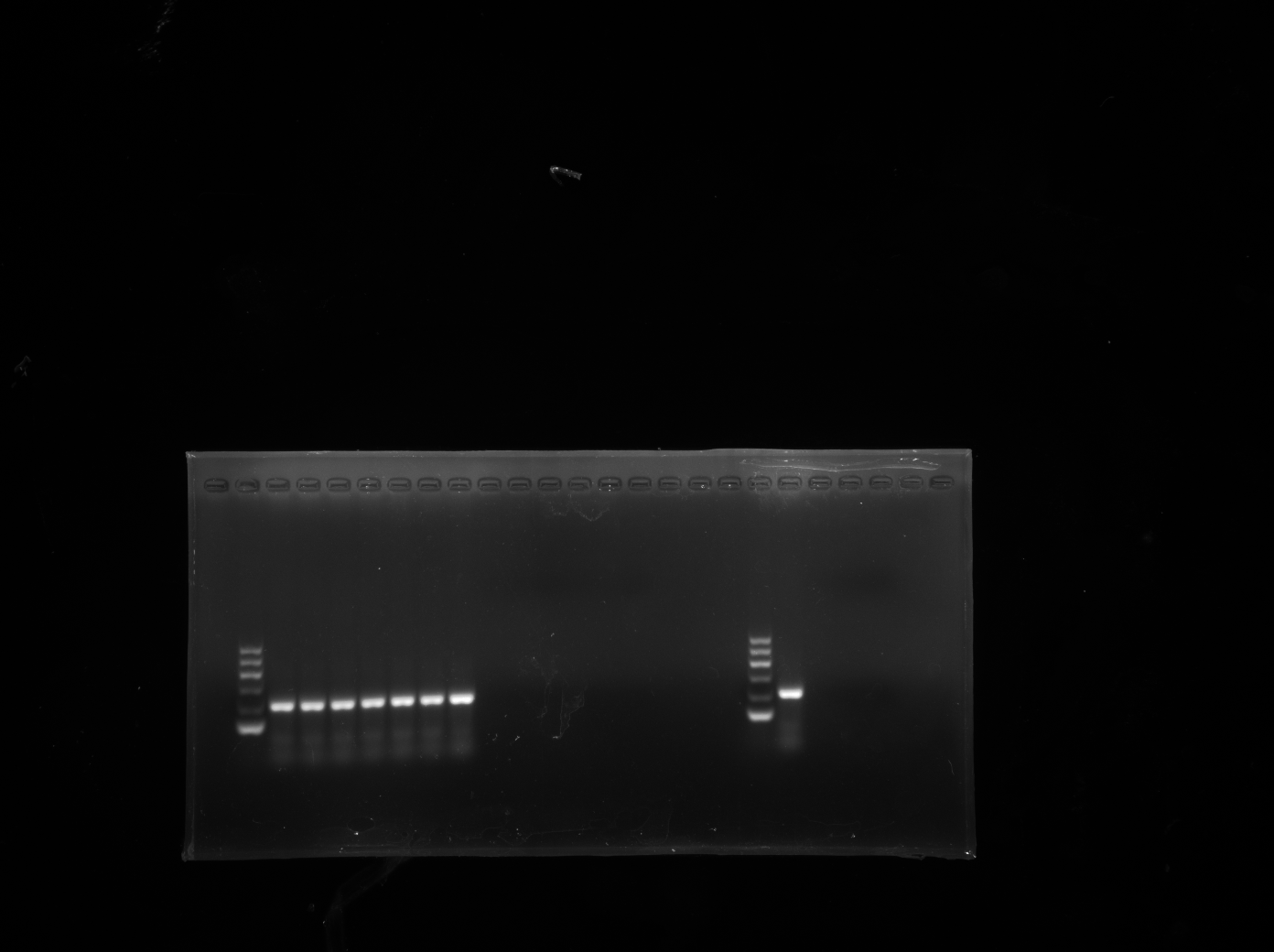

Supplement: Supplementary file 9 [file DataSheet_8.zip › Raw data of Gel images/Supplymentary Figure 1/Supplymentary Figure 1.tif]

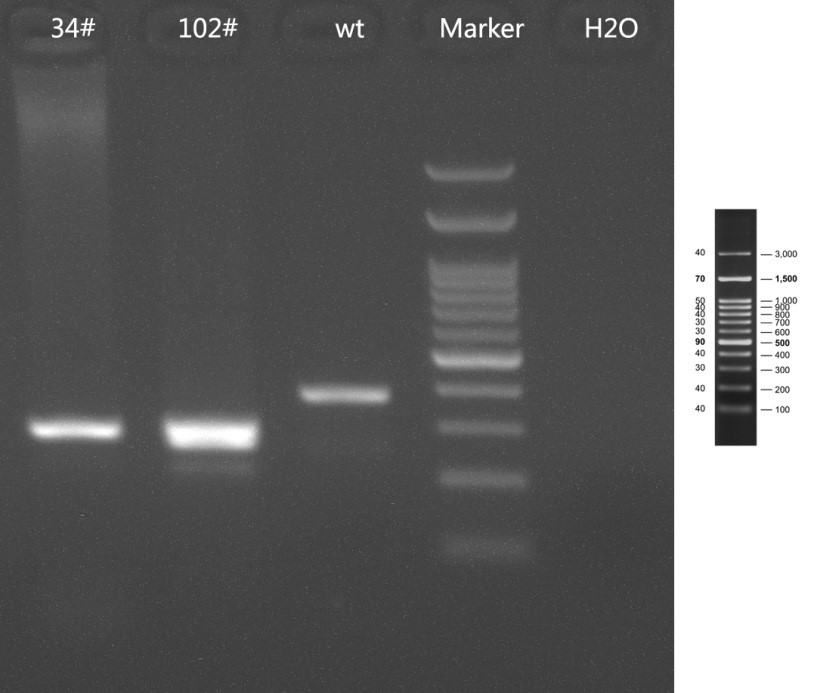

Supplement: Supplementary file 9 [file DataSheet_8.zip › Raw data of Gel images/Supplymentary Figure 1/Supplymentary Figure 2.jpg]
