# Supplementary material for: Hypomethylated interferon regulatory factor 8 recruits activating protein-2α to attenuate porcine epidemic diarrhea virus infection in porcine jejunum
Source: Front Immunol. 2023 Aug 1;14:1187144. doi: 10.3389/fimmu.2023.1187144 (PMC10427914; doi:10.3389/fimmu.2023.1187144)

The Western blot bands (related to Figure 1B, 1D)

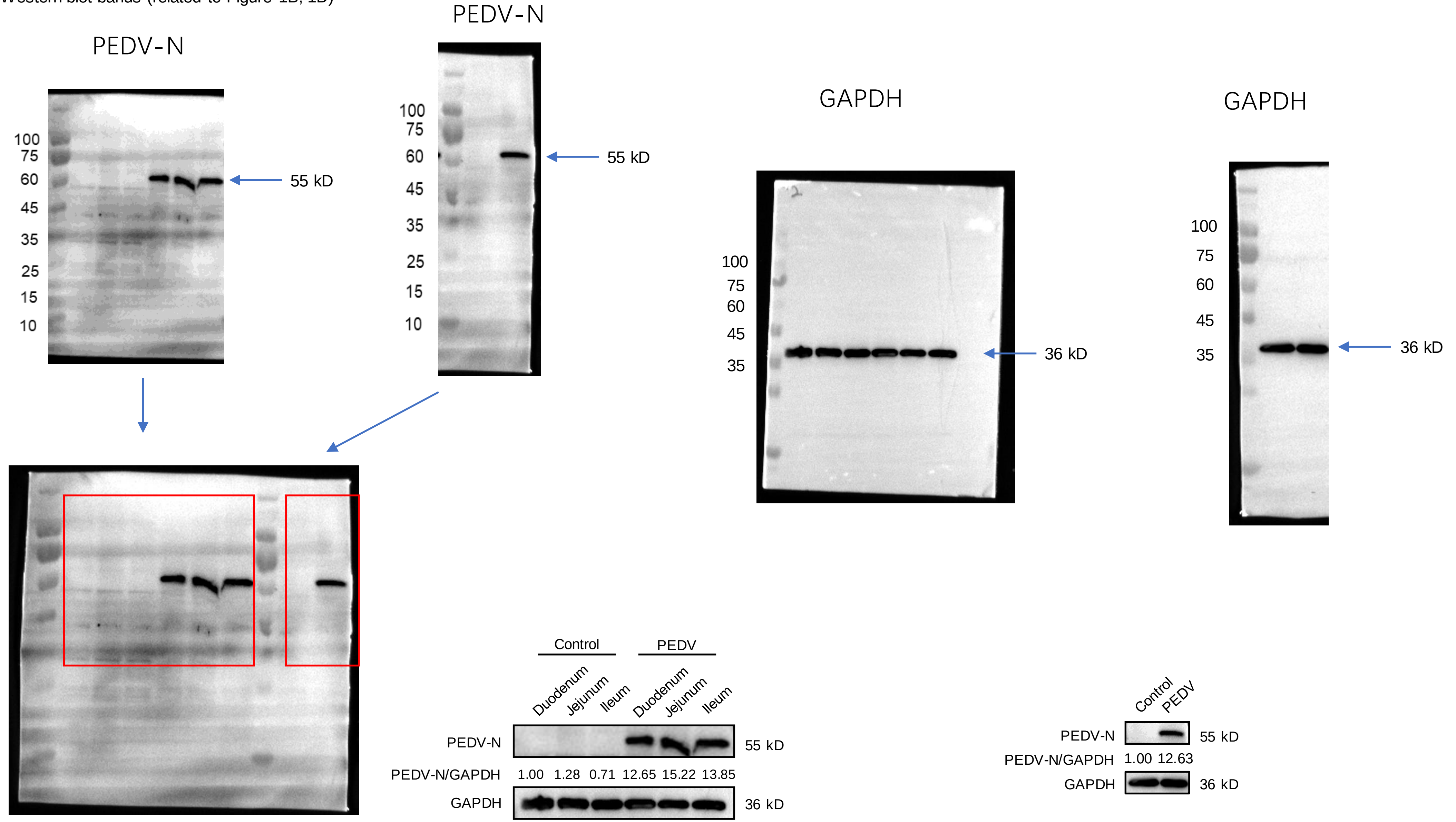

The Western blot bands (related to Figure 2C, 2H)

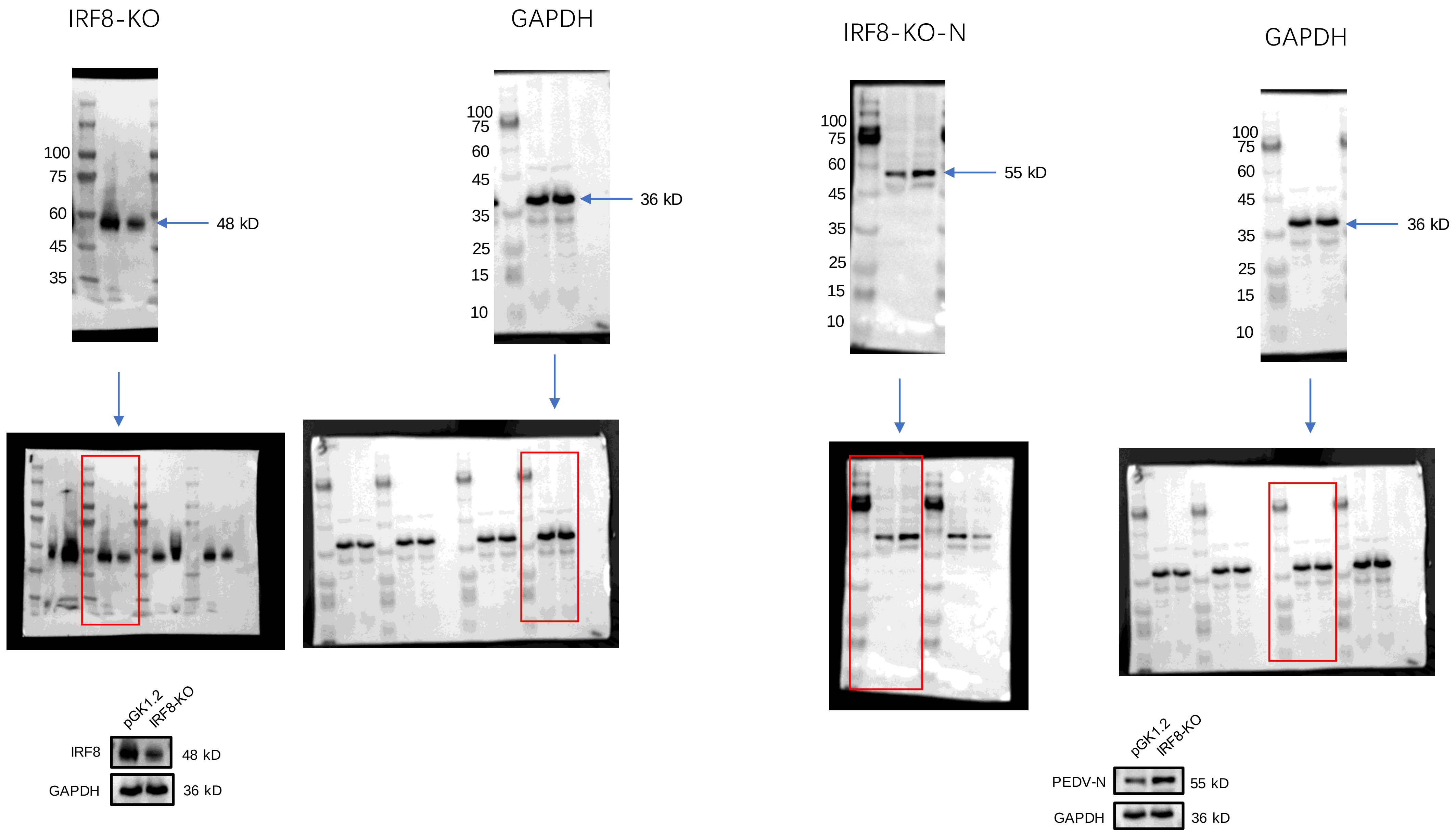

The Western blot bands (related to Figure 7B, 7D)

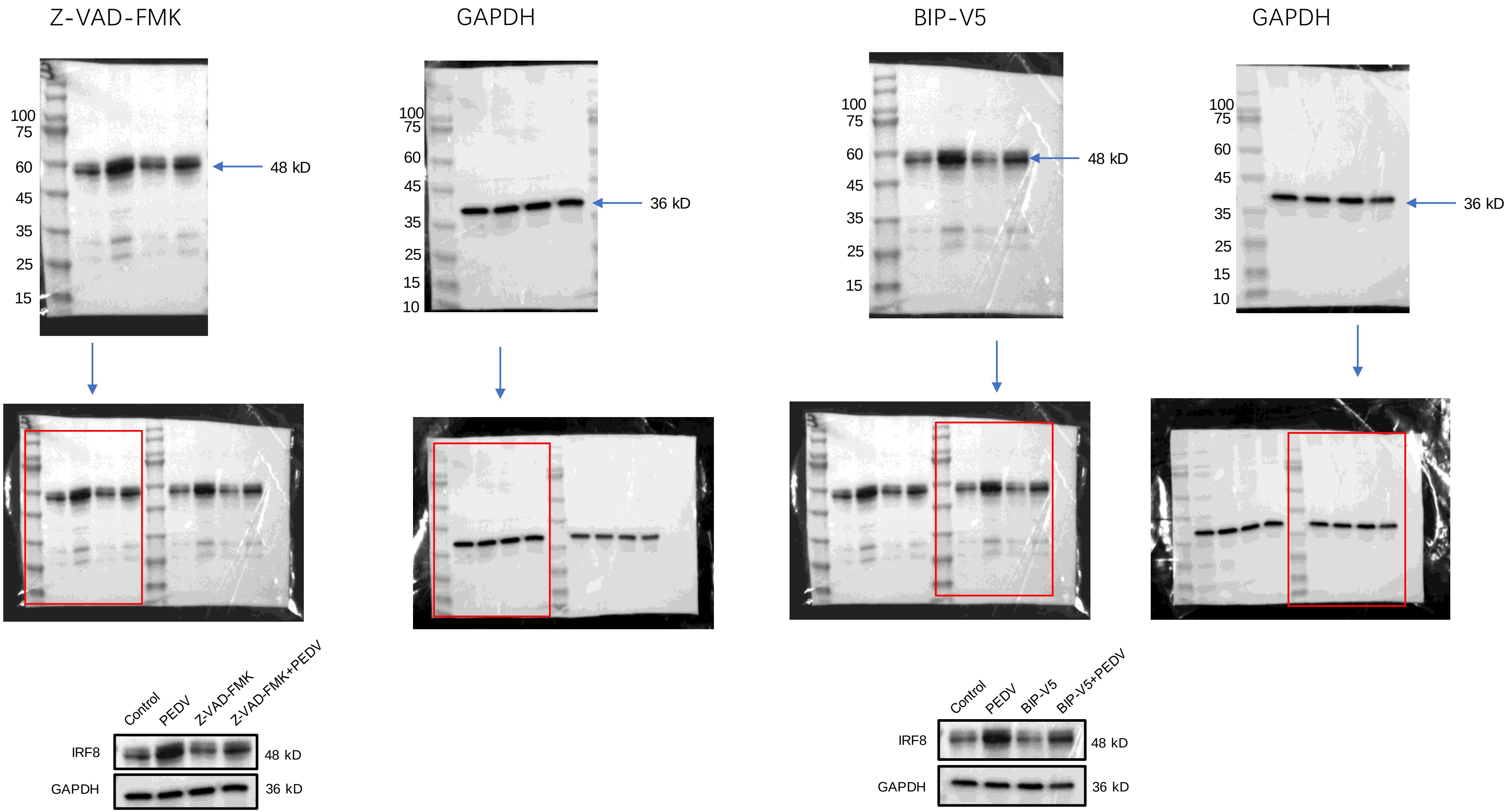

The Western blot bands (related to Figure 7H)

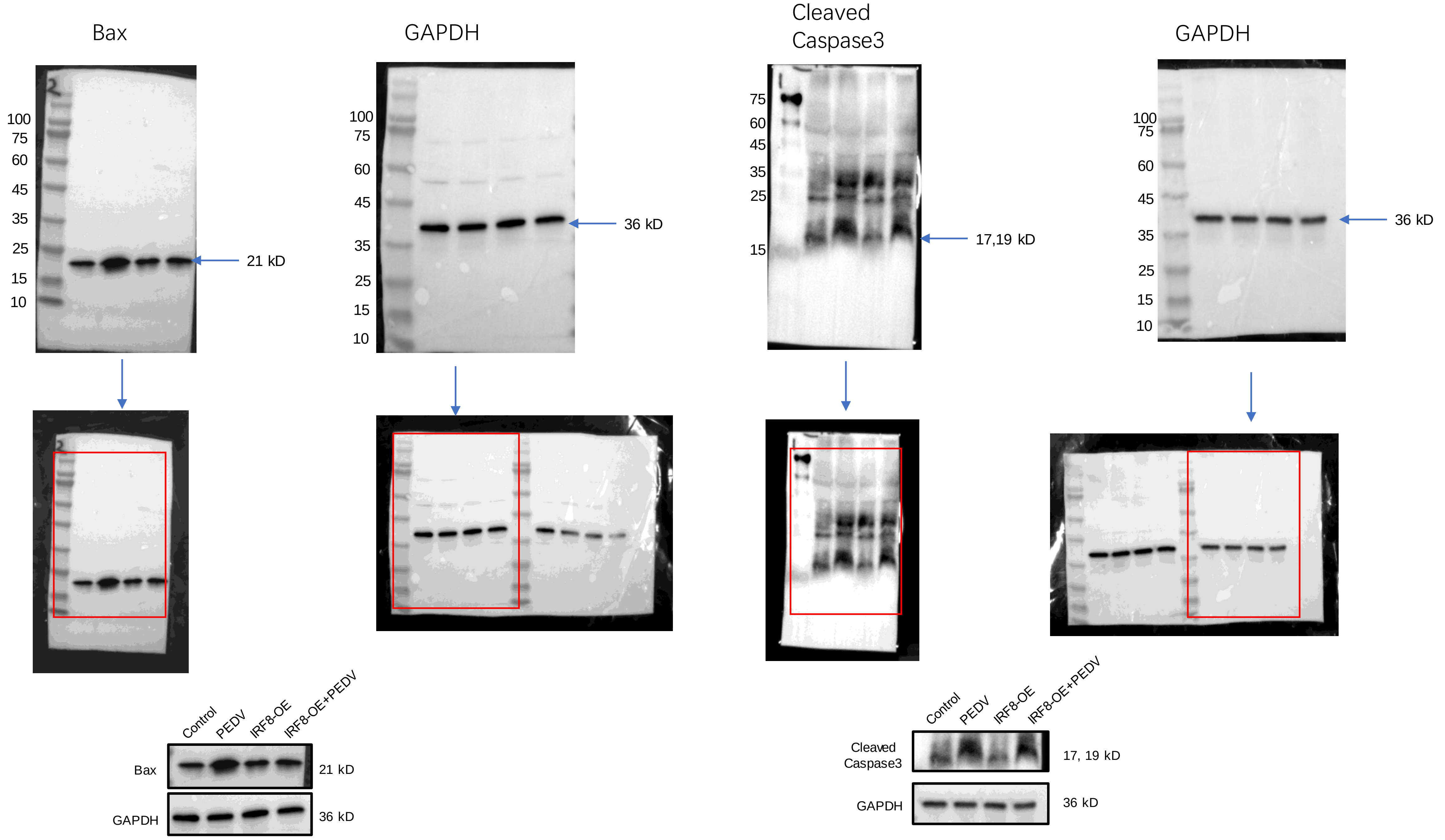

The Western blot bands (related to Figure 7J)

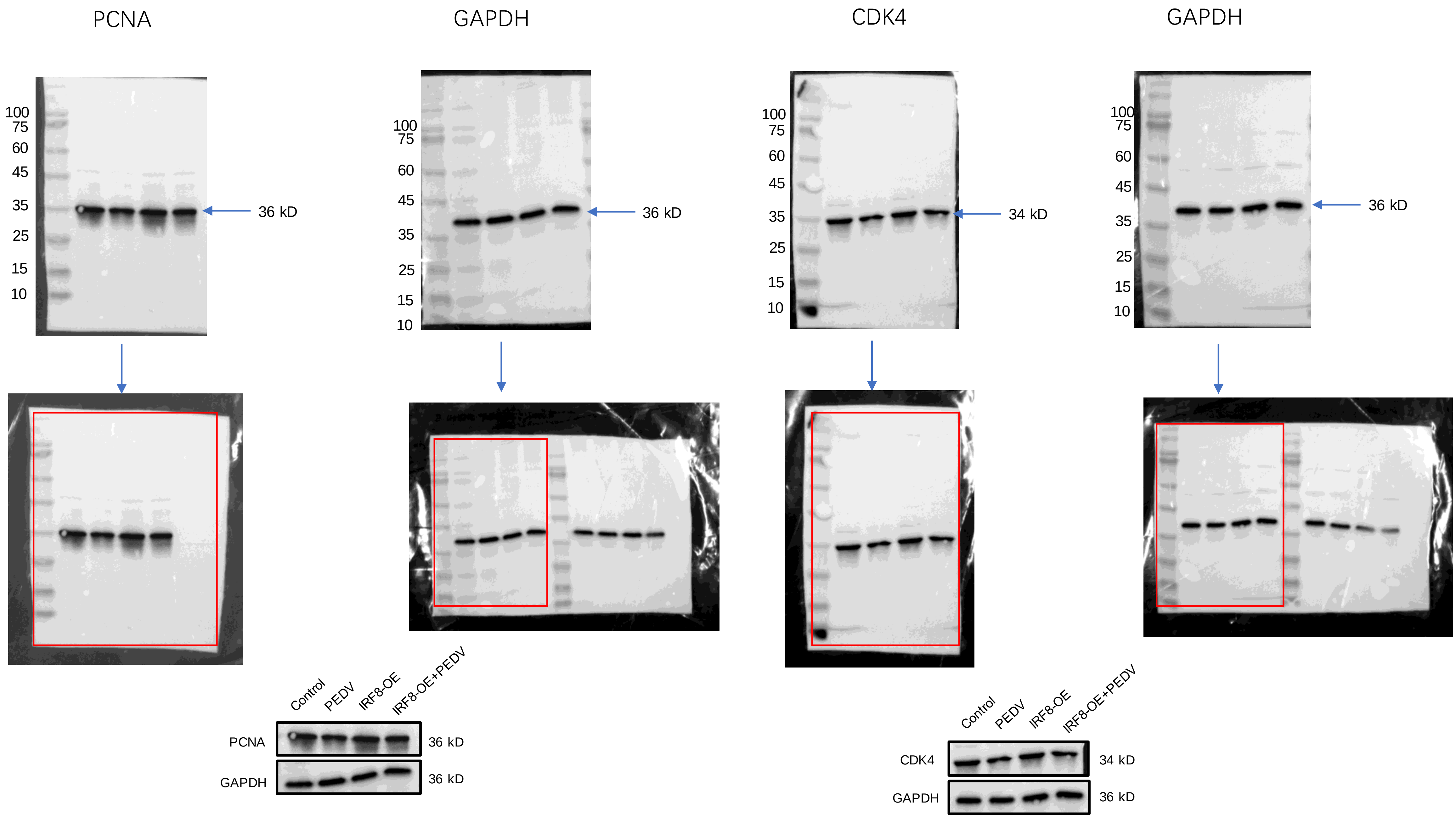

The Western blot bands (related to Figure S2C, S2F)

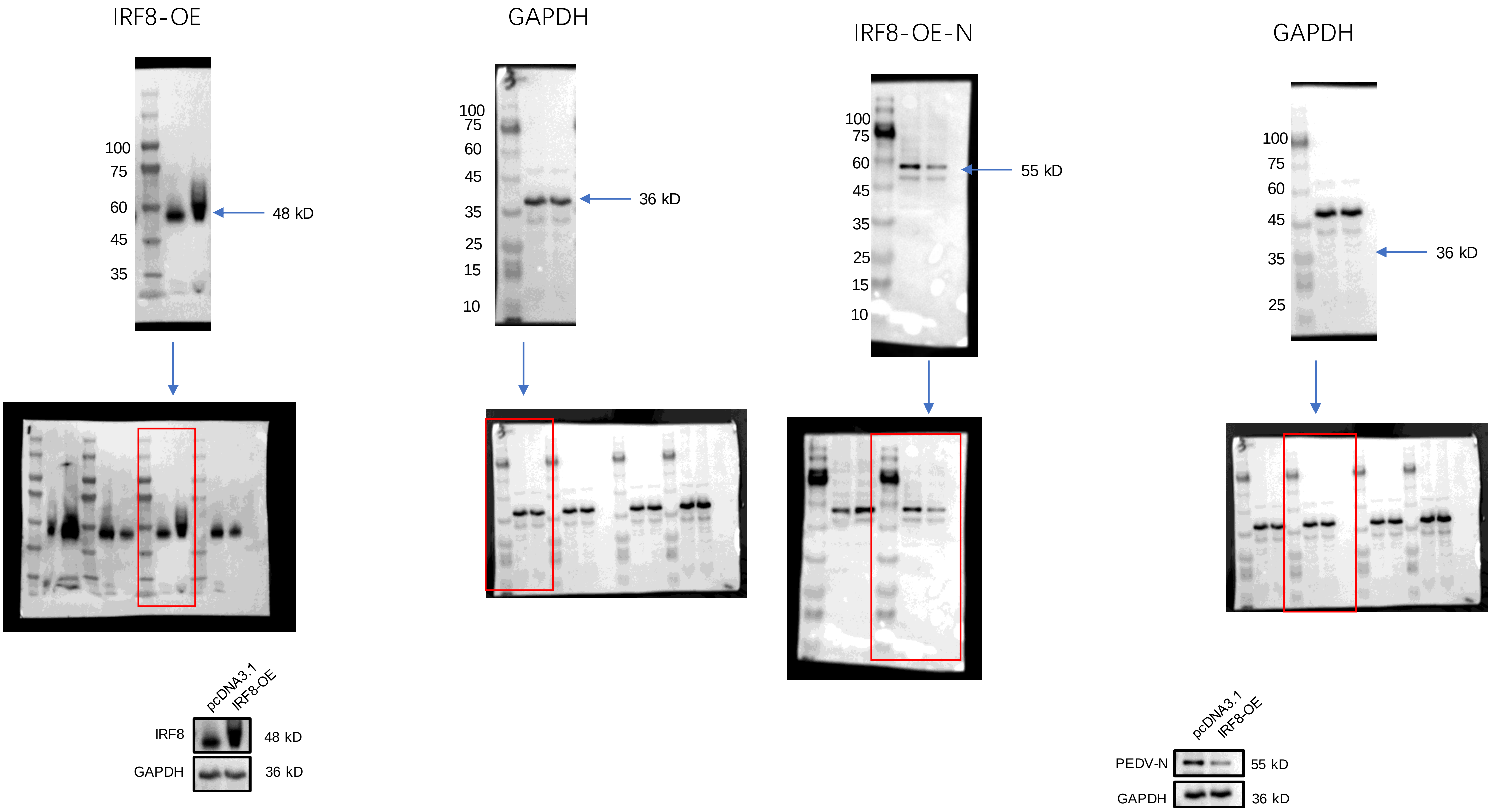

Supplement: Supplementary file 9 [file DataSheet_8.zip › Raw data of Gel images/Western blot imaging/Western blot.pdf]
